# Association between the MCP-1 -2518 A > G (rs1024611) polymorphism and susceptibility to type 2 diabetes mellitus and diabetic nephropathy: a meta-analysis

**DOI:** 10.1186/s12902-023-01514-z

**Published:** 2023-12-04

**Authors:** Wei-wei Chang, Liu Zhang, Li-ying Wen, Yu-jing Tao, Jia-jie Xiong, Xin Tong, Yue-long Jin, Hong Su

**Affiliations:** 1 Department of Epidemiology and Health Statistics, School of Public Health, Wannan Medical College, Wuhu, Anhui 241002 China; 2 Department of Hospital Infection Management Office, Wuhu Hospital of Traditional Chinese Medicine, Wuhu, Anhui 241000 China; 3 Department of Epidemiology and Health Statistics, School of Public Health, Anhui Medical University, Hefei, Anhui 230032 China; 4 Inflammation and Immune Mediated Diseases Laboratory of Anhui Province, No.81 Meishan road, Shushan District, Hefei, Anhui 230031 China

**Keywords:** Monocyte chemoattractant protein-1, Diabetes mellitus, Type 2, Diabetic nephropathy, Meta-analysis

## Abstract

**Background:**

Studies evaluating the association between monocyte chemoattractant protein-1 (MCP-1) -2518 A > G (rs1024611) polymorphism and type 2 diabetes mellitus (T2DM) and diabetic nephropathy (DN) are contradictory. The present study aims to provide a comprehensive assessment and more reliable estimation of the relationship between the MCP-1 rs1024611 polymorphism and T2DM and DN risk.

**Methods:**

Eligible articles were retrieved from the PubMed, Web of Science, EMBASE, Cochrane, and China National Knowledge Infrastructure databases. The effect summary odds ratios (ORs) and 95% confidence intervals (CIs) were obtained to calculate the summary effect size. Heterogeneity was analyzed by subgroup analysis and meta-regression. Publication bias was tested using funnel plots and Egger’s test.

**Results:**

In total, sixteen studies were included. Thirteen studies involving 2,363 patients with T2DM and 4,650 healthy controls found no significant association between the MCP-1 rs1024611 polymorphism and T2DM in the overall population. Ethnicity stratification found an association between the GG + GA genotype and decreased T2DM risk in Caucasians (*OR* = 0.79, 95% CI: 0.66–0.93, *P* = 0.006; *P*_*Q*_ = 0.372). No significant risks were found in the Asian population for any genetic models. Seven studies found an association between the GG + GA genotype and DN risk in the Asian population (*OR* = 1.37, 95% CI: 1.11–1.71, *P* = 0.004, *P*_*Q*_ = 0.222). No significant risks were found in the Caucasian population with any genetic models. There were no statistically significant differences in genotype distribution between patients with T2DM and DN in Asians or Caucasians. Meta-regression revealed that genotyping method was a major driver of heterogeneity in five genetic models (GG + GA vs. AA: *P* = 0.032; GG vs. GA + AA: *P* = 0.028; GG vs. AA: *P* = 0.035; GG vs. GA: *P* = 0.041; G vs. A: *P* = 0.041).

**Conclusion:**

The MCP-1 rs1024611 polymorphism is associated with susceptibility to T2DM in Caucasians and DN in Asians. Larger, well-designed cohort studies are needed in the future to verify this association.

**Supplementary Information:**

The online version contains supplementary material available at 10.1186/s12902-023-01514-z.

## Background

Type 2 diabetes mellitus (T2DM) is a major global health problem and its global prevalence is increasing annually [1]. Approximately 536.6 million people (global prevalence: 10.5%) aged 20–79 years lived with DM in 2021, which was projected to rise to 783.2 million (global prevalence: 12.2%) by 2045 [2]. Microangiopathy is a specific pathology of T2DM, including diabetic nephropathy (DN), diabetic neuropathy, and diabetic retinopathy. DN, as a gradually developing kidney disease, is a critical chronic complication of T2DM and the main cause of end-stage renal disease [3]. DN can be difficult to reverse, thus causing cardiovascular and cerebrovascular disease and presenting an enormous economic burden to society. Given the progression of diabetes and the lack of a clear cure, elucidation of the molecular mechanisms of T2DM and DN is urgently needed to prevent and treat diabetes.

The etiology and pathogenesis of T2DM and DN have not been fully elucidated. Recently, the theory of inflammatory injury has received much attention. Evidence from many clinical and experimental studies shows that T2DM and DN are natural, immune, chronic, low-grade, inflammatory diseases [4–6]. Monocyte chemoattractant protein-1 (MCP-1) is an important chemokine that participates in inflammatory process regulation by activating monocytes and macrophage accumulation in damaged tissues [7]. Several studies have shown that MCP-1 plays a crucial role in inflammatory and immune diseases [8, 9]. An early study showed that serum MCP-1 levels were positively correlated with urinary albumin excretion and the degree of renal damage [10]. These studies suggest that MCP-1 is involved in glomerular injury and T2DM and DN occurrence and development. Single-nucleotide polymorphisms (SNPs), the most common human genetic variation, can affect gene expression and be used to predict disease risk [11]; the MCP-1 -2518 A > G (rs1024611) gene mutation stimulated MCP-1 expression following the inflammatory response [12]. In addition, Zakharyan et al. reported that GG genotype carriers had the highest MCP-1 level compared with AA genotype and AG genotype carriers [13]. These studies indicate that the MCP-1 rs1024611 polymorphism may be associated with T2DM and DN risk.

In recent decades, some case‒control studies have investigated the relationship between the MCP-1 rs1024611 polymorphism and the risk of DN or diabetes; however, the results have been inconsistent [14–29]. Studies are generally restricted by sample size but meta-analyses have greater testing power and produce comprehensive and reliable conclusions. Although several meta-analyses have reported an association between the MCP-1 rs1024611 polymorphism and DN or diabetes risk [30–32], the results were inconsistent; this may have several causes. First, when exploring the correlation between the MCP-1 rs1024611 polymorphism and DN risk, selection of the control group was often not uniform, including patients with diabetes and healthy individuals [31, 32]. Second, in different meta-analyses, inclusion for diabetes differed. For example, Zhang et al. included T1DM and T2DM, while others only included T2DM [30]. Another recent meta-analysis was conducted with a limited number of studies [24]. Therefore, we conducted a comprehensive meta-analysis of all available case‒control studies, aiming to provide reliable evidence for the associations between the rs1024611 polymorphism and DN or T2DM risk using three models: (1) T2DM vs. healthy control; (2) DN vs. healthy control; and (3) DN vs. T2DM.

## Material and methods

### Literature search strategy

This meta-analysis followed the PRISMA guidelines for systematic reviews [33]. Searches were performed using the PubMed, Web of Science, EMBASE, Cochrane, and the China National Knowledge Infrastructure (CNKI) databases up to May 2023, including articles in English and Chinese. The following search terms were used: (‘diabetes mellitus’ or ‘DM’ or ‘nephropathy’ or ‘DN’ or ‘diabetes’) and (‘MCP-1’ or ‘rs1024611’ or ‘monocyte chemoattractant protein-1’ or ‘CCL2’) and (‘polymorphism’ or ‘genotype’ or ‘mutation’). The comprehensive search strategies for different databases are listed in Table S1. A manual search was conducted for the relevant references cited in these articles, and if more information was needed, we contacted the corresponding authors. When the same population was included in multiple publications, only the latest or complete study was included.

### Inclusion and exclusion criteria

Studies meeting all the following criteria were included: (1) observational studies (cohort, case–control, and cross–sectional); (2) at least two comparison groups (T2DM group vs. DN or healthy control group); (3) the distribution of the genotypes in control group and T2DM group (when we compared T2DM group with DN group) was in Hardy–Weinberg equilibrium (HWE); and (4) providing the genotype distribution frequency of the DN and control groups (or T2DM or healthy control groups), or the possibility of calculating this using the literature. Exclusion criteria included: (1) duplicate publications and (2) reviews, case reports, and meta-analyses.

The PECO format was used [33], as follows: (P) patients with T2DM or DN; (E) distribution of the allelic variants of the SNP at MCP-1 rs1024611; (C) control groups; and (O) risk of developing T2DM or DN, as measured by OR.

### Data extraction and quality evaluation

Data extraction was performed independently by two researchers based on the inclusion and exclusion criteria. If any information was missing from the selected articles, we contacted the corresponding authors. The extracted information included the first author, year of publication, study design, country and ethnicity of subjects, HWE in the control group, and the number of genotypes in the case and control groups. The studies included in the analysis were scored according to the standard Newcastle–Ottawa Scale (NOS) [34]. Studies with a score ≥ 7 were considered high-quality.

### Statistical analysis

Stata version 12.0 software was used for all statistical analyses. A chi-square test was used to examine whether the distribution of genotypes among the control and T2DM groups (compared to the T2DM and DN groups) within each study was in HWE. The odds ratios (ORs) and 95% confidence intervals (CIs) were used to calculate the summary effect size and assess the association between the MCP-1 rs1024611 polymorphism and T2DM or DN susceptibility. Pooled ORs were calculated using the Z test for the dominant (GG + GA vs. AA), recessive (GG vs. GA + AA ), allele contrast (G vs. A), homozygote (GG vs. AA), and heterozygote models (GG vs. GA). *P* < 0.05 was considered statistically significant. Subgroup analyses and meta-regression for the study population, genotyping method, comorbid chronic disease, and age- and sex-adjustment were performed to identify possible sources of heterogeneity.

Cochran’s (Q) and *I*^*2*^ tests were used to evaluate statistical heterogeneity [35]. The random-effects (RE) model (if *P*_Q_ < 0.10 or *I*^*2*^ > 50%) was used to calculate the OR and 95% CIs; otherwise, the fixed-effects (FE) model was used to calculate combined effect estimates [36]. A sensitivity analysis was conducted to describe the robustness of our findings. Possible publication bias was tested using funnel plots and Egger’s test.

## Results

### Study characteristics

**Fig. 1:**
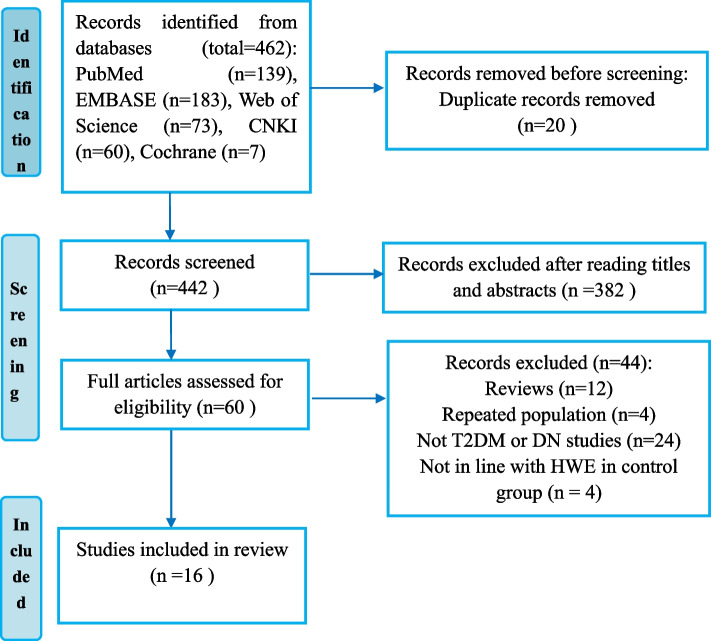
Flow chart illustrating the trial structure. Abbreviations: CNKI, China National Knowledge Infrastructure; T2DM, type 2 diabetes mellitus; DN, diabetic nephropathy; HWE, Hardy–Weinberg equilibrium

A total of 462 studies were obtained through systematic searches. After reading the title, abstract and full text, 446 articles were excluded and the remaining 16 articles were included in the meta-analysis [14–29]. A flow chart of the study selection process is shown in Fig. 1. Thirteen studies were based on Asian populations (three studies in Korea, nine studies in China, and one study in India) [14, 15, 17–19, 21–24, 26–29]; another three studies involved Caucasian populations (one in Turkey, one in Germany, and one in Poland) [16, 20, 25]. Ten studies explored the association between the MCP-1 rs1024611 polymorphism and DN risk. Data extracted from the selected studies are summarized in Table 1 and Table S2.

**Table 1:** Characteristics of studies that provided genotype frequencies evaluating the effects of MCP-1 rs1024611 polymorphism on DN and T2DM risk

No	First author, year	Country	Ethnicity	Sample size^a^	T2DM^b^	DN^c^	Control^d^	HWE(T2DM/ Control)	NOS score
1	Simeoni, 2004 [25]	Germany	Caucasians	632/-/2568	363/222/47	-	1335/1043/190	-/0.482	8
2	Joo, 2007 [19]	Korea	Asians	169/164/-	23/78/68	26/73/65	-	0.933/-	7
3	Moon, 2007 [23]	Korea	Asians	112/112/230	11/50/51	16/61/35	41/102/87	0.804/0.249	8
4	Chen, 2007 [15]	China (Hunan)	Asians	86/94/102	24/40/22	24/44/26	31/47/24	0.521/0.454	6
5	Karadeniz, 2010 [20]	Turkey	Caucasians	43/43/105	26/17/0	24/19/0	49/44/12	0.332/0.659	7
6	Wu, 2011 [28]	China (Tianjin)	Asians	56/56/50	5/25/26	13/25/18	9/22/19	0.771/0.556	7
7	Jing, 2011 [18]	China (Jiangsu)	Asians	416/-/416	50/274/92	-	67/212/137	-/0.318	8
8	Jeoh, 2013 [17]	Korea	Asians	399/191/-	50/349*	28/163*	**-**	**-**	7
9	Grzegorzewska, 2014 [16]	Poland	Caucasians	-/222/437	-	104/97/21	225/177/35	-/0.982	8
10	Raina, 2021 [24]	India	Asians	444/354/515	210/204/30	138/171/45	238/236/41	0.069/0.095	8
11	Xu, 2015 [29]	China (Hubei)	Asians	50/-/50	16/20/14	-	18/19/13	-/0.100	7
12	Ma, 2016 [22]	China (Zhejiang)	Asians	208/-/209	66/102/40	-	46/93/70	-/0.156	8
13	Ma, 2017 [21]	China (Gansu)	Asians	30/-/69	7/13/10	-	13/36/20	-/0.652	7
14	Su, 2018 [26]	China (Hebei)	Asians	135/-/149	18/62/55	-	26/66/57	-/0.688	8
15	Wang, 2019 [27]	China (Jiangsu)	Asians	52/60/78	18/22/12	10/25/25	30/32/16	0.304/0.179	7
16	Cheng, 2019 [14]	China (Zhejiang)	Asians	99/-/109	21/51/27	-	16/54/39	-/0.699	7

*Abbreviations: T2DM* Type 2 diabetes mellitus, *DN* Diabetic nephropathy, *HWE* Hardy–Weinberg equilibrium, *NOS* Newcastle–Ottawa scale

- Not available

* Total number of AG + GG

^a^ Total participants in the T2DM, DN, and control groups, respectively

^b^ Number of AA, AG, and GG genotypes in the T2DM group

^c^ Number of AA, AG, and GG genotypes in the DN group

^d^Number of AA, AG, and GG genotypes in the control group

### Association between the MCP-1 rs1024611 polymorphism and T2DM risk

Thirteen studies with 2,363 patients with T2DM and 4,650 healthy controls were eligible and used to estimate the relationship between the MCP-1 rs1024611 polymorphism and T2DM. Significant heterogeneity was detected among the overall population in five genetic models (Table 2). There was no significant difference between the MCP-1 rs1024611 polymorphism and T2DM under the RE model (Table 2).

**Table 2:** Meta-analysis of the association between the MCP-1 rs1024611polymorphism and T2DM risk (T2DM vs. healthy control)

Genetic variant	Study group	No. studies	Heterogeneity test		Association test (FE model)		Association test (RE model)		Publication bias
			*I*^*2*^	*P* value	OR (95% CI)	*P* value	OR (95% CI)	*P* value	*P* value*
GG + GA vs.AA (dominant)	Overall	13	46.8	0.032	0.92 (0.82–1.03)	0.134	0.98 (0.81–1.20)	0.855	0.110
	Population								
	Asian	11	39.1	0.088	1.04 (0.89–1.21)	0.627	1.07 (0.85–1.34)	0.556	
	Caucasian	2	0.0	0.372	0.79 (0.66–0.93)	0.006	0.79 (0.66–0.93)	0.006	
	Genotyping method								
	PCR–RFLP	10	58.6	0.010	0.90 (0.79–1.03)	0.118	1.00 (0.77–1.23)	0.986	
	ARMS-PCR	2	0.0	0.596	0.98 (0.77–1.25)	0.865	0.98 (0.77–1.25)	0.865	
	TaqMan-PCR	1	-	-	0.76 (0.27–2.16)	0.610	0.76 (0.27–2.16)	0.610	
GG vs. GA + AA (recessive)	Overall	13	53.6	0.011	0.83 (0.72–0.96)	0.013	0.89 (0.71–1.12)	0.321	0.332
	Population								
	Asian	11	54.8	0.015	0.82 (0.70–0.95)	0.011	0.89 (0.69–1.15)	0.377	
	Caucasian	2	66.0	0.086	0.92 (0.66–1.27)	0.611	0.44 (0.04–4.50)	0.490	
	Genotyping method								
	PCR–RFLP	10	63.4	0.003	0.82 (0.70–0.95)	0.010	0.87 (0.65–1.15)	0.326	
	ARMS-PCR	2	0.0	0.512	0.91 (0.60–1.39)	0.656	0.91 (0.60–1.39)	0.660	
	TaqMan-PCR	1	-	-	1.23 (0.49–3.07)	0.666	1.23 (0.49–3.07)	0.666	
GG vs.AA (homozygote model)	Overall	13	50.0	0.02	0.89 (0.75–1.06)	0.204	0.94 (0.71–1.25)	0.689	0.273
	Population								
	Asian	11	52.6	0.02	0.92 (0.75–1.13)	0.420	0.98 (0.71–1.36)	0.925	
	Caucasian	2	66.5	0.084	0.83 (0.59–1.16)	0.277	0.39 (0.04–4.14)	0.436	
	Genotyping method								
	PCR–RFLP	10	61.6	0.005	0.89 (0.73–1.08)	0.232	0.95 (0.66–1.37)	0.784	
	ARMS-PCR	2	0.0	0.455	0.91 (0.58–1.42)	0.669	0.91 (0.58–1.42)	0.672	
	TaqMan-PCR	1	-	-	0.93 (0.28–3.06)	0.903	0.93 (0.28–3.06)	0.903	
GG vs.GA (heterozygote model)	Overall	13	48.6	0.025	0.82 (0.71–0.96)	0.011	0.87 (0.69–1.10)	0.251	0.410
	Population								
	Asian	11	41.7	0.071	0.78 (0.66–0.92)	0.003	0.84 (0.67–1.07)	0.154	
	Caucasian	2	64.3	0.094	1.06 (0.75–1.50)	0.731	0.52 (0.05–5.11)	0.579	
	Genotyping method								
	PCR–RFLP	10	58.9	0.009	0.80 (0.68–0.94)	0.008	0.85 (0.51–1.41)	0.249	
	ARMS-PCR	2	0.0	0.637	0.90 (0.58–1.40)	0.632	0.90 (0.58–1.40)	0.634	
	TaqMan-PCR	1	-	-	1.38 (0.51–3.72)	0.519	1.38 (0.51–3.72)	0.519	
G vs. A (allele contrast model)	Overall	13	53.5	0.011	0.91 (0.84–0.98)	0.014	0.93 (0.82–1.06)	0.296	0.293
	Population								
	Asian	11	53.8	0.017	0.94 (0.85–1.03)	0.189	0.97 (0.83–1.14)	0.726	
	Caucasian	2	63.6	0.097	0.84 (0.74–0.97)	0.016	0.73 (0.45–1.19)	0.202	
	Genotyping method								
	PCR–RFLP	10	63.4	0.003	0.89 (0.78–1.15)	0.009	0.92 (0.78–1.09)	0.32	
	ARMS-PCR	2	0.0	0.500	0.97 (0.81–1.16)	0.748	0.97 (0.81–1.16)	0.749	
	TaqMan-PCR	1	-	-	1.00 (0.54–1.83)	0.992	1.00 (0.54–1.83)	0.992	

*Abbreviations:* *No.* Number, *RE* Random-effects, *FE* Fixed-effects, *PCR–RFLP* Polymerase chain reaction-restriction fragment length polymorphism, *ARMS-PCR* Amplification refractory mutation detection system-polymerase chain reaction

^−^ Not available

^*^ Publication bias test (Egger’s test)

**Fig. 2:**
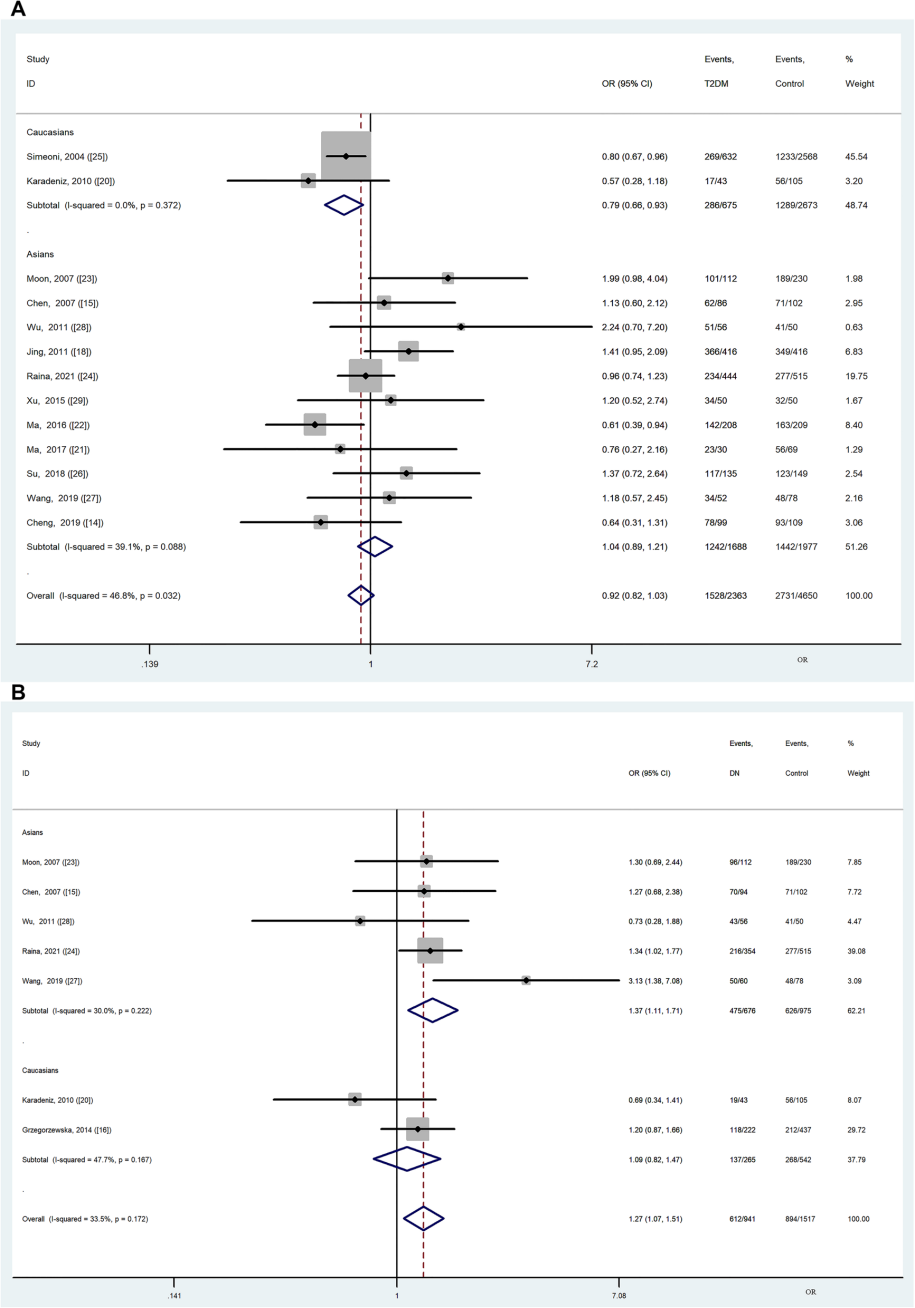

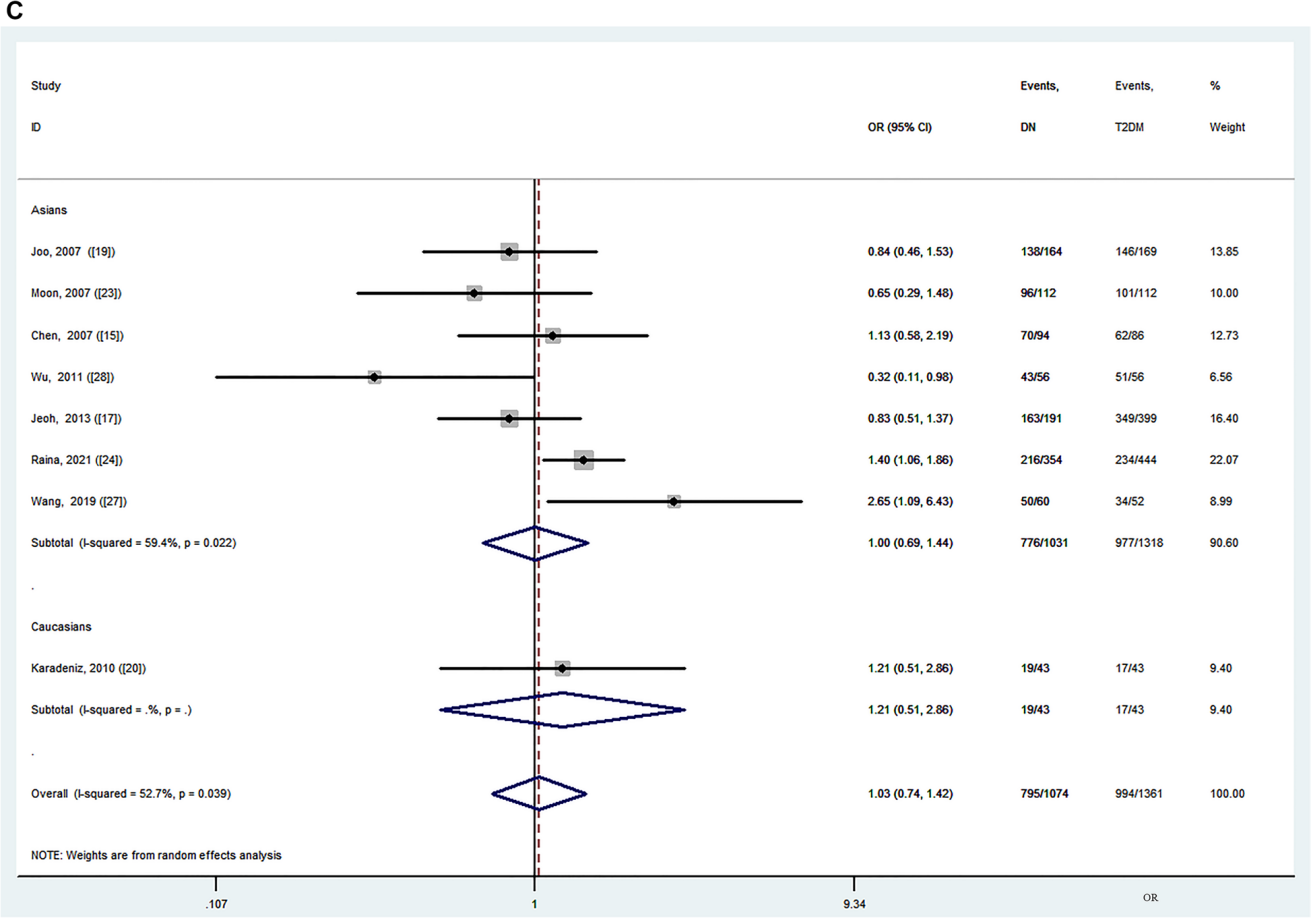
Forest plot for the association between the MCP-1 rs1024611 polymorphism and T2DM or DN risk with the dominant model (GG + GA vs. AA). **A** T2DM vs. healthy control; **B** DN vs. healthy control; **C** DN vs. T2DM

Ethnicity subgroup analyses were carried out in the Asian and Caucasian populations. The GG + GA genotype was associated with a lower risk of T2DM in Caucasians under the FE model (*OR* = 0.79, 95% CI: 0.66–0.93, *P* = 0.006) and RE model (*OR* = 0.79, 95% CI: 0.66–0.93, *P* = 0.006), without significant heterogeneity (*P*_*Q*_ = 0.372, *I*^*2*^ = 0.0%) (Fig. 2A; Table 2). The G allele was associated with a lower risk of T2DM in Caucasians under the FE model (*OR* = 0.84, 95% CI: 0.74–0.97, *P* = 0.016), but no significant risk was found under the RE model (*OR* = 0.73, 95% CI: 0.45–1.19, *P* = 0.202), with significant heterogeneity (*P*_*Q*_ =0.097, *I*^*2*^ = 63.6%). Significant associations with T2DM were found for the heterozygote model in Asians under the FE model (GG vs. GA: *OR* = 0.78, 95% CI: 0.66–0.92, *P* = 0.003), but no significant risk was found under the RE model (*OR* = 0.84, 95% CI: 0.67–1.07, *P* = 0.154), with heterogeneity (*P*_*Q*_ = 0.071, *I*^*2*^ = 41.7%) (Table 2). Associations with T2DM were also observed under the dominant model (GG + GA vs. AA: *OR* = 0.83, 95% CI: 0.72–0.95, *P* = 0.008) in studies without sex- or age-adjustment compared to studies with adjustment (Figure S1, Table S3). In contrast, no significant risks were found for the other four genetic models accounting for study population, genotyping method, comorbid chronic disease, and age- and sex-adjustment (Table 2, Table S3).

Meta-regression revealed that the study population, genotyping method, comorbid chronic disease, and age and sex adjustment were not the causes of heterogeneity in the five genetic models. A forest plot of the dominant model (GG + GA vs. AA) is presented in Fig. 2A and Figure S1.

### Association between the MCP-1 rs1024611 polymorphism and DN risk

Seven studies, five involving Asian populations and two involving Caucasian populations, were used to assess the potential correlations between the MCP-1 rs1024611 polymorphism and DN risk. The GG + GA genotype was associated with DN risk in the overall population (*OR* = 1.27, 95% CI: 1.07–1.51, *P* = 0.007) under the FE model (Table 3). Significant heterogeneity was detected in the overall population in other four genetic models (Table 3). Furthermore, no significant risks were found in the overall population under the RE model in the four genetic models (Table 3).

**Table 3:** Meta-analysis of the association between the MCP-1 rs1024611polymorphism and DN risk (DN vs. healthy control)

Genetic variant	Study group	No. studies	Heterogeneity test		Association test (FE model)		Association test (RE model)		Publication bias
			*I*^*2*^	*P* value	OR (95% CI)	*P* value	OR (95% CI)	*P* value	*P* value*
GG + GA vs.AA (dominant)	Overall	7	33.5	0.172	1.27 (1.07–1.51)	0.007	1.25 (0.98–1.60)	0.072	0.770
	Population								
	Asian	5	30.0	0.222	1.37 (1.11–1.71)	0.004	1.38 (1.01–1.88)	0.041	
	Caucasian	2	47.7	0.167	1.09 (0.82–1.47)	0.545	1.00 (0.60–1.67)	0.986	
	Genotyping method								
	PCR–RFLP	5	0.0	0.539	1.12 (0.88–1.42)	0.354	1.12 (0.88–1.42)	0.355	
	ARMS-PCR	2	72.8	0.055	1.48 (1.14–1.91)	0.003	1.87 (0.83–4.19)	0.128	
GG vs. GA + AA (recessive)	Overall	7	60.6	0.019	1.17 (0.93–1.47)	0.178	1.19 (0.79–1.78)	0.400	0.934
	Population								
	Asian	5	66.1	0.019	1.24 (0.96–1.59)	0.100	1.26 (0.80–2.00)	0.324	
	Caucasian	2	71.5	0.061	0.92 (0.54–1.56)	0.752	0.45 (0.79–1.78)	0.556	
	Genotyping method								
	PCR–RFLP	5	25.1	0.254	0.88 (0.66–1.18)	0.407	0.92 (0.65–1.32)	0.669	
	ARMS-PCR	2	19.4	0.265	1.92 (1.31–2.81)	0.001	1.96 (1.25–3.07)	0.003	
GG vs.AA (homozygote model)	Overall	7	57.8	0.027	1.42 (1.09–1.86)	0.010	1.40 (0.88–2.23)	0.160	0.984
	Population								
	Asian	5	58.8	0.046	1.61 (1.18–2.19)	0.003	1.55 (0.92–2.63)	0.099	
	Caucasian	2	73.9	0.050	0.97 (0.56–1.68)	0.907	0.45 (0.03–7.30)	0.576	
	Genotyping method								
	PCR–RFLP	5	19.3	0.291	1.02 (0.72–1.45)	0.918	1.07 (0.70–1.63)	0.763	
	ARMS-PCR	2	64.3	0.094	2.28 (1.50–3.47)	< 0.001	2.70 (1.13–6.43)	0.025	
GG vs.GA (heterozygote model)	Overall	7	44.8	0.093	1.06 (0.83–1.50)	0.652	1.08 (0.76–1.54)	0.676	0.821
	Population								
	Asian	5	49.5	0.094	1.11 (0.85–1.45)	0.445	1.13 (0.75–1.68)	0.564	
	Caucasian	2	66.4	0.085	0.86 (0.49–1.50)	0.594	0.46 (0.04–5.16)	0.532	
	Genotyping method								
	PCR–RFLP	5	14.1	0.324	0.83 (0.61–1.13)	0.229	0.87 (0.61–1.22)	0.414	
	ARMS-PCR	2	0.0	0.563	1.62 (1.08–2.43)	0.019	1.62 (1.08–2.43)	0.019	
G vs. A (allele contrast model)	Overall	7	67.2	0.006	1.17 (1.04–1.33)	0.010	1.13 (0.89–1.43)	0.304	0.892
	Population								
	Asian	5	68.3	0.013	1.23 (1.07–1.42)	0.004	1.23 (0.92–1.64)	0.166	
	Caucasian	2	76.5	0.039	1.04 (0.83–1.33)	0.754	0.87 (0.46–1.67)	0.684	
	Genotyping method								
	PCR–RFLP	5	32.8	0.203	1.01 (0.86–1.19)	0.877	0.98 (0.80–1.21)	0.886	
	ARMS-PCR	2	80.2	0.025	1.43 (1.19–1.72)	< 0.001	1.70 (0.94–3.05)	0.078	

*Abbreviations: No. Number, RE* Random-effects, *FE* Fixed-effects, *PCR–RFLP* Polymerase chain reaction-restriction fragment length polymorphism, *ARMS-PCR* Amplification refractory mutation detection system-polymerase chain reaction

^*^ Publication bias test (Egger’s test)

Ethnicity-stratified analysis indicated that the GG + GA genotype was associated with DN risk in the Asian population under the FE model (*OR* = 1.37, 95% CI: 1.11–1.71, *P* = 0.004) ,without significant heterogeneity (*P*_*Q*_ = 0.222, *I*^*2*^ = 30.0%). No significant risks were found in the Caucasian population for any genetic model under either the FE or RE models (Fig. 2B; Table 3). Three genetic models showed that the rs1024611 polymorphism was correlated with DN when applying the amplification refractory mutation detection system-polymerase chain reaction (ARMS-PCR) method (GG vs. GA + AA: *OR* = 1.92, 95% CI: 1.31–2.81, *P* = 0.001, *I*^*2*^ = 19.4%; GG vs. AA: *OR* = 2.70, 95% CI: 1.13–6.43, *P* = 0.025, *I*^*2*^ = 64.3%; GG vs. GA: *OR* = 1.62, 95% CI: 1.08–2.43, *P* = 0.019, *I*^*2*^ = 0.0%) (Table 3, Figure S2). Associations were also observed for the dominant model in patients with DN in sex- and age-adjusted studies (GG + GA vs. AA: *OR* = 1.34, 95% CI: 1.04–1.72, *P* = 0.024, *I*^*2*^ = 0.0%) and in patients with DN with no comorbid chronic diseases (GG + GA vs. AA: *OR* = 1.30, 95% CI: 1.05–1.59, *P* = 0.014, *I*^*2*^ = 43.9%) (Table S4, Figure S2). Meta-regression revealed that the study population, genotyping method, comorbid chronic disease, and age- and sex-adjustments were not the causes of heterogeneity in the five genetic models. A forest plot of the dominant model is presented in Fig. 2B and Figure S2.

We compared the DN and T2DM groups, including eight studies (seven involving Asian populations and one involving Caucasian populations) with 1,074 patients with DN and 1,361 patients with T2DM. Significant heterogeneity was detected in the overall and Asian populations under five genetic models (Table 4). No significant risks were found in the Asian or Caucasian populations for any genetic models under the RE model (Table 4; Fig. 2C). Five genetic models showed that the rs1024611 polymorphism was correlated with DN compared to T2DM when applying the ARMS-PCR method (GG vs. GA + AA: *OR* = 1.49, 95% CI: 1.14–1.95, *P* = 0.004, *I*^*2*^ = 43.7%; GG + GA vs. AA: *OR* = 2.10, 95% CI: 1.38–3.19, *P* < 0.001, *I*^*2*^ = 0.0%; GG vs. AA: *OR* = 2.52, 95% CI:1.59–3.97, *P* < 0.001, *I*^*2*^ = 0.0%; GG vs. GA: *OR* = 1.80, 95% CI:1.16–2.79, *P* = 0.009, *I*^*2*^ = 0.0%; G vs. A: *OR* = 1.58, 95% CI:1.08–2.32, *P* = 0.020, *I*^*2*^ = 51.4%) (Table 4, Figure S3). 

**Table 4:** Meta-analysis of the association between the MCP-1 rs1024611polymorphism and DN risk (DN vs. T2DM)

Genetic variant	Study group	No. of studies	Heterogeneity test		Association test (FE model)		Association test (RE model)		Publication bias
			*I*^*2*^	*P* value	OR (95% CI)	*P* value	OR (95% CI)	*P* value	*P* value*
GG + GA vs.AA (dominant)	Overall	8	52.7	0.039	1.13 (0.93–1.37)	0.227	1.03 (0.74–1.42)	0.881	0.572
	Population								
	Asian	7	59.4	0.022	1.12 (0.92–1.37)	0.254	1.00 (0.69–1.44)	0.996	
	Caucasian	1	*-*	*-*	1.21 (0.51–2.86)	0.662	1.21 (0.51–2.86)	0.662	
	Genotyping method								
	PCR–RFLP	6	0.0	0.458	0.83 (0.63–1.10)	0.197	0.84 (0.63–1.11)	0.220	
	ARMS-PCR	2	43.7	0.182	1.49 (1.14–1.95)	0.004	1.67 (0.96–2.90)	0.069	
GG vs. GA + AA (recessive)^a^	Overall	6	73.9	0.002	1.08 (0.86–1.35)	0.532	1.07 (0.66–1.72)	0.786	0.875
	Population								
	Asian	6	73.9	0.002	1.08 (0.86–1.35)	0.532	1.07 (0.66–1.72)	0.786	
	Genotyping method								
	PCR–RFLP	4	34.7	0.204	0.79 (0.60–1.04)	0.098	0.78 (0.54–1.11)	0.167	
	ARMS-PCR	2	0.0	0.728	2.10 (1.38–3.19)	< 0.001	2.10 (1.38–3.19)	0.001	
GG vs.AA (homozygote model)^a^	Overall	6	77.5	< 0.001	1.24 (0.92–1.66)	0.161	1.06 (0.54–2.10)	0.867	0.646
	Population								
	Asian	6	77.5	< 0.001	1.24 (0.92–1.66)	0.161	1.06 (0.54–2.10)	0.867	
	Genotyping method								
	PCR–RFLP	4	42.8	0.155	0.70 (0.47–1.05)	0.087	0.67 (0.38–1.17)	0.158	
	ARMS-PCR	2	0.0	0.399	2.52 (1.59–3.97)	< 0.001	2.51 (1.59–3.97)	< 0.001	
GG vs.GA (heterozygote model)^a^	Overall	6	56.1	0.044	1.06 (0.83–1.36)	0.631	1.05 (0.71–1.55)	0.802	0.975
	Population								
	Asian	6	56.1	0.044	1.06 (0.83–1.36)	0.631	1.05 (0.71–1.55)	0.802	
	Genotyping method								
	PCR–RFLP	4	7.5	0.355	0.83 (0.62–1.12)	0.218	0.83 (0.60–1.13)	0.236	
	ARMS-PCR	2	0.0	0.963	1.80 (1.16–2.79)	0.009	1.80 (1.16–2.79)	0.009	
G vs. A (allele contrast model)^b^	Overall	7	74.9	0.001	1.10 (0.96–1.26)	0.159	1.03 (0.76–1.40)	0.835	0.660
	Population								
	Asian	6	79.1	< 0.001	1.10 (0.96–1.26)	0.173	1.02 (0.73–1.42)	0.905	
	Caucasian	1	*-*	-	1.15 (0.55–2.40)	0.708	1.15 (0.55–2.40)	0.708	
	Genotyping method								
	PCR–RFLP	5	39.0	0.161	0.85 (0.70–1.02)	0.086	0.84 (0.65–1.08)	0.179	
	ARMS-PCR	2	51.4	0.152	1.46 (1.20–1.77)	< 0.001	1.58 (1.08–2.32)	0.020	

*Abbreviations: No. Number, RE* Random-effects, *FE* Fixed-effects, *PCR–RFLP* Polymerase chain reaction-restriction fragment length polymorphism, *ARMS-PCR* Amplification refractory mutation detection system-polymerase chain reaction

− Not available

* Publication bias test (Egger’s test)

^a^ OR (95% CI) in two articles could not be calculated (Jeoh et al., 2013 and Karadeniz et al., 2010 [20])

^b^ OR (95% CI) in one article could not be calculated (Jeoh et al., 2013)

Meta-regression revealed that genotyping method was a major driver of heterogeneity in five genetic models (GG + GA vs. AA: *P* = 0.032; GG vs. GA + AA: *P* = 0.028; GG vs. AA: *P* = 0.035; GG vs. GA: *P* = 0.041; G vs. A: *P* = 0.041), and there was a significant reduction in heterogeneity in the subgroup analysis for genotyping method.

### Sensitivity analysis

We performed sensitivity analysis using the leave-one-out method. No single study changed the summarized ORs for all of the genetic models, indicating that our findings are reliable (Figures S4-S6). 

### Publication bias

**Fig. 3:**
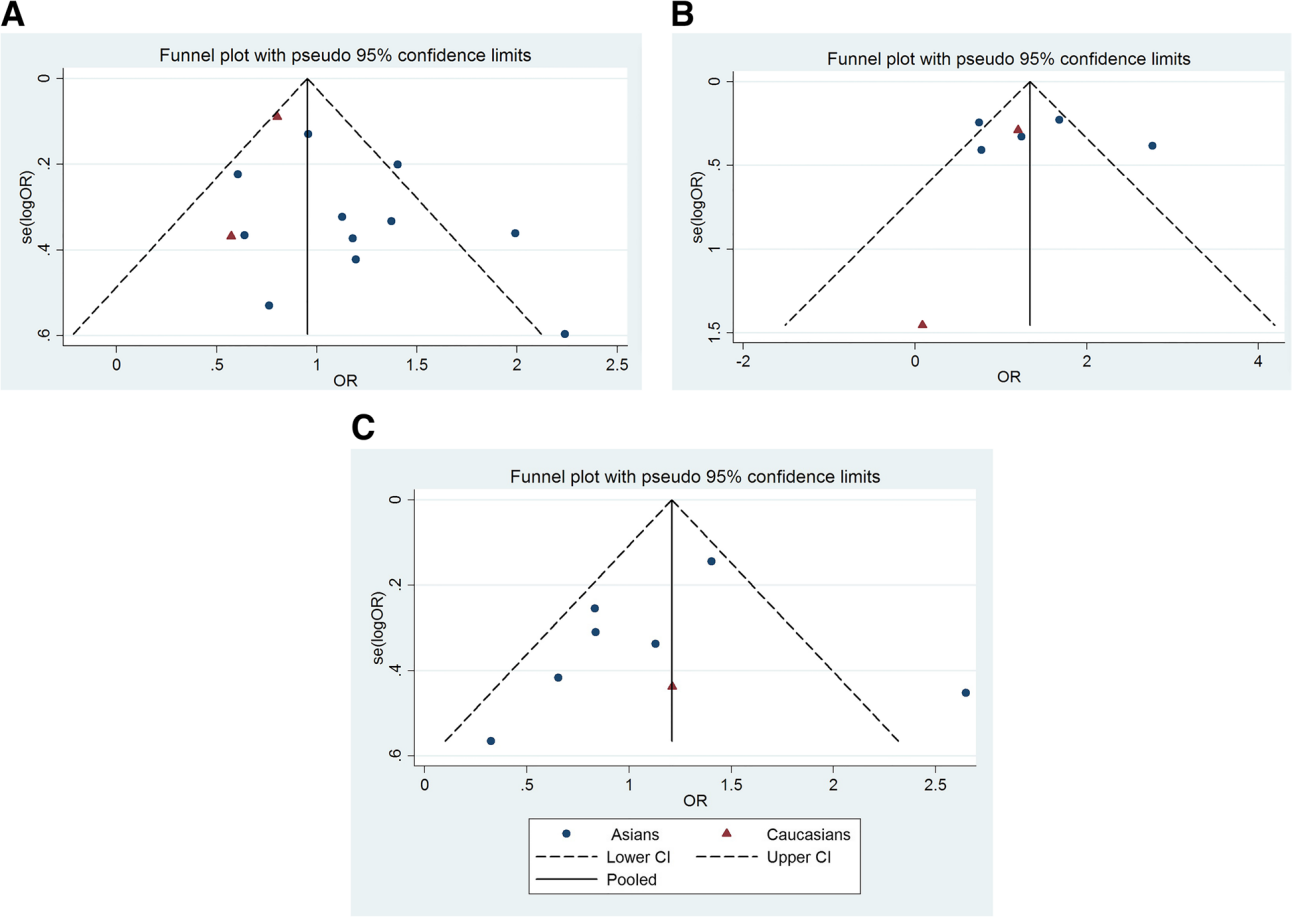
Funnel plots for the association between the MCP-1 rs1024611 polymorphism and T2DM or DN risk with the dominant model (GG + GA vs. AA). **A** T2DM vs. healthy control; **B** DN vs. healthy control; **C** DN vs. T2DM

A funnel plot showed no significant asymmetry under the dominant model (GG + AG vs. AA) (Fig. 3). *P* values obtained from Egger’s test are shown in Tables 2, 3 and 4, indicating that there was no publication bias in the four genetic models (T2DM vs. control: GG + GA vs. AA: *P* = 0.110; GG vs. GA + AA: *P* = 0.332; GG vs. AA: *P* = 0.273; GG vs. GA: *P* = 0.410; G vs. A: *P* = 0.293. DN vs. control: GG + GA vs. AA: *P* = 0.770; GG vs. GA + AA: *P* = 0.934; GG vs. AA: *P* = 0.984; GG vs. GA: *P* = 0.821; G vs. A: *P* = 0.892. DN vs. T2DM: GG + GA vs. AA: *P* = 0.572; GG vs. GA + AA: *P* = 0.875; GG vs. AA: *P* = 0.646; GG vs. GA: *P* = 0.975; G vs. A: *P* = 0.660).

## Discussion

Identification of the possible genetic origin of T2DM and DN could provide a theoretical basis for the early diagnosis or intervention of T2DM and DN. In this pooled analysis, we found that the GG + GA genotype was associated with a lower risk of T2DM in Caucasians, and that the GG + GA genotype was associated with DN risk in Asians. Subgroup analyses were conducted according to population, genotyping method, comorbid chronic disease, and age- and sex-adjustment. We conducted a detailed and comprehensive analysis under five genetic models and three comparative groups. No evidence of publication bias was observed under all genetic models. In addition, we adopted a sensitivity analysis, which did not affect the results of nonsensitivity analysis, indicating that our findings are trustworthy.

To the best of our knowledge, several meta-analyses have reported an association between the MCP-1 rs1024611 polymorphism and DN or T2DM risk [30–32]. For example, a 2011 report was the first to investigate this and indicated that the MCP-1 rs1024611 polymorphism was associated with a decreased risk of diabetes in Caucasians but not in Asians [30]; however, both T1DM and T2DM were included and data were not stratified by diabetic type. A 2015 meta-analysis found no association between the MCP-1 rs1024611 polymorphism and DN susceptibility [31]; however, the control group included patients with T2DM and healthy controls, which may influence the results obtained. Additionally, a 2014 meta-analysis comparing DN and T2DM indicated that the GA genotype might be a risk factor for the onset of nephropathy in T2DM among Asians [32]. Based on the above results, the role of the MCP-1 rs1024611 polymorphism in T2DM and DN susceptibility has been summarized and analyzed in the above meta-analyses with contradictory results. Different types of diabetes have heterogeneous pathophysiology [37, 38]. Differences in the selection of cases and controls could explain the observed contradictory results. In the present study, stringency in selecting the control group was maintained, and three groups were compared (T2DM cases and controls; DN cases and controls; T2DM cases and patients with DN). A recent study by Raina et al. (2021) comprising 350 T2DM cases (145 with end-stage renal disease and 205 without end-stage renal disease) and 221 controls in the Indian population have shown an association of the GG genotype and G allele with end-stage renal disease in T2DM cases [24]. An earlier study by Wang et al. (2019) in the Chinese population also associated carriage of the G-allele with DN [27]. However, the above published reviews did not include these two studies. Comprehensive evidence of the relationship between the MCP-1 rs1024611 polymorphism and T2DM and DN risk remains insufficient. A total of 16 studies were selected for the present meta-analysis. To the best of our knowledge, this study is one of the first to conduct a meta-analysis with the largest sample size. The comprehensiveness of the included articles is important for meta-analyses to draw more reliable conclusions.

Our meta-analysis was based on thirteen studies with 2,363 patients with T2DM and 4,650 healthy controls evaluating the association between the MCP-1 rs1024611 polymorphism and T2DM risk. Ethnicity-stratified analysis indicated that the GG + GA genotype might be a protective factor against T2DM susceptibility in Caucasians but not in Asians, which agrees with a previous meta-analysis [30]. The results of the of subgroup analysis (DN vs. healthy controls) differed from those of a previous meta-analysis [31]; we found that the GG + GA genotype was associated with DN risk in Asians, indicating that the MCP-1 rs1024611 G allele represents increased DN risk. This discrepancy might be due to increased research focusing on Asians. Only three of 16 studies involved Caucasians, suggesting that future studies should focus more on Caucasians.

Overall, our results indicate that the MCP-1 rs1024611 polymorphism has varying effects on T2DM and DN susceptibility in different ethnic groups. T2DM and DN are multietiology diseases that are genetically heterogeneous among different populations [39, 40]. Research from different countries shows that the frequency of the G allele for MCP-1 rs1024611 was 25.0% in Caucasians [25], 55.0% in Chinese people [18], and 61.9% in Koreans [23]. This phenomenon might also be explained by clinical heterogeneity. Differences in original research parameters (gender, age, disease severity, different stages of nephropathy, and method of diagnosing T2DM and DN, etc.) may have caused variations in the results [41]. Several studies found that the 2518G allele for MCP-1 rs1024611 was negatively correlated with plasma MCP-1 levels, insulin resistance, male sex, younger participants (age ≤ 50), and T2DM [18, 25]. However, stratified analysis of these factors has rarely been carried out.

This meta-analysis included eight studies with 1,074 patients with DN and 1,361 patients with T2DM and no significant risks were found in the Asian or Caucasian populations for any genetic models. This suggests that the MCP-1 rs1024611 polymorphism does not affect DN progression in T2DM. Because of the small sample size, this correlation needs to be verified by further multiethnic, large-sample-size studies. Meta-regression revealed that genotyping method was a major driver of heterogeneity in five genetic models when comparing the DN and T2DM groups. Additionally, there was a significant reduction in heterogeneity in subgroup analysis for genotyping method. However, study population, genotyping method, comorbid chronic disease, and age- and sex-adjustments were not causes of heterogeneity when comparing the DN or T2DM groups to the healthy group. Several factors significantly impacted heterogeneity (such as sex and disease stage), although the original articles did not provide more information on this. To the best of our knowledge, the present study is the first to identify a genotyping method as a source of heterogeneity in the relationship between the rs1024611 polymorphism and T2DM/DN susceptibility. To carry out a high-sensitivity rs1024611 SNP analysis, using ARMS-PCR is necessary.

Meta-analysis has greater statistical power than individual studies and can produce more reliable results [42, 43]. We used the NOS to evaluate the quality of the selected literature; sensitivity analysis yielded a similar result. A funnel plot and Egger’s test indicated no publication bias in any genetic model. These results suggest that our investigation was credible and robust. However, some limitations should also be acknowledged when interpreting the results. First, although this study had strict inclusion and exclusion criteria, significant heterogeneity existed under some genetic models in the overall and subgroup populations. After stratified analysis by genotyping method, there was a significant reduction in heterogeneity in subgroup analysis. Sex, age, and lifestyle are also related to T2DM and DN development, which may be sources of heterogeneity [44–46]. The original articles did not provide complete information on these confounding factors (such as age of onset, age matching, and sex ratio), and subgroup analysis of these factors was restricted. Second, most studies included in this meta-analysis were based on Asian populations, with few studies on Caucasian populations and none on African populations; hence, these data need to be verified in more Caucasian and African populations. Third, this meta-analysis only included Chinese and English articles, which may have led to language bias. Fourth, all the studies were case–control studies. A meta-analysis of cohort studies would give us a more valid result, and more cohort studies must be carried out in the future. Finally, the analysis of the correlation between the MCP-1 rs1024611 polymorphism and DN risk was based on small samples. Larger, well–designed case–control studies should be carried out in the future to elucidate the role of the MCP-1 rs1024611 polymorphism in DN susceptibility.

## Conclusions

In summary, the results of our meta-analysis demonstrate that the MCP-1 rs1024611 polymorphism is associated with T2DM susceptibility in Caucasians and with DN in Asians. Larger, well-designed cohort studies should be carried out in the future to verify this association.

### Supplementary Information

**Additional file 1: Figure S1.** Forest plot of T2DM risk with the dominant model (GG+GA vs. AA) (T2DM vs. healthy control) of the MCP-1 rs1024611 polymorphism. (A) for the overall populations and (B) genotyping method; (C) age- and sex- adjusted; (D) and comorbid chronic disease subgroups. **Figure S2.** Forest plot of DN risk with the dominant model (GG+GA vs. AA) (DN vs. healthy control) of the MCP-1 rs1024611 polymorphism. (A) for the overall populations and (B) genotyping method; (C) age- and sex- adjusted; (D) and comorbid chronic disease subgroups. **Figure S3.** Forest plot of DN risk with the dominant model (GG+GA vs. AA) (DN vs. T2DM) of the MCP-1 rs1024611 polymorphism. (A) for the overall populations and (B) genotyping method; (C) age- and sex- adjusted; (D) and comorbid chronic disease subgroups. **Figure S4.** Sensitivity analysis via deletion of each individual study (T2DM vs. healthy control). (A) for GG+GA vs. AA and (B) GG vs.GA + AA; (C) GG vs. AA; (D) GG vs. GA; (E) and G vs. A models. **Figure S5.** Sensitivity analysis via deletion of each individual study (DN vs. healthy control). (A) for GG+GA vs. AA and (B) GG vs.GA + AA; (C) GG vs. AA; (D) GG vs. GA; (E) and G vs. A models. **Figure S6.** Sensitivity analysis via deletion of each individual study (DN vs. T2DM). (A) for GG+GA vs. AA and (B) GG vs.GA + AA; (C) GG vs. AA; (D) GG vs. GA; (E) and G vs. A models. **Table S1.** The comprehensive search strategies for different databases. **Table S2.** Main characteristic of included observational studies evaluating the relationship between the MCP-1 rs1024611 polymorphism and DN/T2DM risk. **Table S3.** Meta-analysis of the association between the MCP-1 rs1024611polymorphism and T2DM risk (T2DM vs. healthy control). **Table S4.** Meta-analysis of the association between the MCP-1 rs1024611polymorphism and DN risk (DN vs. healthy control). **Table S5.** Meta-analysis of the association between the MCP-1 rs1024611polymorphism and DN risk (DN vs. T2DM).

## Data Availability

The datasets used and/or analyzed during the current study are available from the corresponding author upon reasonable request.

## Abbreviations

ARMS-PCR: Amplification refractory mutation detection system-polymerase chain reaction
No.: Number
T2DM: Type 2 Diabetes Mellitus
DN: Diabetic nephropathy
HWE: Hardy–Weinberg equilibrium
MCP-1: Monocyte chemoattractant protein-1
NOS: Newcastle–Ottawa scale
CNKI: Chinese National Knowledge Infrastructure
OR: Odds ratio
CI: Confidence interval
PCR-RFLP: Polymerase chain reaction-restriction fragment length polymorphism
RE: Random-effects
FE: Fixed-effects
SNP: Single-nucleotide polymorphism

## References

[1] Dunlay SM, Givertz MM, Aguilar D, Allen LA, Chan M, Desai AS (2019). Type 2 diabetes mellitus and heart failure: a scientific statement from the American Heart Association and the Heart Failure Society of America: this statement does not represent an update of the 2017 ACC/AHA/HFSA heart failure guideline update. Circulation.

[2] Sun H, Saeedi P, Karuranga S, Pinkepank M, Ogurtsova K, Duncan BB (2022). IDF diabetes atlas: global, regional and country-level diabetes prevalence estimates for 2021 and projections for 2045. Diabetes Res Clin Pract.

[3] Weerarathna T, Liyanage G, Herath M, Weerarathna M, Amarasinghe I (2018). Value of estimated glomerular filtration rate and albuminuria in predicting cardiovascular risk in patients with type 2 diabetes without cardiovascular disease. BioMed Res Int.

[4] Gurley SB, Ghosh S, Johnson SA, Azushima K, Sakban RB, George SE (2018). Inflammation and immunity pathways regulate genetic susceptibility to diabetic nephropathy. Diabetes.

[5] Heerspink HJ, De Zeeuw D (2016). Novel anti-inflammatory drugs for the treatment of diabetic kidney disease. Diabetologia.

[6] Zhou B, Li Q, Wang J, Chen P, Jiang S (2019). Ellagic acid attenuates streptozocin induced diabetic nephropathy via the regulation of oxidative stress and inflammatory signaling. Food Chem Toxicol.

[7] Ibi M, Horie S, Kyakumoto S, Chosa N, Yoshida M, Kamo M (2018). Cell-cell interactions between monocytes/macrophages and synoviocyte-like cells promote inflammatory cell infiltration mediated by augmentation of MCP-1 production in temporomandibular joint. Biosci Rep.

[8] França CN, Izar MCO, Hortêncio MNS, do Amaral JB, Ferreira CES, Tuleta ID (2017). Monocyte subtypes and the CCR2 chemokine receptor in cardiovascular disease. Clin Sci (Lond).

[9] Umare VD, Pradhan VD, Rajadhyaksha AG, Ghosh K, Nadkarni AH (2017). A functional SNP MCP-1 (-2518A/G) predispose to renal disorder in Indian systemic lupus Erythematosus patients. Cytokine.

[10] Nakamura T, Sato E, Amaha M, Kawagoe Y, Maeda S, Inoue H (2012). Ezetimibe reduces urinary albumin excretion in hypercholesterolaemic type 2 diabetes patients with microalbuminuria. J Int Med Res.

[11] Wang XY, Chen HT, Na R, Jiang DK, Lin XL, Yang F (2020). Single-nucleotide polymorphisms based genetic risk score in the prediction of pancreatic cancer risk. World J Gastroenterol.

[12] Rovin BH, Lu L, Saxena R (1999). A novel polymorphism in the MCP-1 gene regulatory region that influences MCP-1 expression. Biochem Biophys Res Commun.

[13] Zakharyan R, Boyajyan A, Arakelyan A, Melkumova M, Mrazek F, Petrek M (2012). Monocyte chemoattractant protein-1 in schizophrenia: -2518A/G genetic variant and protein levels in Armenian population. Cytokine.

[14] Cheng Y, Xue C, Wu JY, Lin HY, Zhao JJ, Mao XJ (2019). Association of MCP-1 gene 2518A/G polymorphism with diabetic retinopathy in type 2 diabetes mellitus patients. Zhejiang Med J.

[15] Chen J, Liu RH (2007). Study on the correlation of the A2518G polymorphism of MCP-1 gene and diabetic nephropathy in type 2 diabetic subjects. Chin J Diabetes.

[16] Grzegorzewska AE, Pajzderski D, Sowińska A, Jagodziński PP (2014). Polymporphism of monocyte chemoattractant protein 1 (MCP1 -2518 A/G) and responsiveness to hepatitis B vaccination in hemodialysis patients. Pol Arch Med Wewn.

[17] Jeon HJ, Choi HJ, Park BH, Lee YH, Oh T (2013). Association of monocyte chemoattractant protein-1 (MCP-1) 2518A/G polymorphism with proliferative diabetic retinopathy in Korean type 2 diabetes. Yonsei Med J.

[18] Jing Y, Zhu D, Bi Y, Yang D, Hu Y, Shen S (2011). Monocyte chemoattractant protein 1-2518 A/G polymorphism and susceptibility to type 2 diabetes in a Chinese population. Clin Chim Acta.

[19] Joo KW, Hwang YH, Kim JH, Oh KH, Kim H, Shin HD (2007). MCP-1 and RANTES polymorphisms in Korean diabetic end-stage renal disease. J Korean Med Sci.

[20] Karadeniz M, Erdogan M, Cetinkalp S, Berdeli A, Eroglu Z, Ozgen AG (2010). Monocyte chemoattractant protein-1 (MCP-1) 2518G/A gene polymorphism in Turkish type 2 diabetes patients with nephropathy. Endocrine.

[21] Ma FR, Ji SR, Chen H, He LR, Ren X, Yue RH (2017). Association of the serum MCP-1 concentration and genetic polymorphism with diabetic peripheral neuropathy in type 2 diabetes mellitus. J Lanzhou Univ (Medical Sciences).

[22] Ma JB, Xu X, Ma GG, Sheng J, Huang QH, Shi YP (2016). Association of monocyte chemoattractant protein-1 gene polymorphism with risk of type 2 diabetes mellitus in Han population in Zhejiang Province. Zhejiang Med J.

[23] Moon JY, Jeong L, Lee S, Jeong K, Lee T, Ihm CG (2007). Association of polymorphisms in monocyte chemoattractant protein-1 promoter with diabetic kidney failure in Korean patients with type 2 diabetes mellitus. J Korean Med Sci.

[24] Raina P, Sikka R, Gupta H, Matharoo K, Bali SK, Singh V (2021). Association of eNOS and MCP-1 genetic variants with type 2 diabetes and diabetic nephropathy susceptibility: a case-control and meta-analysis study. Biochem Genet.

[25] Simeoni E, Hoffmann MM, Winkelmann BR, Ruiz J, Fleury S, Boehm BO (2004). Association between the A-2518G polymorphism in the monocyte chemoattractant protein-1 gene and insulin resistance and type 2 diabetes mellitus. Diabetologia.

[26] Su N, Zhao N, Wang G, Wang L, Zhang Y, Li R (2018). Association of MCP-1 rs1024611 polymorphism with diabetic foot ulcers. Medicine (Baltimore).

[27] Wang Y, Li Y, Guo DX (2019). Relationship between peripheral blood monocyte chemoattractant protein-1 levels and gene polymorphism and diabetic Nephropathy. Guangxi Med J.

[28] Wu YQ, Zhang M, Ren XJ, Yang H, Long G (2011). Relationship between polym orphism ofM CP-1 and kidney failure in elderly patients with type 2 diabetes. Int J Endocrinol Metab.

[29] Xu J, Liao YF, Zhou WP, Ming HL, Wang QH (2015). The MCP-1 gene A-2518G polymorphism confers an increased risk of vascular complications in type 2 diabetes mellitus patients. Genet Test Mol Biomarkers.

[30] Zhang Y, Zhang J, Tian C, Narenqimuge, Deng Y, Zhao Y (2011). The – 2518A/G polymorphism in the monocyte chemoattractant protein-1 (MCP-1) gene and diabetes risk: a meta-analysis. Diabetes Res Clin Pract.

[31] Su N, Li HY, Huang MF, Jiang ZP, Zhou TB (2015). Association of monocyte chemoattractant protein-1 2518G/A gene polymorphism with diabetic nephropathy risk. J Recept Signal Transduct Res.

[32] Mao S, Huang S (2014). Monocyte chemoattractant protein-1 -2518G/A gene polymorphism and the risk of nephropathy in type 2 diabetes mellitus among Asians: a meta-analysis. Ren Fail.

[33] Page MJ, McKenzie JE, Bossuyt PM, Boutron I, Hoffmann TC, Mulrow CD (2021). The PRISMA 2020 statement: an updated guideline for reporting systematic reviews. BMJ.

[34] Stang A (2010). Critical evaluation of the Newcastle-Ottawa scale for the assessment of the quality of nonrandomized studies in meta-analyses. Eur J Epidemiol.

[35] Higgins JP, Thompson SG (2002). Quantifying heterogeneity in a meta-analysis. Stat Med.

[36] DerSimonian R, Kacker R (2007). Random-effects model for meta-analysis of clinical trials: an update. Contemp Clin Trials.

[37] Galicia-Garcia U, Benito-Vicente A, Jebari S, Larrea-Sebal A, Siddiqi H, Uribe KB (2020). Pathophysiology of type 2 diabetes mellitus. Int J Mol Sci.

[38] Skyler JS, Bakris GL, Bonifacio E, Darsow T, Eckel RH, Groop L (2017). Differentiation of diabetes by pathophysiology, natural history, and prognosis. Diabetes.

[39] van Zuydam NR, Ahlqvist E, Sandholm N, Deshmukh H, Rayner NW, Abdalla M (2018). A genome-wide association study of diabetic Kidney Disease in subjects with type 2 Diabetes. Diabetes.

[40] Tong Y, Lin Y, Zhang Y, Yang J, Zhang Y, Liu H (2009). Association between TCF7L2 gene polymorphisms and susceptibility to type 2 diabetes mellitus: a large Human Genome Epidemiology (HuGE) review and meta-analysis. BMC Med Genet.

[41] Kakoly NS, Khomami MB, Joham AE, Cooray SD, Misso ML, Norman RJ (2018). Ethnicity, obesity and the prevalence of impaired glucose tolerance and type 2 diabetes in PCOS: a systematic review and meta-regression. Hum Reprod Update.

[42] Cohn LD, Becker BJ (2003). How meta-analysis increases statistical power. Psychol Methods.

[43] Dawson DV, Pihlstrom BL, Blanchette DR (2016). Understanding and evaluating meta-analysis. J Am Dent Assoc.

[44] Bellou V, Belbasis L, Tzoulaki I, Evangelou E (2018). Risk factors for type 2 diabetes mellitus: an exposure-wide umbrella review of meta-analyses. PLoS One.

[45] Gao X, Hou R, Li X, Qiu XH, Luo HH, Liu SL (2021). The association between leucine and diabetic nephropathy in different gender: a cross-sectional study in Chinese patients with type 2 diabetes. Front Endocrinol (Lausanne).

[46] Kautzky-Willer A, Harreiter J, Pacini G (2016). Sex and gender differences in risk, pathophysiology and complications of type 2 diabetes mellitus. Endocr Rev.

