# Supplementary material for: Association between the MCP-1 -2518 A > G (rs1024611) polymorphism and susceptibility to type 2 diabetes mellitus and diabetic nephropathy: a meta-analysis
Source: BMC Endocr Disord. 2023 Dec 4;23:267. doi: 10.1186/s12902-023-01514-z (PMC10694925; doi:10.1186/s12902-023-01514-z)
Supplement: Supplementary file 1 — Additional file 1: Figure S1. Forest plot of T2DM risk with the dominant model (GG+GA vs. AA) (T2DM vs. healthy control) of the MCP-1 rs1024611 polymorphism. (A) for the overall populations and (B) genotyping method; (C) age- and sex- adjusted; (D) and comorbid chronic disease subgroups. Figure S2. Forest plot of DN risk with the dominant model (GG+GA vs. AA) (DN vs. healthy control) of the MCP-1 rs1024611 polymorphism. (A) for the overall populations and (B) genotyping method; (C) age- and sex- adjusted; (D) and comorbid chronic disease subgroups. Figure S3. Forest plot of DN risk with the dominant model (GG+GA vs. AA) (DN vs. T2DM) of the MCP-1 rs1024611 polymorphism. (A) for the overall populations and (B) genotyping method; (C) age- and sex- adjusted; (D) and comorbid chronic disease subgroups. Figure S4. Sensitivity analysis via deletion of each individual study (T2DM vs. healthy control). (A) for GG+GA vs. AA and (B) GG vs.GA + AA; (C) GG vs. AA; (D) GG vs. GA; (E) and G vs. A models. Figure S5. Sensitivity analysis via deletion of each individual study (DN vs. healthy control). (A) for GG+GA vs. AA and (B) GG vs.GA + AA; (C) GG vs. AA; (D) GG vs. GA; (E) and G vs. A models. Figure S6. Sensitivity analysis via deletion of each individual study (DN vs. T2DM). (A) for GG+GA vs. AA and (B) GG vs.GA + AA; (C) GG vs. AA; (D) GG vs. GA; (E) and G vs. A models. Table S1. The comprehensive search strategies for different databases. Table S2. Main characteristic of included observational studies evaluating the relationship between the MCP-1 rs1024611 polymorphism and DN/T2DM risk. Table S3. Meta-analysis of the association between the MCP-1 rs1024611polymorphism and T2DM risk (T2DM vs. healthy control). Table S4. Meta-analysis of the association between the MCP-1 rs1024611polymorphism and DN risk (DN vs. healthy control). Table S5. Meta-analysis of the association between the MCP-1 rs1024611polymorphism and DN risk (DN vs. T2DM). [file 12902_2023_1514_MOESM1_ESM.zip › Supplementary Tables 20231106.docx]

**Table S1 The comprehensive search strategies for different databases**

| **Database** | **Formula** | **No. of Results** |
| --- | --- | --- |
| **PubMed** | ((Diabetes mellitus[Title/Abstract] OR DM[Title/Abstract] OR nephropathy[Title/Abstract] OR diabetes[Title/Abstract]) AND (MCP-1[Title/Abstract] OR rs1024611[Title/Abstract] OR monocyte chemoattractant protein-1[Title/Abstract] OR CCL2[Title/Abstract])) AND (polymorphism OR genotype OR mutation) | 139 |
| EMBASE | ('diabetes mellitus':ab,ti OR 'dm':ab,ti OR 'nephropathy':ab,ti OR 'diabetes':ab,ti) AND ('mcp-1':ab,ti OR 'rs1024611':ab,ti OR 'monocyte chemoattractant protein-1':ab,ti OR 'ccl2':ab,ti) AND ('polymorphism' OR 'genotype' OR 'mutation') | 183 |
| **Web of Science** | [Diabetes mellitus OR DM OR nephropathy OR diabetes AND MCP-1 OR rs1042711 OR monocyte chemoattractant protein-1 OR CCL2  AND polymorphism OR genotype OR mutation](https://www.webofscience.com/wos/woscc/summary/518bec26-3912-4d71-9a72-9a022e467a56-8b052dba/relevance/1) | 73 |
| CNKI | (Diabetes mellitus [Title/Abstract/Keyword] OR nephropathy [Title/Abstract/Keyword]) AND (MCP-1 [Title/Abstract/Keyword] OR CCL2 [Title/Abstract/Keyword]) AND (polymorphism [Title/Abstract/Keyword]) | 60 |
| **Cochrane** | Diabetes mellitus OR DM OR nephropathy OR diabetes in Title Abstract Keyword AND MCP-1 OR rs1024611 OR monocyte chemoattractant protein-1 OR CCL2 OR 2518 in Title Abstract Keyword AND polymorphism OR genotype OR mutation in Title Abstract Keyword | 7 |

Table S2 Main characteristic of included observational studies evaluating the relationship between the MCP-1 rs1024611 polymorphism and DN/T2DM risk

| No. | First author,  year | Study type | Mean age (DN/T2DM/Control) | Male:Female(DN/T2DM/Control) | DN  diagnostic | T2DM diagnostic | Confounders | Disease  duration (Y) (DN/T2DM) | Genotyping method | Diabetes type | Comorbid  chronic  disease |
| --- | --- | --- | --- | --- | --- | --- | --- | --- | --- | --- | --- |
| 1 | Simeoni, 2004 | Case-control | -70.2/48.9 | -;1806/762;  430/202 | - | ADA & WHO | Not adjusted | None | PCR-  RFLP | 2 | No |
| 2 | Jooh, 2007 | Case-control | 61/64/- | 97/67;  68/101;  - | UAER>300mg/24 h | WHO | Not adjusted | 20/18 | PCR-  RFLP | 2 | Diabetic retinopathy |
| 3 | Moon, 2007 | Case-control | 58.2/58.8/58.9 | 42/70;  33/79;  80/150 | None | None | Adjusted for age, sex, and hypertension | 15.6/16.9 | PCR-  RFLP | 2 | No |
| 4 | Chen J, 2007 | Case-control | 54/53/54 | 56/38;  52/34;  61/41 | Two consecutive UAER>20 μg/min  within 6 months | WHO | Not adjusted | >10 | PCR-  RFLP | 2 | No |
| 5 | Karadeniz, 2010 | Case-control | 53.38/54.27/53.26 | None | UAER was 30–300 mg/24h in at least  two with 3 months | ADA | Not adjusted | 8.87  /8.53 | PCR-  RFLP | 2 | No |
| 6 | Wu YQ, 2011 | Case-control | 65/65/64 | 33/23;29/27;  26/24 | None | WHO | Not adjusted | 15.9 /17.3 | PCR-  RFLP | 2 | No |
| 7 | Jing Y, 2011 | Case-control | -/51.6/ 50.0 | -;214/202;  214/202 | - | ADA | Adjusted for age, sex, BMI, smoking status, hypertension, family history of diabetes  and residence | 0 | PCR-  RFLP | 2 | No |
| 8 | Jeoh, 2013 | Case-control | >59 | None | None | WHO | Not adjusted | >12 | PCR-  RFLP | 2 | No |
| 9 | Grzegorzewska, 2014 | Case-control | None | None | - | None | Not adjusted | None | PCR-  RFLP | 2 | HBV infection in HD patients |
| 10 | Raina, 2021 | Case-control | 60/56/44 | 242/112;  265/219;  237/287 | UAER>300mg /24 h | ADA | Adjusted for age, sex,BMI, and WHR | 12.72/  8.32 | ARMS- PCR | 2 | No |
| 11 | Xu J, 2015 | Case-control | -/56.83/58.35 | -;36/14;27/23 | - | WHO | Not adjusted | None | PCR-  RFLP | 2 | No |
| 12 | Ma JB, 2016 | Case-control | -/65.8/63.9 | -;143/65;  143/66 | - | WHO | Not adjusted | None | PCR-  RFLP | 2 | No |
| 13 | Ma FR, 2017 | Case-control | -/53.53/54.96 | -;19/11; 36/33 | - | WHO | Not adjusted | None | TaqMan  PCR | 2 | No |
| 14 | Su Na, 2018 | Case-control | -/64.53/65.36 | -;76/59; 83/66 | - | ADA | Not adjusted | None | PCR-  RFLP | 2 | No |
| 15 | Wang Y, 2019 | Case-control | 68.3/65.2/68.1 | 31/29;25/27;  42/36 | UAER>300 mg/24h | WHO | Not adjusted | 7.9/4.6 | ARMS-  PCR | 2 | No |
| 16 | Cheng Y, 2019 | Case-control | -/60.42/57.84 | -;47/52; 50/59 | - | None | Not adjusted | -/9.81 | PCR-  RFLP | 2 | No |

Abbreviations: No., number; T2DM, type 2 diabetes mellitus; DN, diabetic nephropathy; PCR-RFLP, polymerase chain reaction-restriction fragment length polymorphism; UAER, urinary albumin excretion rate; ADA, American Diagnostic Association; ARMS-PCR, amplification refractory mutation detection system-polymerase chain reaction.

Table S3 Meta-analysis of the association between the MCP-1 rs1024611polymorphism and T2DM risk (T2DM vs. healthy control)

| Genetic variant | Study group | No. studies | Heterogeneity test | |  | Association test  (FE model) | |  | Association test  (RE model) | | Publication bias |
| --- | --- | --- | --- | --- | --- | --- | --- | --- | --- | --- | --- |
|  |  |  | *I^2^* | *P* value |  | OR (95% CI) | *P* value |  | OR (95% CI) | *P* value | *P* value*** |
| GG+GA vs.AA (dominant) | Age and sex adjustment | |  |  |  |  |  |  |  |  | 0.110 |
|  | Yes | 3 | 62.0 | 0.072 |  | 1.14 (0.93–1.39) | 0.218 |  | 1.27 (0.86–1.87) | 0.235 |  |
|  | No | 10 | 20.1 | 0.258 |  | 0.83 (0.72–0.95) | 0.008 |  | 0.86 (0.70–1.05) | 0.127 |  |
|  | Comorbid chronic disease | |  |  |  |  |  |  |  |  |  |
|  | No | 13 | 46.8 | 0.032 |  | 0.92 (0.82–1.03) | 0.134 |  | 0.98 (0.81–1.20) | 0.855 |  |
| GG vs. GA +AA (recessive) | Age and sex adjustment | |  |  |  |  |  |  |  |  | 0.332 |
|  | Yes | 3 | 41.4 | 0.082 |  | 0.88 (0.73–1.05) | 0.159 |  | 0.86 (0.51–1.45) | 0.561 |  |
|  | No | 10 | 79.1 | 0.008 |  | 0.77 (0.62–0.97) | 0.026 |  | 0.91 (0.70–1.19) | 0.486 |  |
|  | Comorbid chronic disease | |  |  |  |  |  |  |  |  |  |
|  | No | 13 | 53.6 | 0.011 |  | 0.83 (0.72–0.96) | 0.013 |  | 0.89 (0.71–1.12) | 0.321 |  |
| GG vs.AA (homozygote model) | Age and sex adjustment | |  |  |  |  |  |  |  |  | 0.273 |
|  | Yes | 3 | 59.3 | 0.086 |  | 1.03 (0.76–1.39) | 0.859 |  | 1.09 (0.66–1.80) | 0.733 |  |
|  | No | 10 | 50.9 | 0.032 |  | 0.83 (0.67–1.03) | 0.094 |  | 0.89 (0.62–1.28) | 0.524 |  |
|  | Comorbid chronic disease | |  |  |  |  |  |  |  |  |  |
|  | No | 13 | 50.0 | 0.02 |  | 0.89 (0.75–1.06) | 0.204 |  | 0.94 (0.71–1.25) | 0.689 |  |
| GG vs.GA (heterozygote model) | Age and sex adjustment | |  |  |  |  |  |  |  |  | 0.410 |
|  | Yes | 3 | 76.7 | 0.014 |  | 0.70 (0.56–0.89) | 0.003 |  | 0.79 (0.47–1.32) | 0.363 |  |
|  | No | 10 | 21.6 | 0.244 |  | 0.92 (0.75–1.12) | 0.391 |  | 0.93 (0.73–1.18) | 0.540 |  |
|  | Comorbid chronic disease | |  |  |  |  |  |  |  |  |  |
|  | No | 13 | 48.6 | 0.025 |  | 0.82 (0.71–0.96) | 0.011 |  | 0.87 (0.69–1.10) | 0.251 |  |
| G vs. A (allele contrast model) | Age and sex adjustment | |  |  |  |  |  |  |  |  | 0.293 |
|  | Yes | 3 | 66.1 | 0.052 |  | 0.97 (0.85–1.10) | 0.621 |  | 1.01 (0.80–1.27) | 0.920 |  |
|  | No | 10 | 50.8 | 0.032 |  | 0.87 (0.79–0.96) | 0.006 |  | 0.90 (0.76–1.07) | 0.218 |  |
|  | Comorbid chronic disease | |  |  |  |  |  |  |  |  |  |
|  | No | 13 | 53.5 | 0.011 |  | 0.91 (0.84–0.98) | 0.014 |  | 0.93 (0.82–1.06) | 0.296 |  |

Abbreviations: RE, random-effects; FE, fixed-effects;

*, Publication bias test (Egger’s test)

Table S4 Meta-analysis of the association between the MCP-1 rs1024611polymorphism and DN risk (DN vs. healthy control)

| Genetic variant | Study group | No. studies | Heterogeneity test | |  | Association test  (FE model) | |  | Association test  (RE model) | | Publication bias |
| --- | --- | --- | --- | --- | --- | --- | --- | --- | --- | --- | --- |
|  |  |  | *I^2^* | *P* value |  | OR (95% CI) | *P* value |  | OR (95% CI) | *P* value | *P* value*** |
| GG+GA vs.AA (dominant) | Age and sex adjustment |  |  |  |  |  |  |  |  |  | 0.770 |
|  | Yes | 2 | 0.0 | 0.926 |  | 1.34 (1.04–1.72) | 0.024 |  | 1.34 (1.04–1.72) | 0.024 |  |
|  | No | 5 | 53.7 | 0.071 |  | 1.21 (0.95–1.54) | 0.126 |  | 1.20 (0.79–1.82) | 0.400 |  |
|  | Comorbid chronic disease | |  |  |  |  |  |  |  |  |  |
|  | Yes | 1 | - | - |  | 1.20 (0.87–1.66) | 0.260 |  | 1.20 (0.87–1.66) | 0.260 |  |
|  | No | 6 | 43.9 | 0.113 |  | 1.30 (1.05–1.59) | 0.014 |  | 1.26 (0.90–1.75) | 0.176 |  |
| GG vs. GA+ AA (recessive) | Age and sex adjustment |  |  |  |  |  |  |  |  |  | 0.934 |
|  | Yes | 2 | 83.0 | 0.015 |  | 1.15 (0.83–1.58) | 0.406 |  | 1.13 (0.51–2.50) | 0.768 |  |
|  | No | 5 | 56.7 | 0.056 |  | 1.19 (0.86–1.65) | 0.283 |  | 1.22 (0.70–2.11) | 0.488 |  |
|  | Comorbid chronic disease | |  |  |  |  |  |  |  |  |  |
|  | Yes | 1 |  |  |  | 1.20 (0.93–1.47) | 0.528 |  | 1.20 (0.93–1.47) | 0.528 |  |
|  | No | 6 | 67.2 | 0.009 |  | 1.16 (0.91–1.49) | 0.232 |  | 1.17 (0.71–1.93) | 0.528 |  |
| GG vs.AA (homozygote model) | Age and sex adjustment |  |  |  |  |  |  |  |  |  | 0.984 |
|  | Yes | 2 | 49.9 | 0.158 |  | 1.56 (1.05–2.31) | 0.027 |  | 1.48 (0.82–2.65) | 0.190 |  |
|  | No | 5 | 67.1 | 0.016 |  | 1.31 (0.91–1.89) | 0.149 |  | 1.30 (0.61–2.78) | 0.498 |  |
|  | Comorbid chronic disease | |  |  |  |  |  |  |  |  |  |
|  | Yes | 1 | - | - |  | 1.30 (0.72–2.34) | 0.385 |  | 1.30 (0.72–2.34) | 0.385 |  |
|  | No | 6 | 64.3 | 0.016 |  | 1.45 (1.08–1.96) | 0.015 |  | 1.39 (0.77–2.51) | 0.276 |  |
| GG vs.GA (heterozygote model) | Age and sex adjustment |  |  |  |  |  |  |  |  |  | 0.821 |
|  | Yes | 2 | 81.3 | 0.021 |  | 1.04 (0.74–1.46) | 0.832 |  | 1.02 (0.46–2.25) | 0.970 |  |
|  | No | 5 | 26.1 | 0.248 |  | 1.08 (0.76–1.53) | 0.668 |  | 1.13 (0.73–1.75) | 0.580 |  |
|  | Comorbid chronic disease | |  |  |  |  |  |  |  |  |  |
|  | Yes | 1 | - | - |  | 1.09 (0.60–1.98) | 0.765 |  | 1.09 (0.60–1.98) | 0.765 |  |
|  | No | 6 | 54.0 | 0.054 |  | 1.05 (0.81–1.37) | 0.718 |  | 1.07 (0.69–1.66) | 0.771 |  |
| G vs. A (allele contrast model) | Age and sex adjustment |  |  |  |  |  |  |  |  |  | 0.892 |
|  | Yes | 2 | 65.2 | 0.090 |  | 1.19 (1.00–1.41) | 0.045 |  | 1.14 (0.82–1.57) | 0.434 |  |
|  | No | 5 | 74.0 | 0.004 |  | 1.16 (0.97–1.37) | 0.098 |  | 1.12 (0.77–1.63) | 0.551 |  |
|  | Comorbid chronic disease | |  |  |  |  |  |  |  |  |  |
|  | Yes | 1 | - | - |  | 1.16 (0.90–1.48) | 0.251 |  | 1.16 (0.90–1.48) | 0.251 |  |
|  | No | 6 | 72.6 | 0.003 |  | 1.18 (1.03–1.36) | 0.020 |  | 1.12 (0.83–1.51) | 0.470 |  |

Abbreviations: RE, random-effects; FE, fixed-effects

-, not available

*, Publication bias test (Egger’s test)

Table S5 Meta-analysis of the association between the MCP-1 rs1024611polymorphism and DN risk (DN vs. T2DM)

| Genetic variant | Study group | No. of studies | Heterogeneity test | |  | Association test  (FE model) | |  | Association test  (RE model) | | Publication bias |
| --- | --- | --- | --- | --- | --- | --- | --- | --- | --- | --- | --- |
|  |  |  | *I^2^* | *P* value |  | OR (95% CI) | *P* value |  | OR (95% CI) | *P* value | *P* value* |
| GG+GA vs.AA (dominant) | Age and sex adjustment | |  |  |  |  |  |  |  |  | 0.572 |
|  | Yes | 2 | 66.8 | 0.083 |  | 1.29 (0.99–1.69) | 0.060 |  | 1.06 (0.51–2.18) | 0.877 |  |
|  | No | 6 | 48.5 | 0.084 |  | 0.97 (0.73–1.28) | 0.825 |  | 0.99 (0.65–1.50) | 0.960 |  |
|  | Comorbid chronic disease | |  |  |  |  |  |  |  |  |  |
|  | Yes | 1 | - | - |  | 0.84 (0.46–1.53) | 0.564 |  | 0.84 (0.46–1.53) | 0.564 |  |
|  | No | 7 | 56.3 | 0.033 |  | 1.16 (0.95–1.43) | 0.142 |  | 1.05 (0.73–1.53) | 0.782 |  |
| GG vs. GA +AA (recessive)^#^ | Age and sex adjustment | |  |  |  |  |  |  |  |  | 0.875 |
|  | Yes | 2 | 91.9 | <0.001 |  | 1.13 (0.79–1.60) | 0.503 |  | 1.05 (0.29–3.79) | 0.938 |  |
|  | No | 4 | 55.2 | 0.082 |  | 1.04 (0.77–1.41) | 0.804 |  | 1.06 (0.65–1.73) | 0.807 |  |
|  | Comorbid chronic disease | |  |  |  |  |  |  |  |  |  |
|  | Yes | 1 | - | - |  | 0.98 (0.63–1.51) | 0.911 |  | 0.98 (0.63–1.51) | 0.911 |  |
|  | No | 5 | 78.8 | 0.001 |  | 1.12 (0.85–1.46) | 0.422 |  | 1.09 (0.59–2.03) | 0.781 |  |
| GG vs.AA (homozygote model)^#^ | Age and sex adjustment | |  |  |  |  |  |  |  |  | 0.646 |
|  | Yes | 2 | 89.2 | 0.002 |  | 1.51 (0.99–2.32) | 0.058 |  | 1.08 (0.23–5.07) | 0.920 |  |
|  | No | 4 | 73.6 | 0.010 |  | 1.03 (0.68–1.55) | 0.904 |  | 1.03 (0.43–2.45) | 0.953 |  |
|  | Comorbid chronic disease | |  |  |  |  |  |  |  |  |  |
|  | Yes | 1 |  |  |  | 0.85 (0.44–1.63) | 0.616 |  | 0.85 (0.44–1.63) | 0.616 |  |
|  | No | 5 | 80.5 | <0.001 |  | 1.36 (0.98–1.90) | 0.068 |  | 1.10 (0.47–2.57) | 0.821 |  |
|  |  |  |  |  |  |  |  |  |  |  |  |
| GG vs.GA (heterozygote model)^#^ | Age and sex adjustment | |  |  |  |  |  |  |  |  | 0.975 |
|  | Yes | 2 | 88.7 | 0.003 |  | 1.08 (0.74–1.56) | 0.694 |  | 1.01 (0.33–3.14) | 0.985 |  |
|  | No | 4 | 0.0 | 0.475 |  | 1.05 (0.76–1.45) | 0.769 |  | 1.05 (0.76–1.46) | 0.775 |  |
|  | Comorbid chronic disease | |  |  |  |  |  |  |  |  |  |
|  | Yes | 1 | - | - |  | 1.02 (0.64–1.63) | 0.929 |  | 1.02 (0.64–1.63) | 0.929 |  |
|  | No | 5 | 64.8 | 0.023 |  | 1.08 (0.81–1.44) | 0.610 |  | 1.06 (0.64–1.76) | 0.826 |  |
|  |  |  |  |  |  |  |  |  |  |  |  |
| G vs. A (allele contrast model)^&^ | Age and sex adjustment | |  |  |  |  |  |  |  |  | 0.660 |
|  | Yes | 2 | 90.5 | 0.001 |  | 1.17 (0.97–1.40) | 0.099 |  | 0.98 (0.48–1.99) | 0.951 |  |
|  | No | 5 | 68.2 | 0.014 |  | 1.03 (0.84–1.26) | 0.775 |  | 1.05 (0.72–1.54) | 0.790 |  |
|  | Comorbid chronic disease | |  |  |  |  |  |  |  |  |  |
|  | Yes | 1 | - | - |  | 0.94 (0.69–1.29) | 0.704 |  | 0.94 (0.69–1.29) | 0.704 |  |
|  | No | 6 | 78.0 | <0.001 |  | 1.14 (0.98–1.33) | 0.082 |  | 1.05 (0.73–1.52) | 0.794 |  |

Abbreviations: RE, random-effects; FE, fixed-effects

-, not available

*, Publication bias test (Egger’s test)

^#^: OR (95% CI) in two articles could not be calculated (Jeoh et al.,2013 and Karadeniz et al.,2010)

^&^: OR (95% CI) in one article could not be calculated (Jeoh et al., 2013)
