# Supplementary figures and images for: Association between the MCP-1 -2518 A > G (rs1024611) polymorphism and susceptibility to type 2 diabetes mellitus and diabetic nephropathy: a meta-analysis
Source: BMC Endocr Disord. 2023 Dec 4;23:267. doi: 10.1186/s12902-023-01514-z (PMC10694925; doi:10.1186/s12902-023-01514-z)

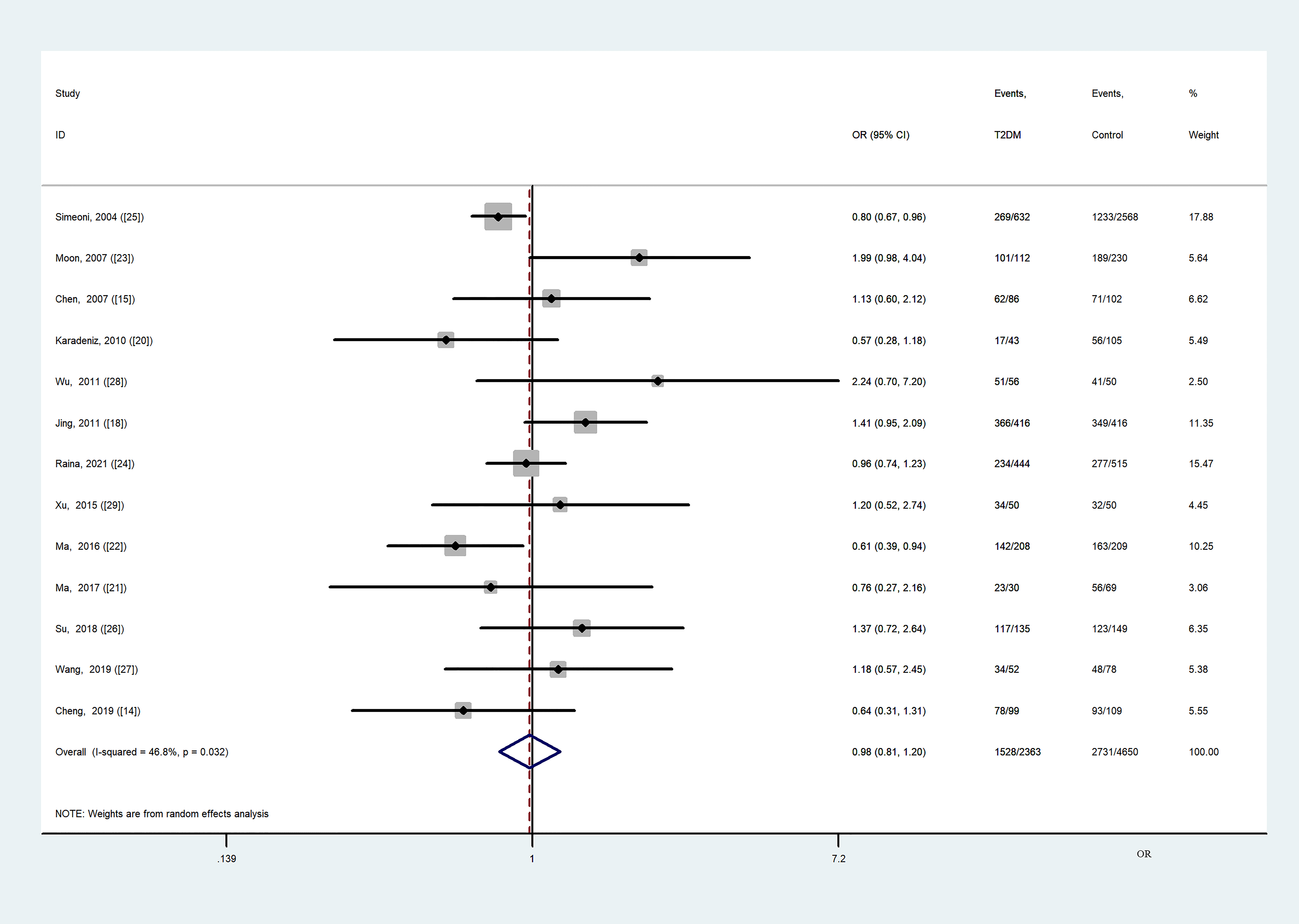

Supplement: Supplementary file 1 — Additional file 1: Figure S1. Forest plot of T2DM risk with the dominant model (GG+GA vs. AA) (T2DM vs. healthy control) of the MCP-1 rs1024611 polymorphism. (A) for the overall populations and (B) genotyping method; (C) age- and sex- adjusted; (D) and comorbid chronic disease subgroups. Figure S2. Forest plot of DN risk with the dominant model (GG+GA vs. AA) (DN vs. healthy control) of the MCP-1 rs1024611 polymorphism. (A) for the overall populations and (B) genotyping method; (C) age- and sex- adjusted; (D) and comorbid chronic disease subgroups. Figure S3. Forest plot of DN risk with the dominant model (GG+GA vs. AA) (DN vs. T2DM) of the MCP-1 rs1024611 polymorphism. (A) for the overall populations and (B) genotyping method; (C) age- and sex- adjusted; (D) and comorbid chronic disease subgroups. Figure S4. Sensitivity analysis via deletion of each individual study (T2DM vs. healthy control). (A) for GG+GA vs. AA and (B) GG vs.GA + AA; (C) GG vs. AA; (D) GG vs. GA; (E) and G vs. A models. Figure S5. Sensitivity analysis via deletion of each individual study (DN vs. healthy control). (A) for GG+GA vs. AA and (B) GG vs.GA + AA; (C) GG vs. AA; (D) GG vs. GA; (E) and G vs. A models. Figure S6. Sensitivity analysis via deletion of each individual study (DN vs. T2DM). (A) for GG+GA vs. AA and (B) GG vs.GA + AA; (C) GG vs. AA; (D) GG vs. GA; (E) and G vs. A models. Table S1. The comprehensive search strategies for different databases. Table S2. Main characteristic of included observational studies evaluating the relationship between the MCP-1 rs1024611 polymorphism and DN/T2DM risk. Table S3. Meta-analysis of the association between the MCP-1 rs1024611polymorphism and T2DM risk (T2DM vs. healthy control). Table S4. Meta-analysis of the association between the MCP-1 rs1024611polymorphism and DN risk (DN vs. healthy control). Table S5. Meta-analysis of the association between the MCP-1 rs1024611polymorphism and DN risk (DN vs. T2DM). [file 12902_2023_1514_MOESM1_ESM.zip › Figure S1/Figure S1A.jpg]

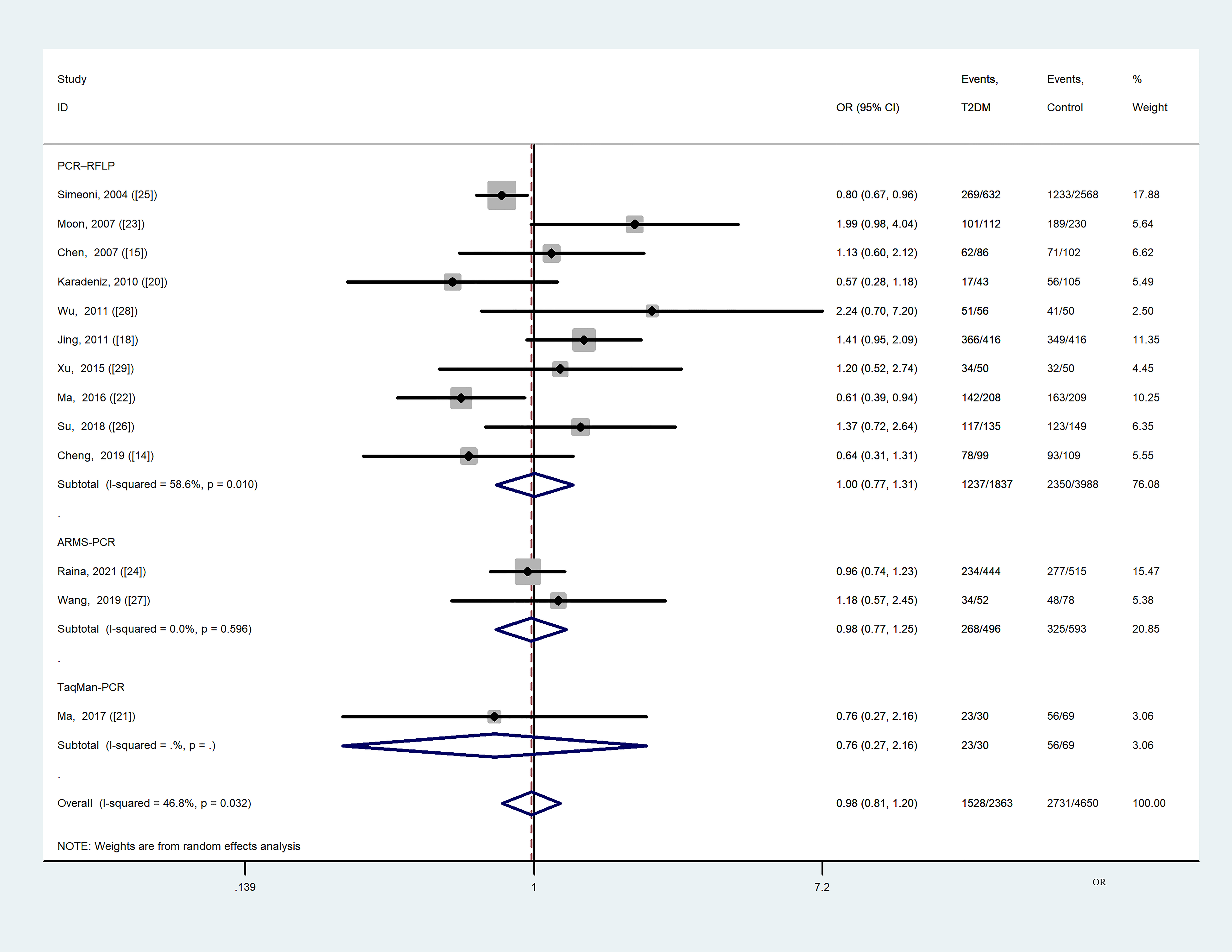

Supplement: Supplementary file 1 — Additional file 1: Figure S1. Forest plot of T2DM risk with the dominant model (GG+GA vs. AA) (T2DM vs. healthy control) of the MCP-1 rs1024611 polymorphism. (A) for the overall populations and (B) genotyping method; (C) age- and sex- adjusted; (D) and comorbid chronic disease subgroups. Figure S2. Forest plot of DN risk with the dominant model (GG+GA vs. AA) (DN vs. healthy control) of the MCP-1 rs1024611 polymorphism. (A) for the overall populations and (B) genotyping method; (C) age- and sex- adjusted; (D) and comorbid chronic disease subgroups. Figure S3. Forest plot of DN risk with the dominant model (GG+GA vs. AA) (DN vs. T2DM) of the MCP-1 rs1024611 polymorphism. (A) for the overall populations and (B) genotyping method; (C) age- and sex- adjusted; (D) and comorbid chronic disease subgroups. Figure S4. Sensitivity analysis via deletion of each individual study (T2DM vs. healthy control). (A) for GG+GA vs. AA and (B) GG vs.GA + AA; (C) GG vs. AA; (D) GG vs. GA; (E) and G vs. A models. Figure S5. Sensitivity analysis via deletion of each individual study (DN vs. healthy control). (A) for GG+GA vs. AA and (B) GG vs.GA + AA; (C) GG vs. AA; (D) GG vs. GA; (E) and G vs. A models. Figure S6. Sensitivity analysis via deletion of each individual study (DN vs. T2DM). (A) for GG+GA vs. AA and (B) GG vs.GA + AA; (C) GG vs. AA; (D) GG vs. GA; (E) and G vs. A models. Table S1. The comprehensive search strategies for different databases. Table S2. Main characteristic of included observational studies evaluating the relationship between the MCP-1 rs1024611 polymorphism and DN/T2DM risk. Table S3. Meta-analysis of the association between the MCP-1 rs1024611polymorphism and T2DM risk (T2DM vs. healthy control). Table S4. Meta-analysis of the association between the MCP-1 rs1024611polymorphism and DN risk (DN vs. healthy control). Table S5. Meta-analysis of the association between the MCP-1 rs1024611polymorphism and DN risk (DN vs. T2DM). [file 12902_2023_1514_MOESM1_ESM.zip › Figure S1/Figure S1B.jpg]

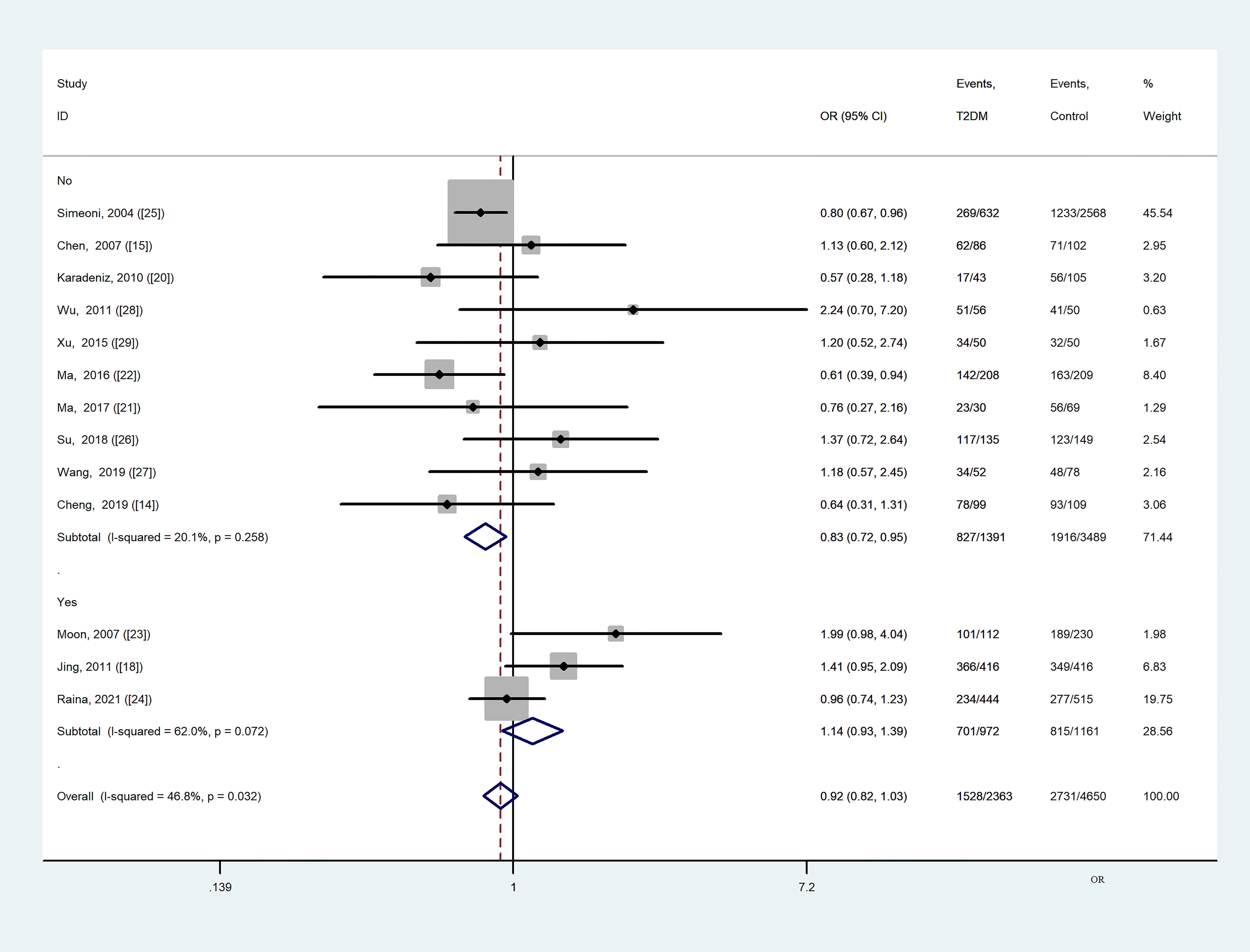

Supplement: Supplementary file 1 — Additional file 1: Figure S1. Forest plot of T2DM risk with the dominant model (GG+GA vs. AA) (T2DM vs. healthy control) of the MCP-1 rs1024611 polymorphism. (A) for the overall populations and (B) genotyping method; (C) age- and sex- adjusted; (D) and comorbid chronic disease subgroups. Figure S2. Forest plot of DN risk with the dominant model (GG+GA vs. AA) (DN vs. healthy control) of the MCP-1 rs1024611 polymorphism. (A) for the overall populations and (B) genotyping method; (C) age- and sex- adjusted; (D) and comorbid chronic disease subgroups. Figure S3. Forest plot of DN risk with the dominant model (GG+GA vs. AA) (DN vs. T2DM) of the MCP-1 rs1024611 polymorphism. (A) for the overall populations and (B) genotyping method; (C) age- and sex- adjusted; (D) and comorbid chronic disease subgroups. Figure S4. Sensitivity analysis via deletion of each individual study (T2DM vs. healthy control). (A) for GG+GA vs. AA and (B) GG vs.GA + AA; (C) GG vs. AA; (D) GG vs. GA; (E) and G vs. A models. Figure S5. Sensitivity analysis via deletion of each individual study (DN vs. healthy control). (A) for GG+GA vs. AA and (B) GG vs.GA + AA; (C) GG vs. AA; (D) GG vs. GA; (E) and G vs. A models. Figure S6. Sensitivity analysis via deletion of each individual study (DN vs. T2DM). (A) for GG+GA vs. AA and (B) GG vs.GA + AA; (C) GG vs. AA; (D) GG vs. GA; (E) and G vs. A models. Table S1. The comprehensive search strategies for different databases. Table S2. Main characteristic of included observational studies evaluating the relationship between the MCP-1 rs1024611 polymorphism and DN/T2DM risk. Table S3. Meta-analysis of the association between the MCP-1 rs1024611polymorphism and T2DM risk (T2DM vs. healthy control). Table S4. Meta-analysis of the association between the MCP-1 rs1024611polymorphism and DN risk (DN vs. healthy control). Table S5. Meta-analysis of the association between the MCP-1 rs1024611polymorphism and DN risk (DN vs. T2DM). [file 12902_2023_1514_MOESM1_ESM.zip › Figure S1/Figure S1C.jpg]

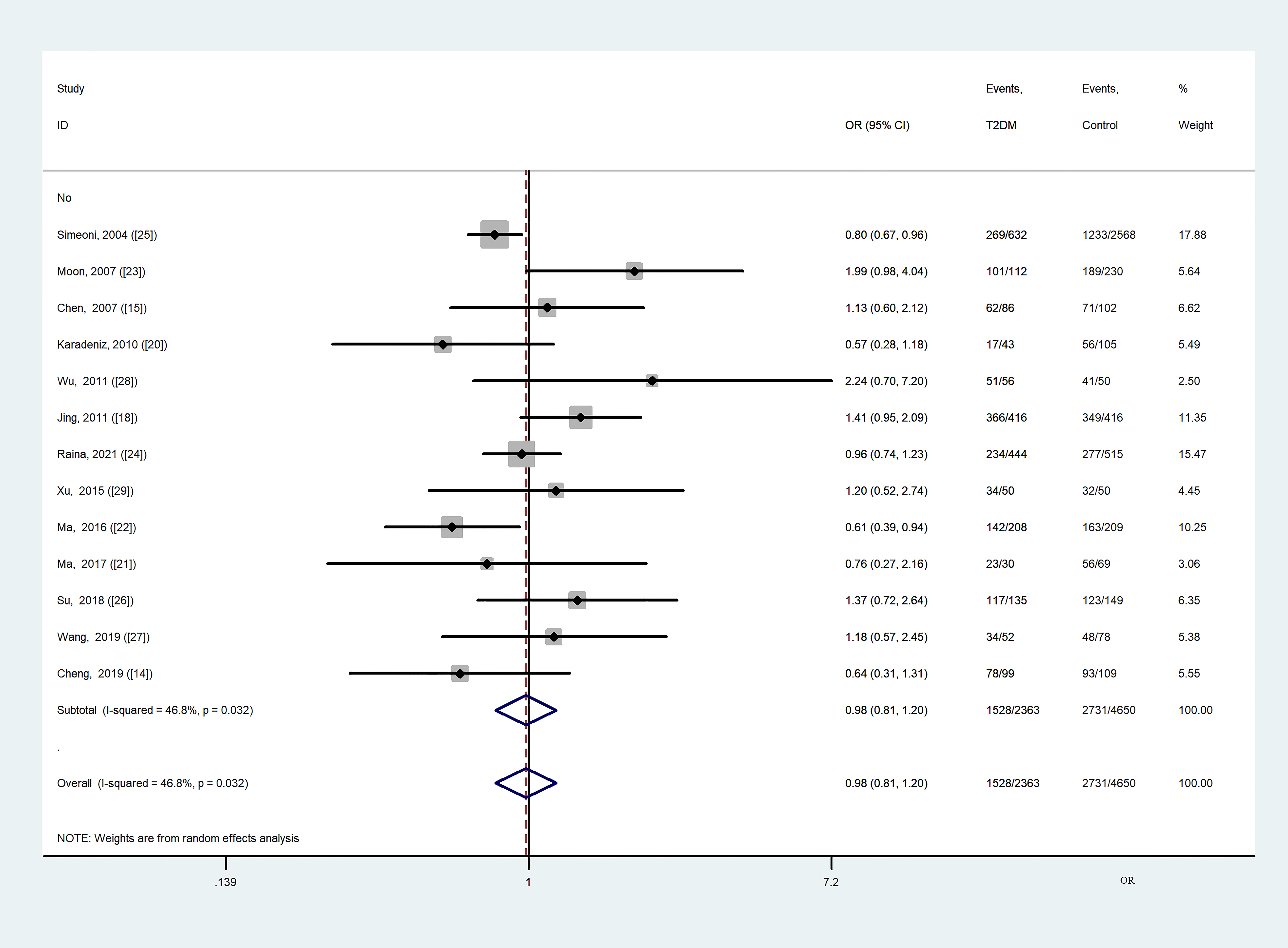

Supplement: Supplementary file 1 — Additional file 1: Figure S1. Forest plot of T2DM risk with the dominant model (GG+GA vs. AA) (T2DM vs. healthy control) of the MCP-1 rs1024611 polymorphism. (A) for the overall populations and (B) genotyping method; (C) age- and sex- adjusted; (D) and comorbid chronic disease subgroups. Figure S2. Forest plot of DN risk with the dominant model (GG+GA vs. AA) (DN vs. healthy control) of the MCP-1 rs1024611 polymorphism. (A) for the overall populations and (B) genotyping method; (C) age- and sex- adjusted; (D) and comorbid chronic disease subgroups. Figure S3. Forest plot of DN risk with the dominant model (GG+GA vs. AA) (DN vs. T2DM) of the MCP-1 rs1024611 polymorphism. (A) for the overall populations and (B) genotyping method; (C) age- and sex- adjusted; (D) and comorbid chronic disease subgroups. Figure S4. Sensitivity analysis via deletion of each individual study (T2DM vs. healthy control). (A) for GG+GA vs. AA and (B) GG vs.GA + AA; (C) GG vs. AA; (D) GG vs. GA; (E) and G vs. A models. Figure S5. Sensitivity analysis via deletion of each individual study (DN vs. healthy control). (A) for GG+GA vs. AA and (B) GG vs.GA + AA; (C) GG vs. AA; (D) GG vs. GA; (E) and G vs. A models. Figure S6. Sensitivity analysis via deletion of each individual study (DN vs. T2DM). (A) for GG+GA vs. AA and (B) GG vs.GA + AA; (C) GG vs. AA; (D) GG vs. GA; (E) and G vs. A models. Table S1. The comprehensive search strategies for different databases. Table S2. Main characteristic of included observational studies evaluating the relationship between the MCP-1 rs1024611 polymorphism and DN/T2DM risk. Table S3. Meta-analysis of the association between the MCP-1 rs1024611polymorphism and T2DM risk (T2DM vs. healthy control). Table S4. Meta-analysis of the association between the MCP-1 rs1024611polymorphism and DN risk (DN vs. healthy control). Table S5. Meta-analysis of the association between the MCP-1 rs1024611polymorphism and DN risk (DN vs. T2DM). [file 12902_2023_1514_MOESM1_ESM.zip › Figure S1/Figure S1D.jpg]

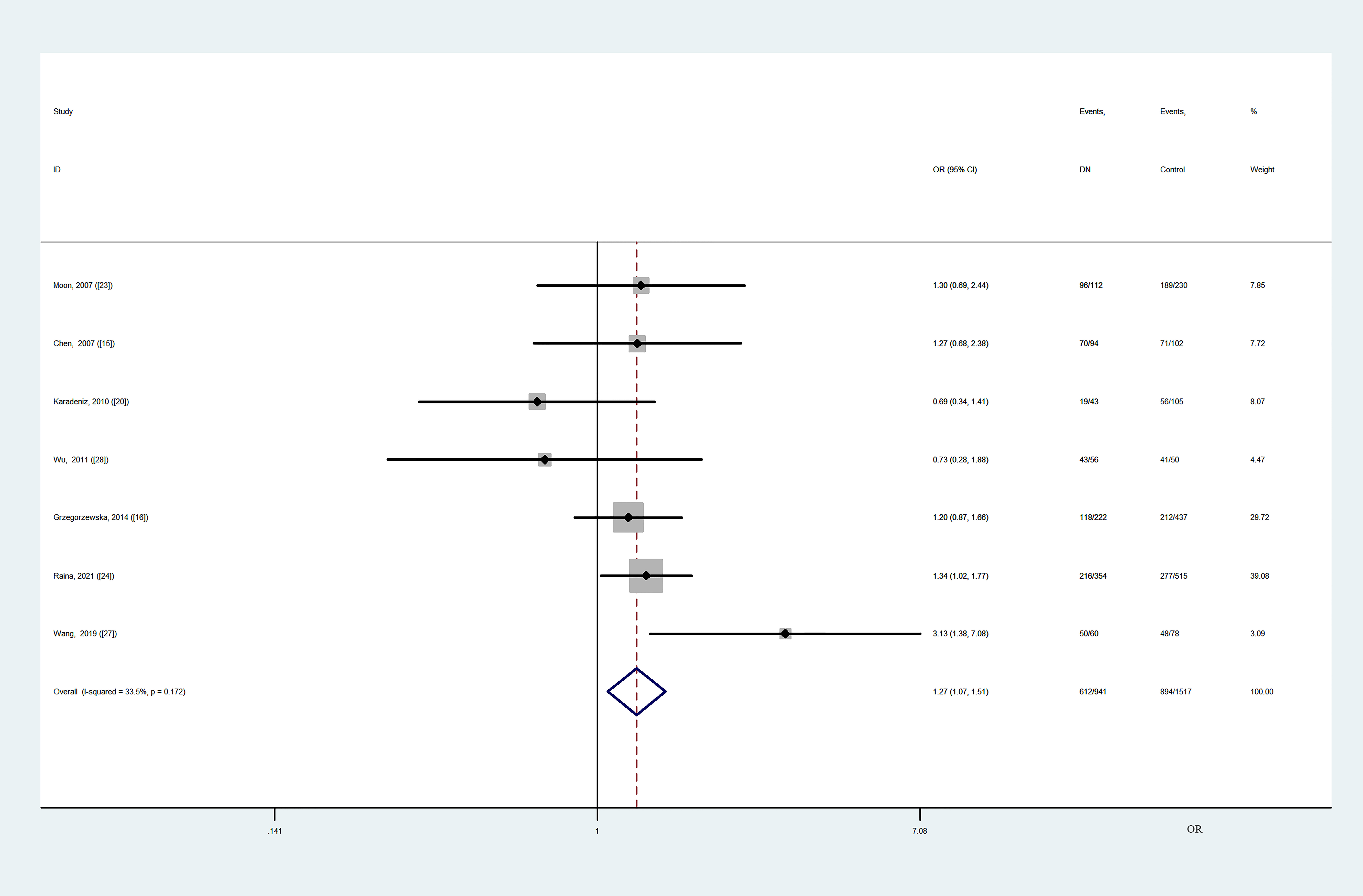

Supplement: Supplementary file 1 — Additional file 1: Figure S1. Forest plot of T2DM risk with the dominant model (GG+GA vs. AA) (T2DM vs. healthy control) of the MCP-1 rs1024611 polymorphism. (A) for the overall populations and (B) genotyping method; (C) age- and sex- adjusted; (D) and comorbid chronic disease subgroups. Figure S2. Forest plot of DN risk with the dominant model (GG+GA vs. AA) (DN vs. healthy control) of the MCP-1 rs1024611 polymorphism. (A) for the overall populations and (B) genotyping method; (C) age- and sex- adjusted; (D) and comorbid chronic disease subgroups. Figure S3. Forest plot of DN risk with the dominant model (GG+GA vs. AA) (DN vs. T2DM) of the MCP-1 rs1024611 polymorphism. (A) for the overall populations and (B) genotyping method; (C) age- and sex- adjusted; (D) and comorbid chronic disease subgroups. Figure S4. Sensitivity analysis via deletion of each individual study (T2DM vs. healthy control). (A) for GG+GA vs. AA and (B) GG vs.GA + AA; (C) GG vs. AA; (D) GG vs. GA; (E) and G vs. A models. Figure S5. Sensitivity analysis via deletion of each individual study (DN vs. healthy control). (A) for GG+GA vs. AA and (B) GG vs.GA + AA; (C) GG vs. AA; (D) GG vs. GA; (E) and G vs. A models. Figure S6. Sensitivity analysis via deletion of each individual study (DN vs. T2DM). (A) for GG+GA vs. AA and (B) GG vs.GA + AA; (C) GG vs. AA; (D) GG vs. GA; (E) and G vs. A models. Table S1. The comprehensive search strategies for different databases. Table S2. Main characteristic of included observational studies evaluating the relationship between the MCP-1 rs1024611 polymorphism and DN/T2DM risk. Table S3. Meta-analysis of the association between the MCP-1 rs1024611polymorphism and T2DM risk (T2DM vs. healthy control). Table S4. Meta-analysis of the association between the MCP-1 rs1024611polymorphism and DN risk (DN vs. healthy control). Table S5. Meta-analysis of the association between the MCP-1 rs1024611polymorphism and DN risk (DN vs. T2DM). [file 12902_2023_1514_MOESM1_ESM.zip › Figure S2/Figure S2A.jpg]

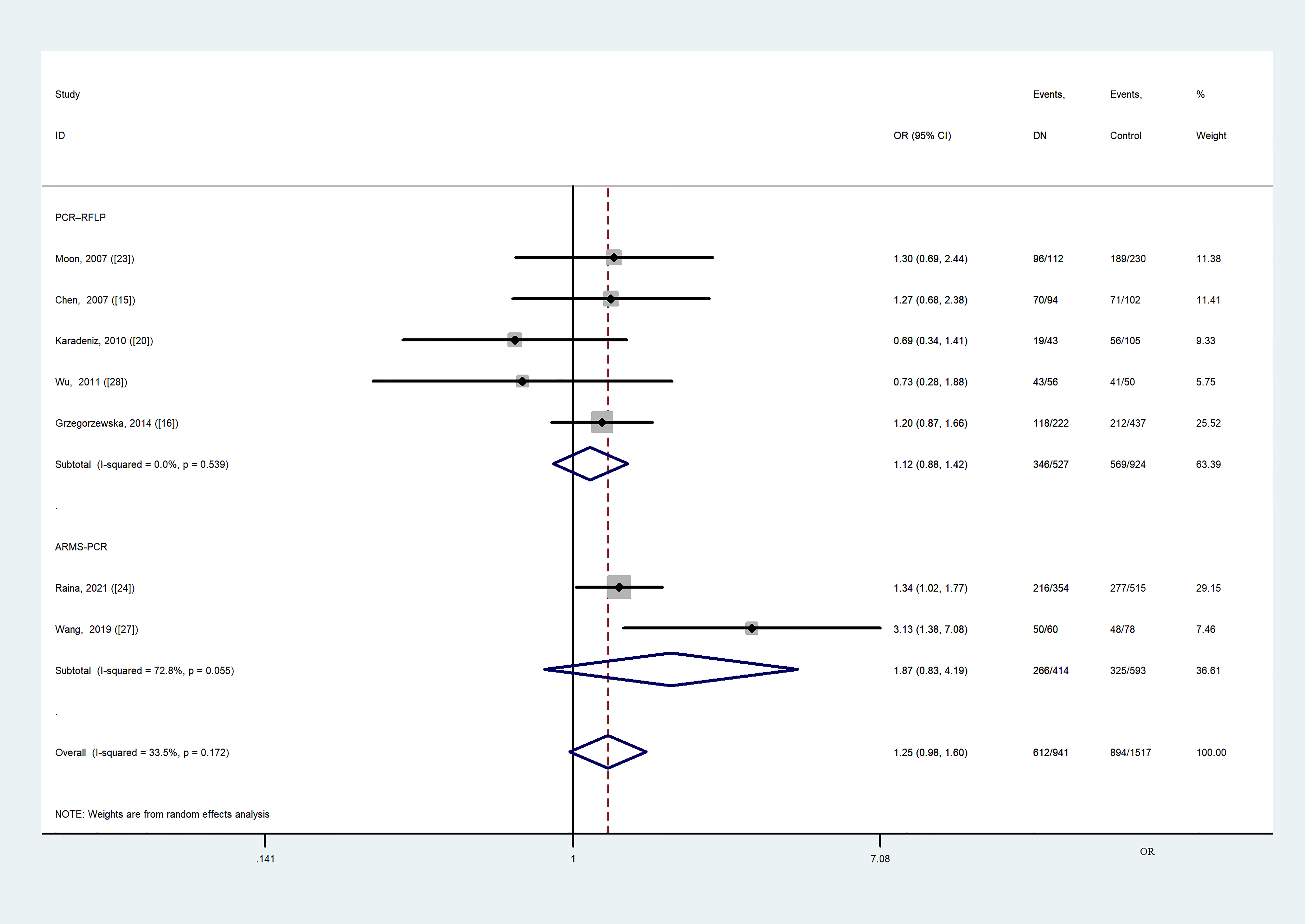

Supplement: Supplementary file 1 — Additional file 1: Figure S1. Forest plot of T2DM risk with the dominant model (GG+GA vs. AA) (T2DM vs. healthy control) of the MCP-1 rs1024611 polymorphism. (A) for the overall populations and (B) genotyping method; (C) age- and sex- adjusted; (D) and comorbid chronic disease subgroups. Figure S2. Forest plot of DN risk with the dominant model (GG+GA vs. AA) (DN vs. healthy control) of the MCP-1 rs1024611 polymorphism. (A) for the overall populations and (B) genotyping method; (C) age- and sex- adjusted; (D) and comorbid chronic disease subgroups. Figure S3. Forest plot of DN risk with the dominant model (GG+GA vs. AA) (DN vs. T2DM) of the MCP-1 rs1024611 polymorphism. (A) for the overall populations and (B) genotyping method; (C) age- and sex- adjusted; (D) and comorbid chronic disease subgroups. Figure S4. Sensitivity analysis via deletion of each individual study (T2DM vs. healthy control). (A) for GG+GA vs. AA and (B) GG vs.GA + AA; (C) GG vs. AA; (D) GG vs. GA; (E) and G vs. A models. Figure S5. Sensitivity analysis via deletion of each individual study (DN vs. healthy control). (A) for GG+GA vs. AA and (B) GG vs.GA + AA; (C) GG vs. AA; (D) GG vs. GA; (E) and G vs. A models. Figure S6. Sensitivity analysis via deletion of each individual study (DN vs. T2DM). (A) for GG+GA vs. AA and (B) GG vs.GA + AA; (C) GG vs. AA; (D) GG vs. GA; (E) and G vs. A models. Table S1. The comprehensive search strategies for different databases. Table S2. Main characteristic of included observational studies evaluating the relationship between the MCP-1 rs1024611 polymorphism and DN/T2DM risk. Table S3. Meta-analysis of the association between the MCP-1 rs1024611polymorphism and T2DM risk (T2DM vs. healthy control). Table S4. Meta-analysis of the association between the MCP-1 rs1024611polymorphism and DN risk (DN vs. healthy control). Table S5. Meta-analysis of the association between the MCP-1 rs1024611polymorphism and DN risk (DN vs. T2DM). [file 12902_2023_1514_MOESM1_ESM.zip › Figure S2/Figure S2B.jpg]

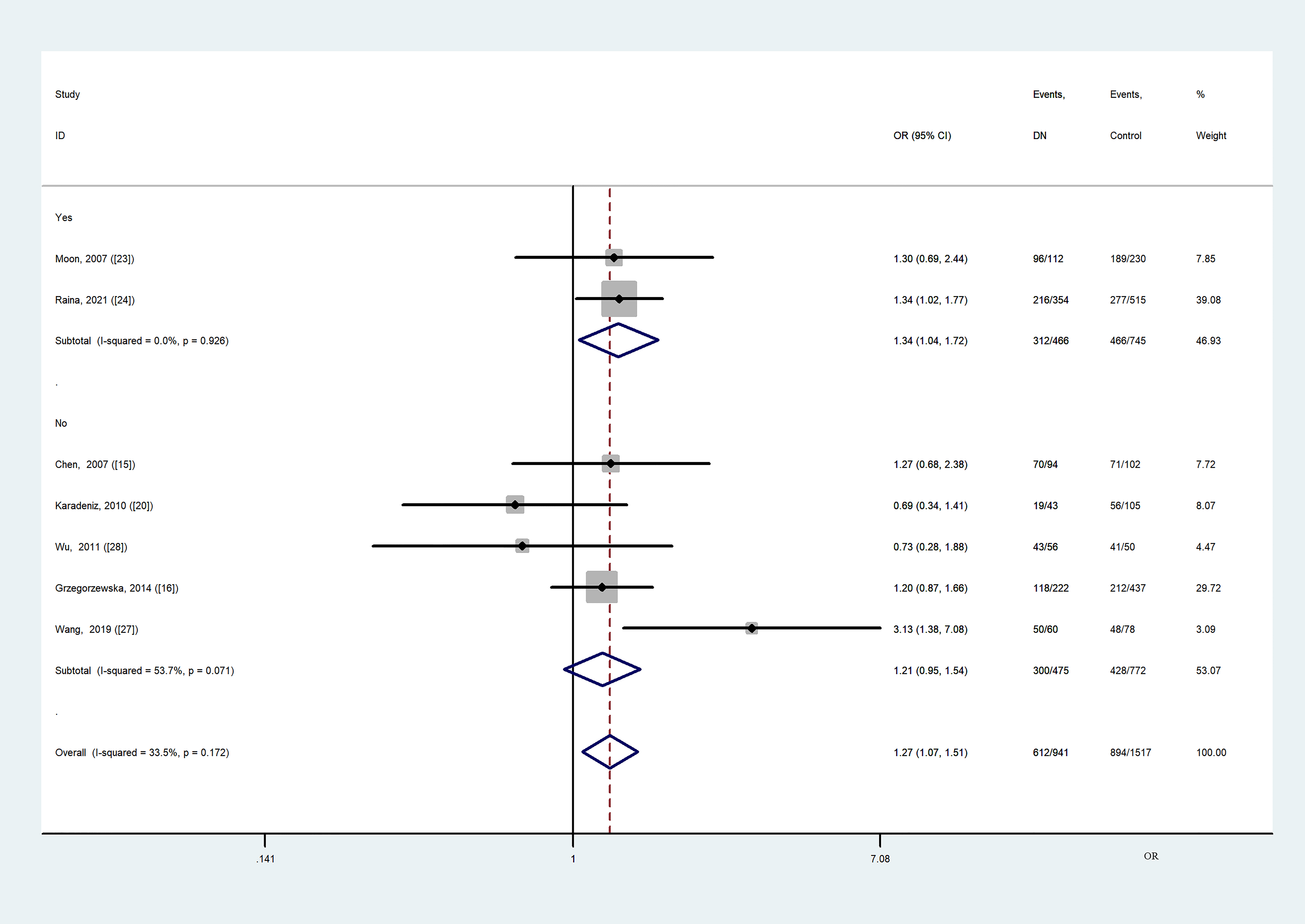

Supplement: Supplementary file 1 — Additional file 1: Figure S1. Forest plot of T2DM risk with the dominant model (GG+GA vs. AA) (T2DM vs. healthy control) of the MCP-1 rs1024611 polymorphism. (A) for the overall populations and (B) genotyping method; (C) age- and sex- adjusted; (D) and comorbid chronic disease subgroups. Figure S2. Forest plot of DN risk with the dominant model (GG+GA vs. AA) (DN vs. healthy control) of the MCP-1 rs1024611 polymorphism. (A) for the overall populations and (B) genotyping method; (C) age- and sex- adjusted; (D) and comorbid chronic disease subgroups. Figure S3. Forest plot of DN risk with the dominant model (GG+GA vs. AA) (DN vs. T2DM) of the MCP-1 rs1024611 polymorphism. (A) for the overall populations and (B) genotyping method; (C) age- and sex- adjusted; (D) and comorbid chronic disease subgroups. Figure S4. Sensitivity analysis via deletion of each individual study (T2DM vs. healthy control). (A) for GG+GA vs. AA and (B) GG vs.GA + AA; (C) GG vs. AA; (D) GG vs. GA; (E) and G vs. A models. Figure S5. Sensitivity analysis via deletion of each individual study (DN vs. healthy control). (A) for GG+GA vs. AA and (B) GG vs.GA + AA; (C) GG vs. AA; (D) GG vs. GA; (E) and G vs. A models. Figure S6. Sensitivity analysis via deletion of each individual study (DN vs. T2DM). (A) for GG+GA vs. AA and (B) GG vs.GA + AA; (C) GG vs. AA; (D) GG vs. GA; (E) and G vs. A models. Table S1. The comprehensive search strategies for different databases. Table S2. Main characteristic of included observational studies evaluating the relationship between the MCP-1 rs1024611 polymorphism and DN/T2DM risk. Table S3. Meta-analysis of the association between the MCP-1 rs1024611polymorphism and T2DM risk (T2DM vs. healthy control). Table S4. Meta-analysis of the association between the MCP-1 rs1024611polymorphism and DN risk (DN vs. healthy control). Table S5. Meta-analysis of the association between the MCP-1 rs1024611polymorphism and DN risk (DN vs. T2DM). [file 12902_2023_1514_MOESM1_ESM.zip › Figure S2/Figure S2C.jpg]

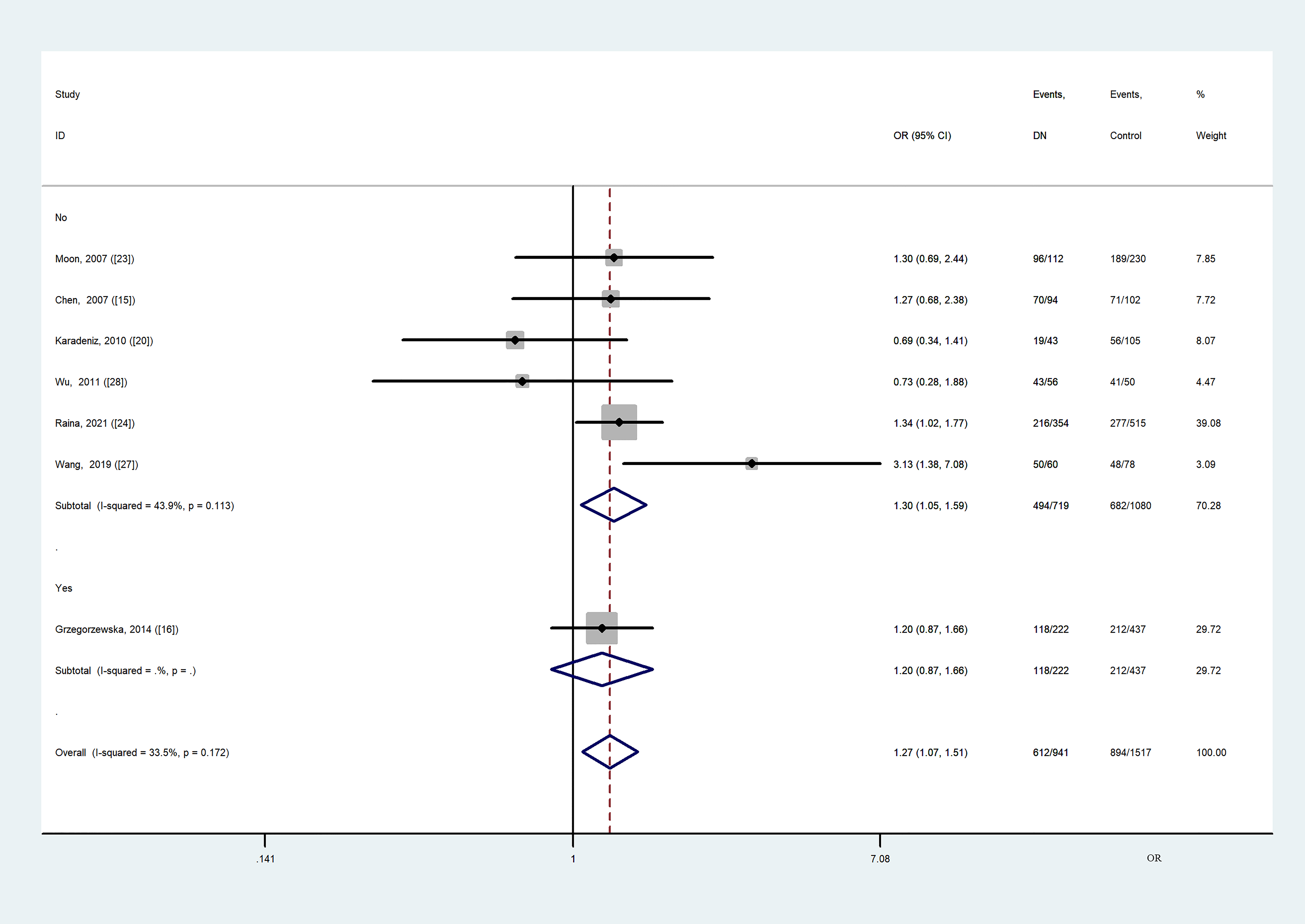

Supplement: Supplementary file 1 — Additional file 1: Figure S1. Forest plot of T2DM risk with the dominant model (GG+GA vs. AA) (T2DM vs. healthy control) of the MCP-1 rs1024611 polymorphism. (A) for the overall populations and (B) genotyping method; (C) age- and sex- adjusted; (D) and comorbid chronic disease subgroups. Figure S2. Forest plot of DN risk with the dominant model (GG+GA vs. AA) (DN vs. healthy control) of the MCP-1 rs1024611 polymorphism. (A) for the overall populations and (B) genotyping method; (C) age- and sex- adjusted; (D) and comorbid chronic disease subgroups. Figure S3. Forest plot of DN risk with the dominant model (GG+GA vs. AA) (DN vs. T2DM) of the MCP-1 rs1024611 polymorphism. (A) for the overall populations and (B) genotyping method; (C) age- and sex- adjusted; (D) and comorbid chronic disease subgroups. Figure S4. Sensitivity analysis via deletion of each individual study (T2DM vs. healthy control). (A) for GG+GA vs. AA and (B) GG vs.GA + AA; (C) GG vs. AA; (D) GG vs. GA; (E) and G vs. A models. Figure S5. Sensitivity analysis via deletion of each individual study (DN vs. healthy control). (A) for GG+GA vs. AA and (B) GG vs.GA + AA; (C) GG vs. AA; (D) GG vs. GA; (E) and G vs. A models. Figure S6. Sensitivity analysis via deletion of each individual study (DN vs. T2DM). (A) for GG+GA vs. AA and (B) GG vs.GA + AA; (C) GG vs. AA; (D) GG vs. GA; (E) and G vs. A models. Table S1. The comprehensive search strategies for different databases. Table S2. Main characteristic of included observational studies evaluating the relationship between the MCP-1 rs1024611 polymorphism and DN/T2DM risk. Table S3. Meta-analysis of the association between the MCP-1 rs1024611polymorphism and T2DM risk (T2DM vs. healthy control). Table S4. Meta-analysis of the association between the MCP-1 rs1024611polymorphism and DN risk (DN vs. healthy control). Table S5. Meta-analysis of the association between the MCP-1 rs1024611polymorphism and DN risk (DN vs. T2DM). [file 12902_2023_1514_MOESM1_ESM.zip › Figure S2/Figure S2D.jpg]

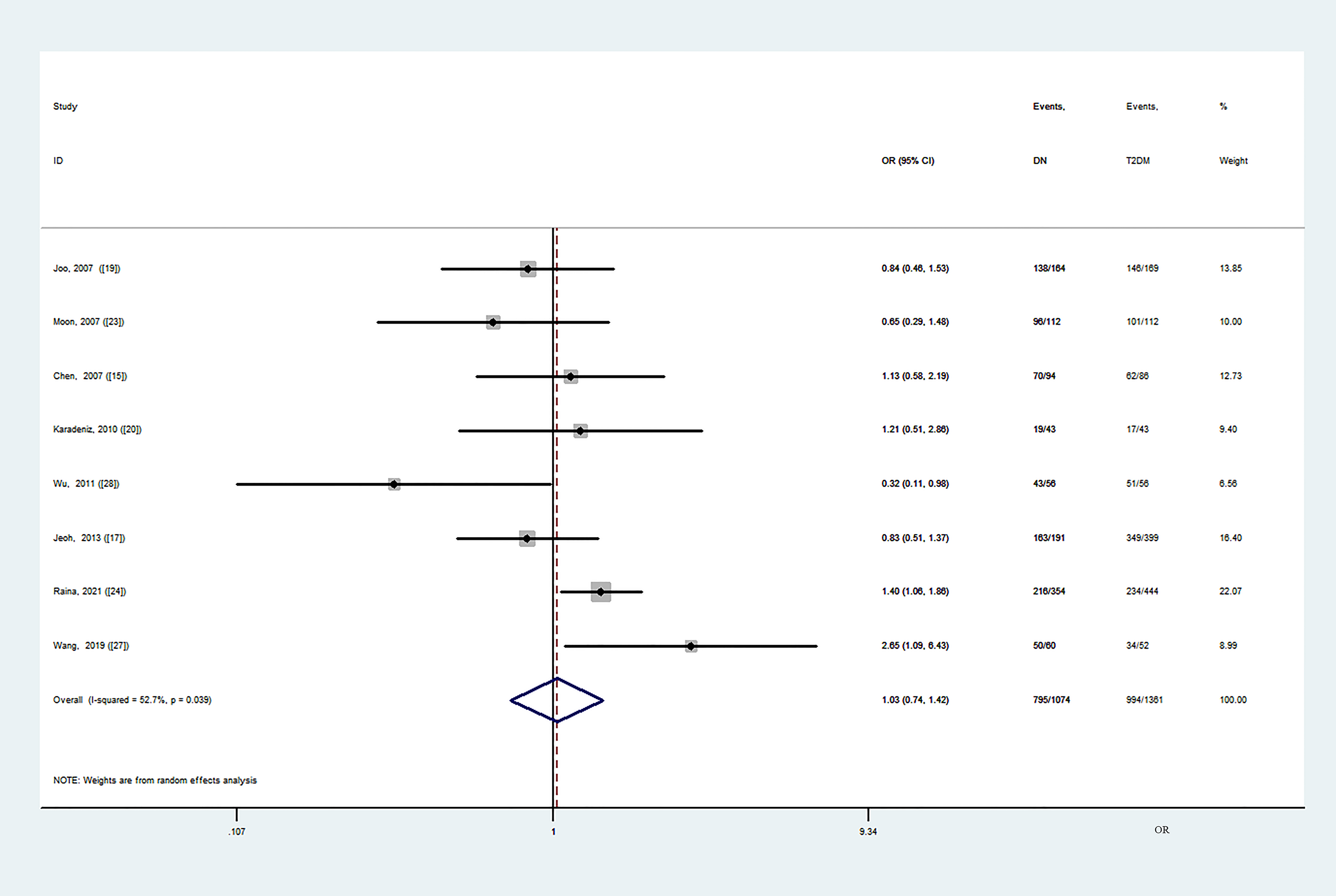

Supplement: Supplementary file 1 — Additional file 1: Figure S1. Forest plot of T2DM risk with the dominant model (GG+GA vs. AA) (T2DM vs. healthy control) of the MCP-1 rs1024611 polymorphism. (A) for the overall populations and (B) genotyping method; (C) age- and sex- adjusted; (D) and comorbid chronic disease subgroups. Figure S2. Forest plot of DN risk with the dominant model (GG+GA vs. AA) (DN vs. healthy control) of the MCP-1 rs1024611 polymorphism. (A) for the overall populations and (B) genotyping method; (C) age- and sex- adjusted; (D) and comorbid chronic disease subgroups. Figure S3. Forest plot of DN risk with the dominant model (GG+GA vs. AA) (DN vs. T2DM) of the MCP-1 rs1024611 polymorphism. (A) for the overall populations and (B) genotyping method; (C) age- and sex- adjusted; (D) and comorbid chronic disease subgroups. Figure S4. Sensitivity analysis via deletion of each individual study (T2DM vs. healthy control). (A) for GG+GA vs. AA and (B) GG vs.GA + AA; (C) GG vs. AA; (D) GG vs. GA; (E) and G vs. A models. Figure S5. Sensitivity analysis via deletion of each individual study (DN vs. healthy control). (A) for GG+GA vs. AA and (B) GG vs.GA + AA; (C) GG vs. AA; (D) GG vs. GA; (E) and G vs. A models. Figure S6. Sensitivity analysis via deletion of each individual study (DN vs. T2DM). (A) for GG+GA vs. AA and (B) GG vs.GA + AA; (C) GG vs. AA; (D) GG vs. GA; (E) and G vs. A models. Table S1. The comprehensive search strategies for different databases. Table S2. Main characteristic of included observational studies evaluating the relationship between the MCP-1 rs1024611 polymorphism and DN/T2DM risk. Table S3. Meta-analysis of the association between the MCP-1 rs1024611polymorphism and T2DM risk (T2DM vs. healthy control). Table S4. Meta-analysis of the association between the MCP-1 rs1024611polymorphism and DN risk (DN vs. healthy control). Table S5. Meta-analysis of the association between the MCP-1 rs1024611polymorphism and DN risk (DN vs. T2DM). [file 12902_2023_1514_MOESM1_ESM.zip › Figure S3/Figure S3A.jpg]

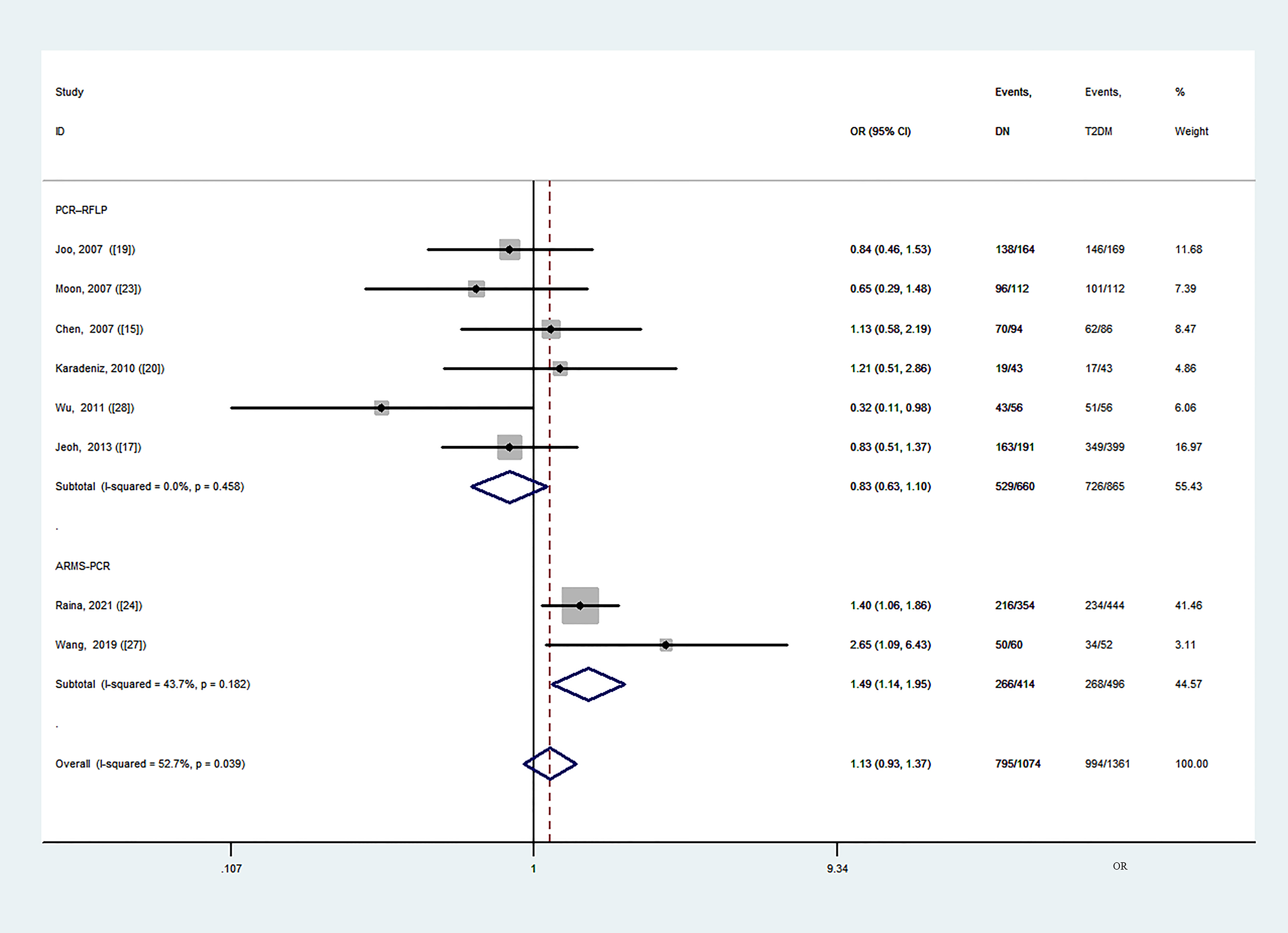

Supplement: Supplementary file 1 — Additional file 1: Figure S1. Forest plot of T2DM risk with the dominant model (GG+GA vs. AA) (T2DM vs. healthy control) of the MCP-1 rs1024611 polymorphism. (A) for the overall populations and (B) genotyping method; (C) age- and sex- adjusted; (D) and comorbid chronic disease subgroups. Figure S2. Forest plot of DN risk with the dominant model (GG+GA vs. AA) (DN vs. healthy control) of the MCP-1 rs1024611 polymorphism. (A) for the overall populations and (B) genotyping method; (C) age- and sex- adjusted; (D) and comorbid chronic disease subgroups. Figure S3. Forest plot of DN risk with the dominant model (GG+GA vs. AA) (DN vs. T2DM) of the MCP-1 rs1024611 polymorphism. (A) for the overall populations and (B) genotyping method; (C) age- and sex- adjusted; (D) and comorbid chronic disease subgroups. Figure S4. Sensitivity analysis via deletion of each individual study (T2DM vs. healthy control). (A) for GG+GA vs. AA and (B) GG vs.GA + AA; (C) GG vs. AA; (D) GG vs. GA; (E) and G vs. A models. Figure S5. Sensitivity analysis via deletion of each individual study (DN vs. healthy control). (A) for GG+GA vs. AA and (B) GG vs.GA + AA; (C) GG vs. AA; (D) GG vs. GA; (E) and G vs. A models. Figure S6. Sensitivity analysis via deletion of each individual study (DN vs. T2DM). (A) for GG+GA vs. AA and (B) GG vs.GA + AA; (C) GG vs. AA; (D) GG vs. GA; (E) and G vs. A models. Table S1. The comprehensive search strategies for different databases. Table S2. Main characteristic of included observational studies evaluating the relationship between the MCP-1 rs1024611 polymorphism and DN/T2DM risk. Table S3. Meta-analysis of the association between the MCP-1 rs1024611polymorphism and T2DM risk (T2DM vs. healthy control). Table S4. Meta-analysis of the association between the MCP-1 rs1024611polymorphism and DN risk (DN vs. healthy control). Table S5. Meta-analysis of the association between the MCP-1 rs1024611polymorphism and DN risk (DN vs. T2DM). [file 12902_2023_1514_MOESM1_ESM.zip › Figure S3/Figure S3B.jpg]

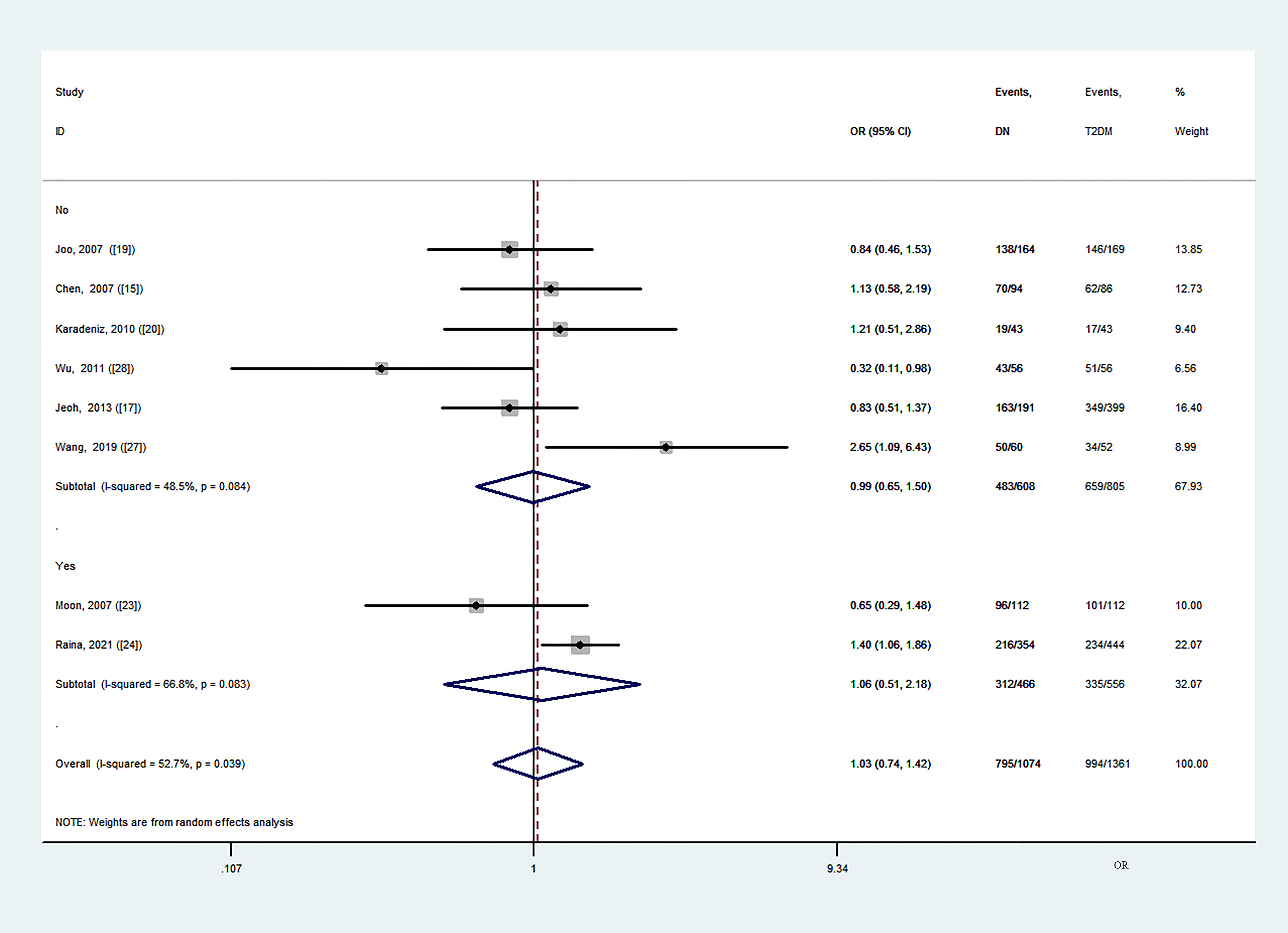

Supplement: Supplementary file 1 — Additional file 1: Figure S1. Forest plot of T2DM risk with the dominant model (GG+GA vs. AA) (T2DM vs. healthy control) of the MCP-1 rs1024611 polymorphism. (A) for the overall populations and (B) genotyping method; (C) age- and sex- adjusted; (D) and comorbid chronic disease subgroups. Figure S2. Forest plot of DN risk with the dominant model (GG+GA vs. AA) (DN vs. healthy control) of the MCP-1 rs1024611 polymorphism. (A) for the overall populations and (B) genotyping method; (C) age- and sex- adjusted; (D) and comorbid chronic disease subgroups. Figure S3. Forest plot of DN risk with the dominant model (GG+GA vs. AA) (DN vs. T2DM) of the MCP-1 rs1024611 polymorphism. (A) for the overall populations and (B) genotyping method; (C) age- and sex- adjusted; (D) and comorbid chronic disease subgroups. Figure S4. Sensitivity analysis via deletion of each individual study (T2DM vs. healthy control). (A) for GG+GA vs. AA and (B) GG vs.GA + AA; (C) GG vs. AA; (D) GG vs. GA; (E) and G vs. A models. Figure S5. Sensitivity analysis via deletion of each individual study (DN vs. healthy control). (A) for GG+GA vs. AA and (B) GG vs.GA + AA; (C) GG vs. AA; (D) GG vs. GA; (E) and G vs. A models. Figure S6. Sensitivity analysis via deletion of each individual study (DN vs. T2DM). (A) for GG+GA vs. AA and (B) GG vs.GA + AA; (C) GG vs. AA; (D) GG vs. GA; (E) and G vs. A models. Table S1. The comprehensive search strategies for different databases. Table S2. Main characteristic of included observational studies evaluating the relationship between the MCP-1 rs1024611 polymorphism and DN/T2DM risk. Table S3. Meta-analysis of the association between the MCP-1 rs1024611polymorphism and T2DM risk (T2DM vs. healthy control). Table S4. Meta-analysis of the association between the MCP-1 rs1024611polymorphism and DN risk (DN vs. healthy control). Table S5. Meta-analysis of the association between the MCP-1 rs1024611polymorphism and DN risk (DN vs. T2DM). [file 12902_2023_1514_MOESM1_ESM.zip › Figure S3/Figure S3C.jpg]

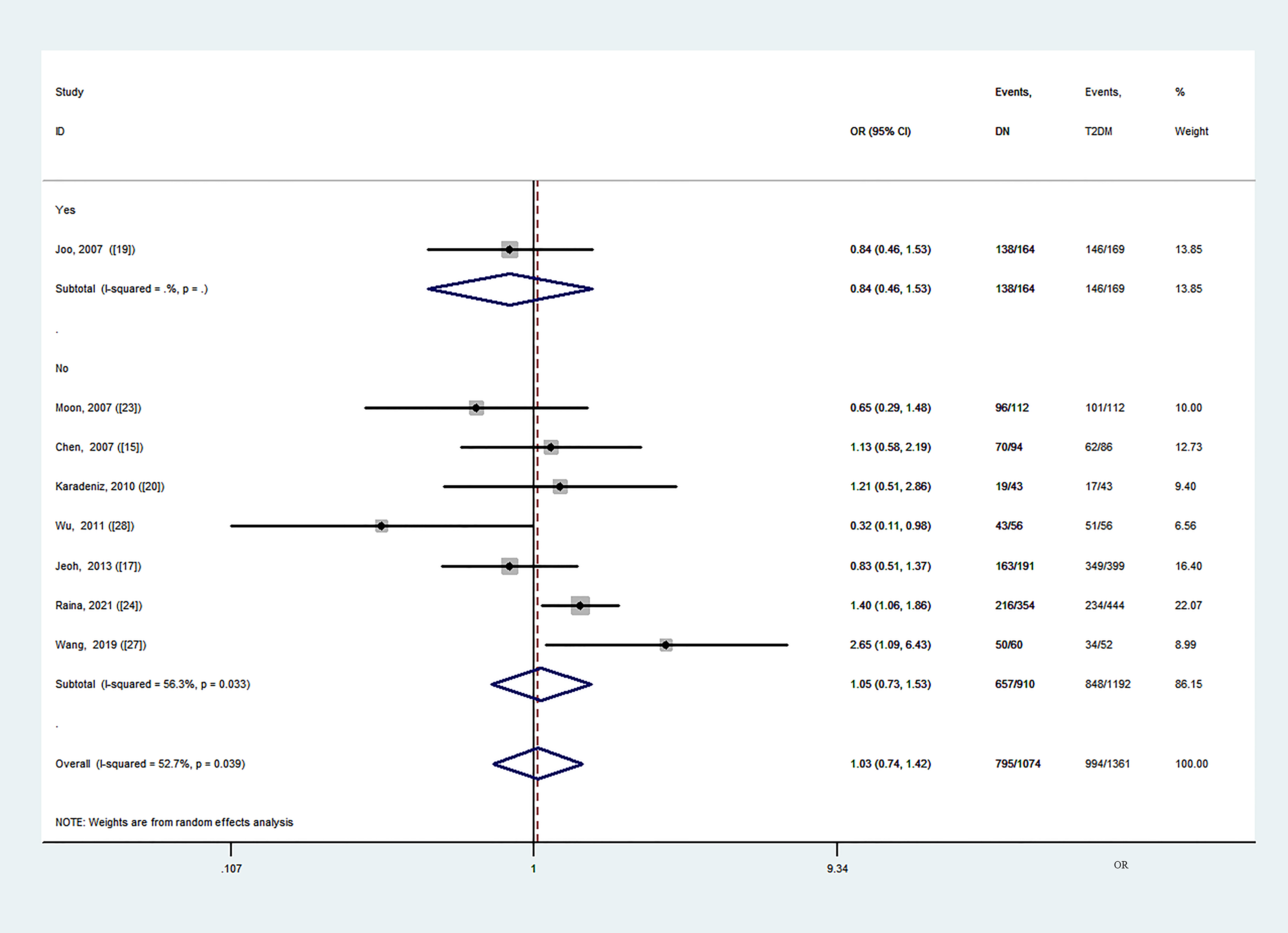

Supplement: Supplementary file 1 — Additional file 1: Figure S1. Forest plot of T2DM risk with the dominant model (GG+GA vs. AA) (T2DM vs. healthy control) of the MCP-1 rs1024611 polymorphism. (A) for the overall populations and (B) genotyping method; (C) age- and sex- adjusted; (D) and comorbid chronic disease subgroups. Figure S2. Forest plot of DN risk with the dominant model (GG+GA vs. AA) (DN vs. healthy control) of the MCP-1 rs1024611 polymorphism. (A) for the overall populations and (B) genotyping method; (C) age- and sex- adjusted; (D) and comorbid chronic disease subgroups. Figure S3. Forest plot of DN risk with the dominant model (GG+GA vs. AA) (DN vs. T2DM) of the MCP-1 rs1024611 polymorphism. (A) for the overall populations and (B) genotyping method; (C) age- and sex- adjusted; (D) and comorbid chronic disease subgroups. Figure S4. Sensitivity analysis via deletion of each individual study (T2DM vs. healthy control). (A) for GG+GA vs. AA and (B) GG vs.GA + AA; (C) GG vs. AA; (D) GG vs. GA; (E) and G vs. A models. Figure S5. Sensitivity analysis via deletion of each individual study (DN vs. healthy control). (A) for GG+GA vs. AA and (B) GG vs.GA + AA; (C) GG vs. AA; (D) GG vs. GA; (E) and G vs. A models. Figure S6. Sensitivity analysis via deletion of each individual study (DN vs. T2DM). (A) for GG+GA vs. AA and (B) GG vs.GA + AA; (C) GG vs. AA; (D) GG vs. GA; (E) and G vs. A models. Table S1. The comprehensive search strategies for different databases. Table S2. Main characteristic of included observational studies evaluating the relationship between the MCP-1 rs1024611 polymorphism and DN/T2DM risk. Table S3. Meta-analysis of the association between the MCP-1 rs1024611polymorphism and T2DM risk (T2DM vs. healthy control). Table S4. Meta-analysis of the association between the MCP-1 rs1024611polymorphism and DN risk (DN vs. healthy control). Table S5. Meta-analysis of the association between the MCP-1 rs1024611polymorphism and DN risk (DN vs. T2DM). [file 12902_2023_1514_MOESM1_ESM.zip › Figure S3/Figure S3D.jpg]

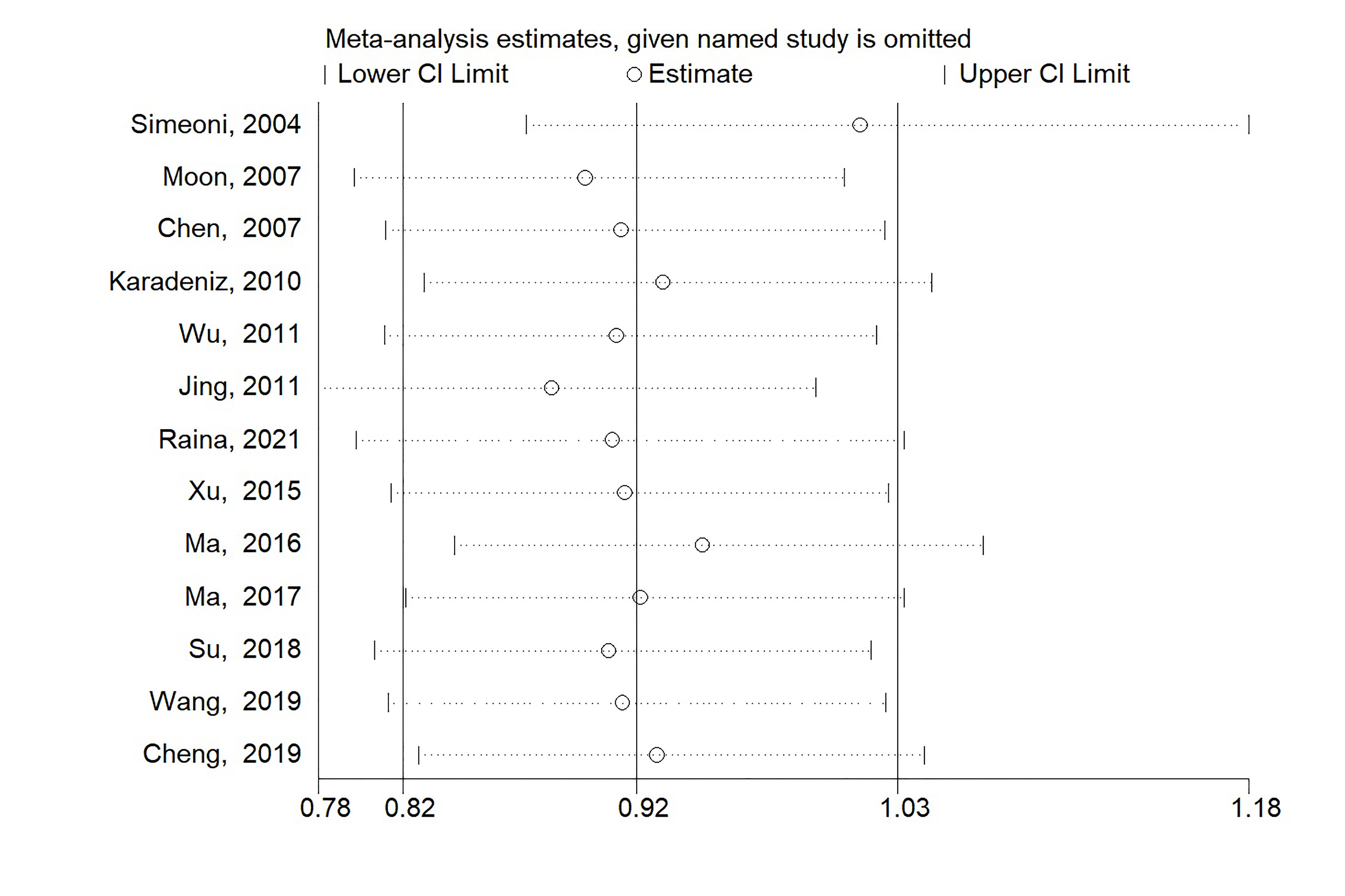

Supplement: Supplementary file 1 — Additional file 1: Figure S1. Forest plot of T2DM risk with the dominant model (GG+GA vs. AA) (T2DM vs. healthy control) of the MCP-1 rs1024611 polymorphism. (A) for the overall populations and (B) genotyping method; (C) age- and sex- adjusted; (D) and comorbid chronic disease subgroups. Figure S2. Forest plot of DN risk with the dominant model (GG+GA vs. AA) (DN vs. healthy control) of the MCP-1 rs1024611 polymorphism. (A) for the overall populations and (B) genotyping method; (C) age- and sex- adjusted; (D) and comorbid chronic disease subgroups. Figure S3. Forest plot of DN risk with the dominant model (GG+GA vs. AA) (DN vs. T2DM) of the MCP-1 rs1024611 polymorphism. (A) for the overall populations and (B) genotyping method; (C) age- and sex- adjusted; (D) and comorbid chronic disease subgroups. Figure S4. Sensitivity analysis via deletion of each individual study (T2DM vs. healthy control). (A) for GG+GA vs. AA and (B) GG vs.GA + AA; (C) GG vs. AA; (D) GG vs. GA; (E) and G vs. A models. Figure S5. Sensitivity analysis via deletion of each individual study (DN vs. healthy control). (A) for GG+GA vs. AA and (B) GG vs.GA + AA; (C) GG vs. AA; (D) GG vs. GA; (E) and G vs. A models. Figure S6. Sensitivity analysis via deletion of each individual study (DN vs. T2DM). (A) for GG+GA vs. AA and (B) GG vs.GA + AA; (C) GG vs. AA; (D) GG vs. GA; (E) and G vs. A models. Table S1. The comprehensive search strategies for different databases. Table S2. Main characteristic of included observational studies evaluating the relationship between the MCP-1 rs1024611 polymorphism and DN/T2DM risk. Table S3. Meta-analysis of the association between the MCP-1 rs1024611polymorphism and T2DM risk (T2DM vs. healthy control). Table S4. Meta-analysis of the association between the MCP-1 rs1024611polymorphism and DN risk (DN vs. healthy control). Table S5. Meta-analysis of the association between the MCP-1 rs1024611polymorphism and DN risk (DN vs. T2DM). [file 12902_2023_1514_MOESM1_ESM.zip › Figure S4/Figure S4A.jpg]

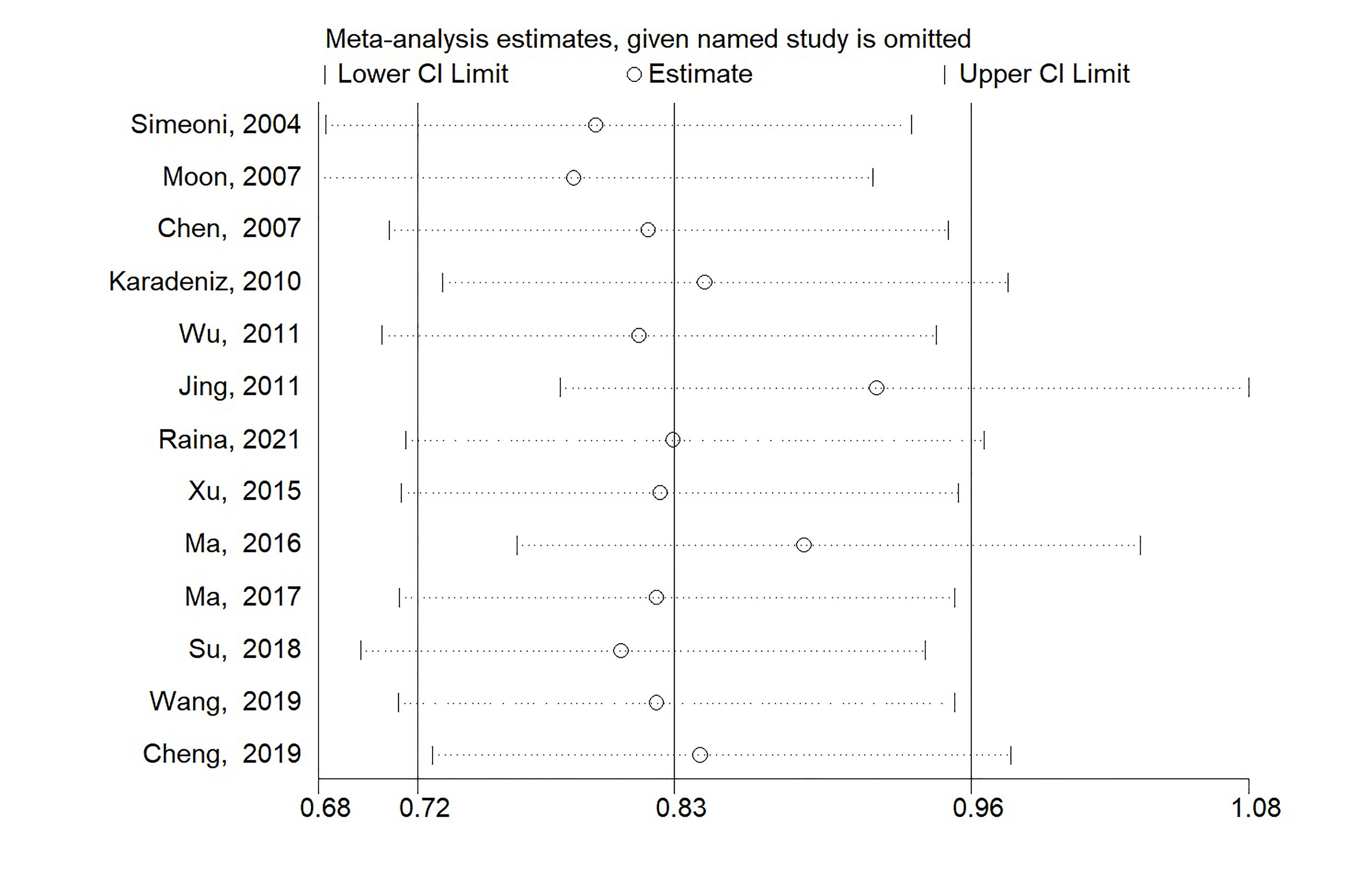

Supplement: Supplementary file 1 — Additional file 1: Figure S1. Forest plot of T2DM risk with the dominant model (GG+GA vs. AA) (T2DM vs. healthy control) of the MCP-1 rs1024611 polymorphism. (A) for the overall populations and (B) genotyping method; (C) age- and sex- adjusted; (D) and comorbid chronic disease subgroups. Figure S2. Forest plot of DN risk with the dominant model (GG+GA vs. AA) (DN vs. healthy control) of the MCP-1 rs1024611 polymorphism. (A) for the overall populations and (B) genotyping method; (C) age- and sex- adjusted; (D) and comorbid chronic disease subgroups. Figure S3. Forest plot of DN risk with the dominant model (GG+GA vs. AA) (DN vs. T2DM) of the MCP-1 rs1024611 polymorphism. (A) for the overall populations and (B) genotyping method; (C) age- and sex- adjusted; (D) and comorbid chronic disease subgroups. Figure S4. Sensitivity analysis via deletion of each individual study (T2DM vs. healthy control). (A) for GG+GA vs. AA and (B) GG vs.GA + AA; (C) GG vs. AA; (D) GG vs. GA; (E) and G vs. A models. Figure S5. Sensitivity analysis via deletion of each individual study (DN vs. healthy control). (A) for GG+GA vs. AA and (B) GG vs.GA + AA; (C) GG vs. AA; (D) GG vs. GA; (E) and G vs. A models. Figure S6. Sensitivity analysis via deletion of each individual study (DN vs. T2DM). (A) for GG+GA vs. AA and (B) GG vs.GA + AA; (C) GG vs. AA; (D) GG vs. GA; (E) and G vs. A models. Table S1. The comprehensive search strategies for different databases. Table S2. Main characteristic of included observational studies evaluating the relationship between the MCP-1 rs1024611 polymorphism and DN/T2DM risk. Table S3. Meta-analysis of the association between the MCP-1 rs1024611polymorphism and T2DM risk (T2DM vs. healthy control). Table S4. Meta-analysis of the association between the MCP-1 rs1024611polymorphism and DN risk (DN vs. healthy control). Table S5. Meta-analysis of the association between the MCP-1 rs1024611polymorphism and DN risk (DN vs. T2DM). [file 12902_2023_1514_MOESM1_ESM.zip › Figure S4/Figure S4B.jpg]

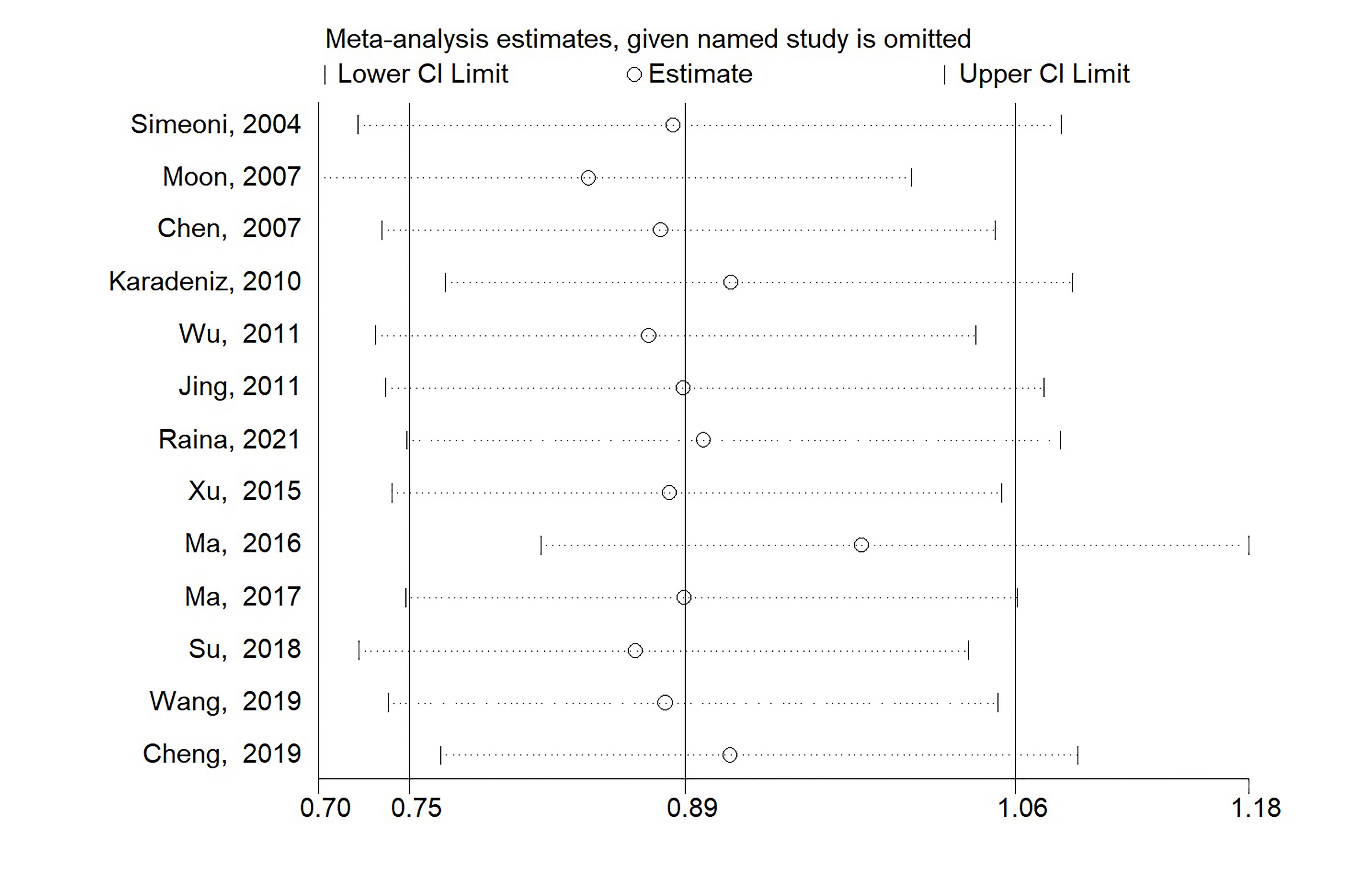

Supplement: Supplementary file 1 — Additional file 1: Figure S1. Forest plot of T2DM risk with the dominant model (GG+GA vs. AA) (T2DM vs. healthy control) of the MCP-1 rs1024611 polymorphism. (A) for the overall populations and (B) genotyping method; (C) age- and sex- adjusted; (D) and comorbid chronic disease subgroups. Figure S2. Forest plot of DN risk with the dominant model (GG+GA vs. AA) (DN vs. healthy control) of the MCP-1 rs1024611 polymorphism. (A) for the overall populations and (B) genotyping method; (C) age- and sex- adjusted; (D) and comorbid chronic disease subgroups. Figure S3. Forest plot of DN risk with the dominant model (GG+GA vs. AA) (DN vs. T2DM) of the MCP-1 rs1024611 polymorphism. (A) for the overall populations and (B) genotyping method; (C) age- and sex- adjusted; (D) and comorbid chronic disease subgroups. Figure S4. Sensitivity analysis via deletion of each individual study (T2DM vs. healthy control). (A) for GG+GA vs. AA and (B) GG vs.GA + AA; (C) GG vs. AA; (D) GG vs. GA; (E) and G vs. A models. Figure S5. Sensitivity analysis via deletion of each individual study (DN vs. healthy control). (A) for GG+GA vs. AA and (B) GG vs.GA + AA; (C) GG vs. AA; (D) GG vs. GA; (E) and G vs. A models. Figure S6. Sensitivity analysis via deletion of each individual study (DN vs. T2DM). (A) for GG+GA vs. AA and (B) GG vs.GA + AA; (C) GG vs. AA; (D) GG vs. GA; (E) and G vs. A models. Table S1. The comprehensive search strategies for different databases. Table S2. Main characteristic of included observational studies evaluating the relationship between the MCP-1 rs1024611 polymorphism and DN/T2DM risk. Table S3. Meta-analysis of the association between the MCP-1 rs1024611polymorphism and T2DM risk (T2DM vs. healthy control). Table S4. Meta-analysis of the association between the MCP-1 rs1024611polymorphism and DN risk (DN vs. healthy control). Table S5. Meta-analysis of the association between the MCP-1 rs1024611polymorphism and DN risk (DN vs. T2DM). [file 12902_2023_1514_MOESM1_ESM.zip › Figure S4/Figure S4C.jpg]

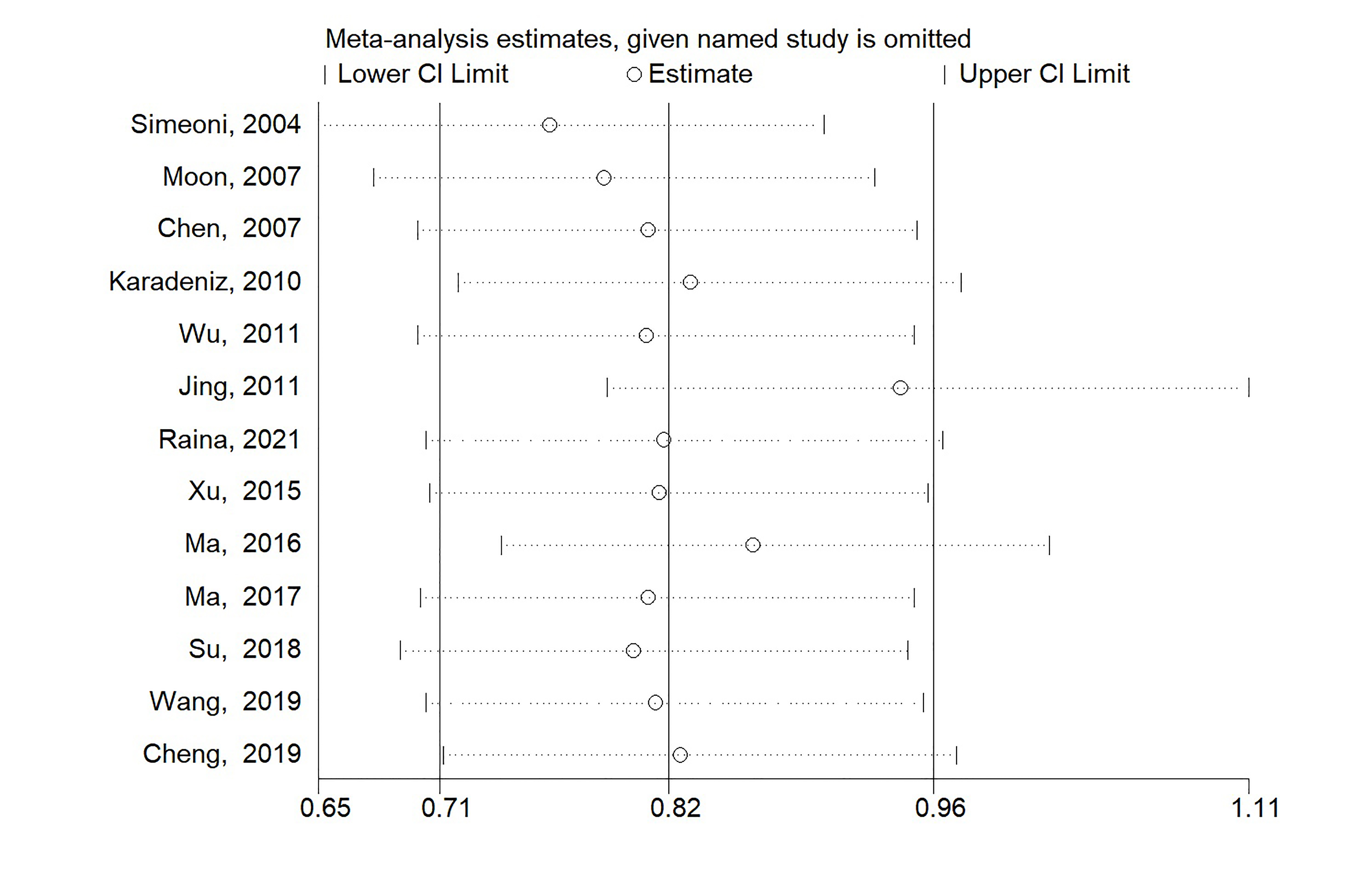

Supplement: Supplementary file 1 — Additional file 1: Figure S1. Forest plot of T2DM risk with the dominant model (GG+GA vs. AA) (T2DM vs. healthy control) of the MCP-1 rs1024611 polymorphism. (A) for the overall populations and (B) genotyping method; (C) age- and sex- adjusted; (D) and comorbid chronic disease subgroups. Figure S2. Forest plot of DN risk with the dominant model (GG+GA vs. AA) (DN vs. healthy control) of the MCP-1 rs1024611 polymorphism. (A) for the overall populations and (B) genotyping method; (C) age- and sex- adjusted; (D) and comorbid chronic disease subgroups. Figure S3. Forest plot of DN risk with the dominant model (GG+GA vs. AA) (DN vs. T2DM) of the MCP-1 rs1024611 polymorphism. (A) for the overall populations and (B) genotyping method; (C) age- and sex- adjusted; (D) and comorbid chronic disease subgroups. Figure S4. Sensitivity analysis via deletion of each individual study (T2DM vs. healthy control). (A) for GG+GA vs. AA and (B) GG vs.GA + AA; (C) GG vs. AA; (D) GG vs. GA; (E) and G vs. A models. Figure S5. Sensitivity analysis via deletion of each individual study (DN vs. healthy control). (A) for GG+GA vs. AA and (B) GG vs.GA + AA; (C) GG vs. AA; (D) GG vs. GA; (E) and G vs. A models. Figure S6. Sensitivity analysis via deletion of each individual study (DN vs. T2DM). (A) for GG+GA vs. AA and (B) GG vs.GA + AA; (C) GG vs. AA; (D) GG vs. GA; (E) and G vs. A models. Table S1. The comprehensive search strategies for different databases. Table S2. Main characteristic of included observational studies evaluating the relationship between the MCP-1 rs1024611 polymorphism and DN/T2DM risk. Table S3. Meta-analysis of the association between the MCP-1 rs1024611polymorphism and T2DM risk (T2DM vs. healthy control). Table S4. Meta-analysis of the association between the MCP-1 rs1024611polymorphism and DN risk (DN vs. healthy control). Table S5. Meta-analysis of the association between the MCP-1 rs1024611polymorphism and DN risk (DN vs. T2DM). [file 12902_2023_1514_MOESM1_ESM.zip › Figure S4/Figure S4D.jpg]

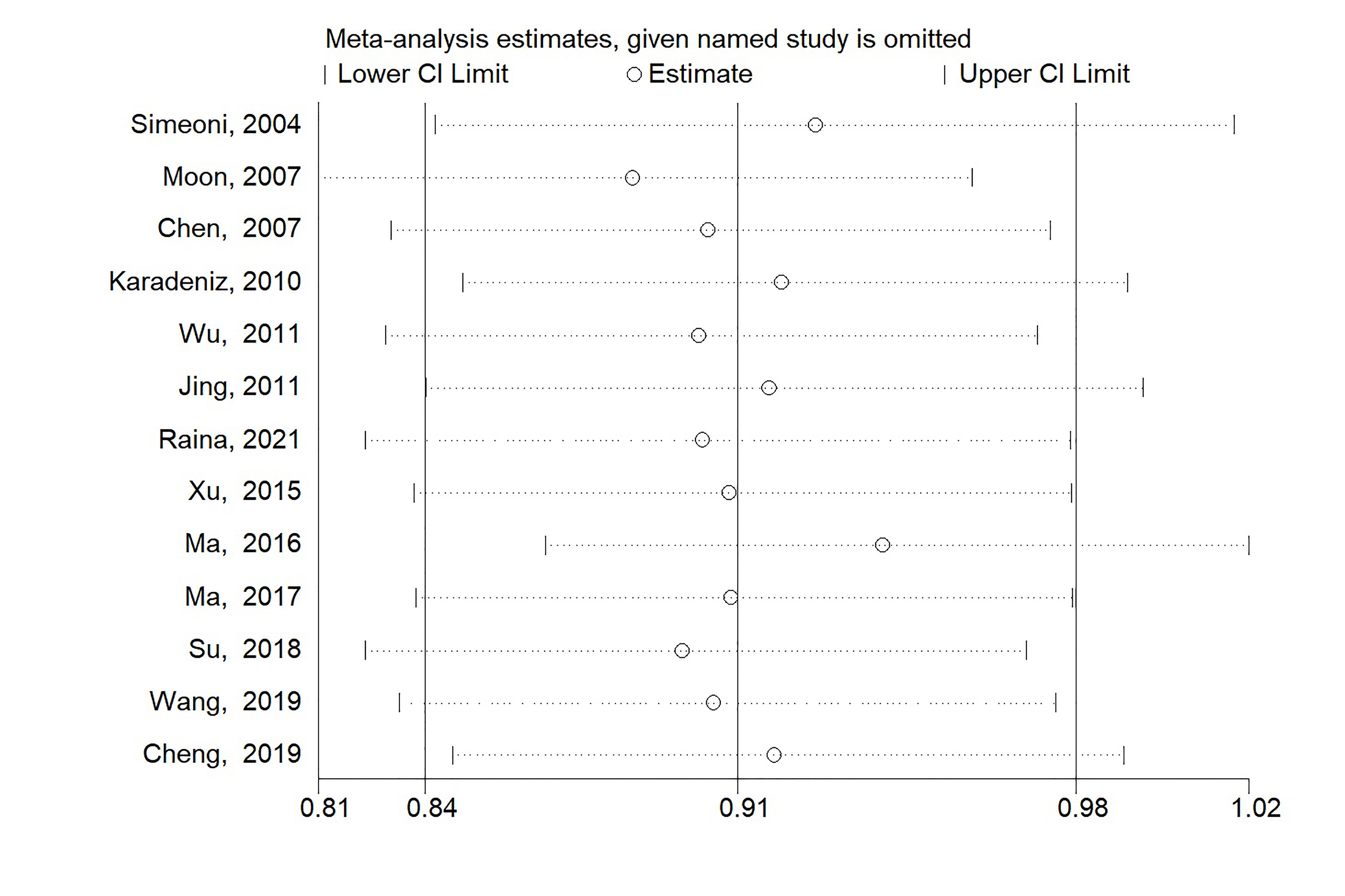

Supplement: Supplementary file 1 — Additional file 1: Figure S1. Forest plot of T2DM risk with the dominant model (GG+GA vs. AA) (T2DM vs. healthy control) of the MCP-1 rs1024611 polymorphism. (A) for the overall populations and (B) genotyping method; (C) age- and sex- adjusted; (D) and comorbid chronic disease subgroups. Figure S2. Forest plot of DN risk with the dominant model (GG+GA vs. AA) (DN vs. healthy control) of the MCP-1 rs1024611 polymorphism. (A) for the overall populations and (B) genotyping method; (C) age- and sex- adjusted; (D) and comorbid chronic disease subgroups. Figure S3. Forest plot of DN risk with the dominant model (GG+GA vs. AA) (DN vs. T2DM) of the MCP-1 rs1024611 polymorphism. (A) for the overall populations and (B) genotyping method; (C) age- and sex- adjusted; (D) and comorbid chronic disease subgroups. Figure S4. Sensitivity analysis via deletion of each individual study (T2DM vs. healthy control). (A) for GG+GA vs. AA and (B) GG vs.GA + AA; (C) GG vs. AA; (D) GG vs. GA; (E) and G vs. A models. Figure S5. Sensitivity analysis via deletion of each individual study (DN vs. healthy control). (A) for GG+GA vs. AA and (B) GG vs.GA + AA; (C) GG vs. AA; (D) GG vs. GA; (E) and G vs. A models. Figure S6. Sensitivity analysis via deletion of each individual study (DN vs. T2DM). (A) for GG+GA vs. AA and (B) GG vs.GA + AA; (C) GG vs. AA; (D) GG vs. GA; (E) and G vs. A models. Table S1. The comprehensive search strategies for different databases. Table S2. Main characteristic of included observational studies evaluating the relationship between the MCP-1 rs1024611 polymorphism and DN/T2DM risk. Table S3. Meta-analysis of the association between the MCP-1 rs1024611polymorphism and T2DM risk (T2DM vs. healthy control). Table S4. Meta-analysis of the association between the MCP-1 rs1024611polymorphism and DN risk (DN vs. healthy control). Table S5. Meta-analysis of the association between the MCP-1 rs1024611polymorphism and DN risk (DN vs. T2DM). [file 12902_2023_1514_MOESM1_ESM.zip › Figure S4/Figure S4E.jpg]

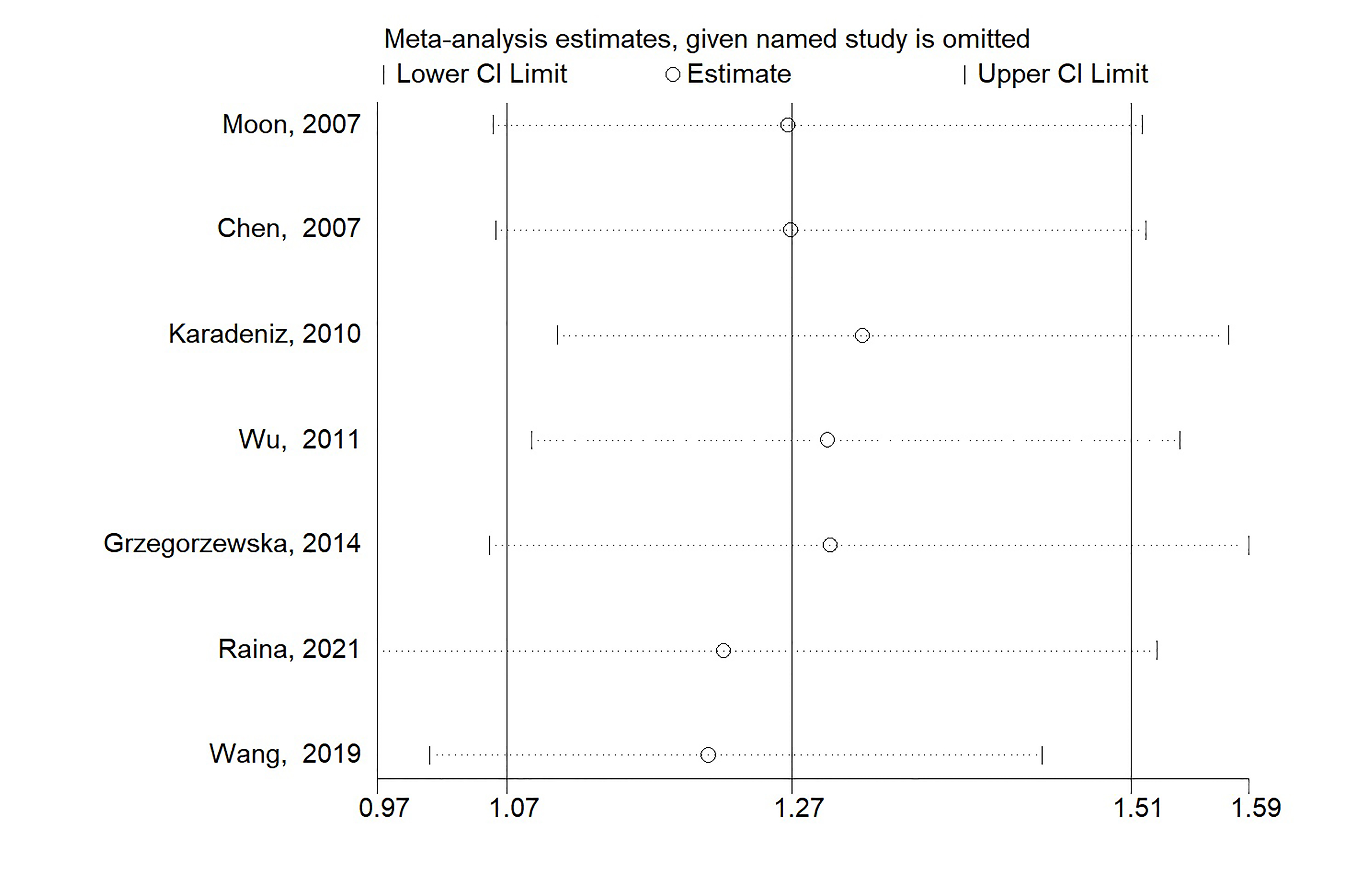

Supplement: Supplementary file 1 — Additional file 1: Figure S1. Forest plot of T2DM risk with the dominant model (GG+GA vs. AA) (T2DM vs. healthy control) of the MCP-1 rs1024611 polymorphism. (A) for the overall populations and (B) genotyping method; (C) age- and sex- adjusted; (D) and comorbid chronic disease subgroups. Figure S2. Forest plot of DN risk with the dominant model (GG+GA vs. AA) (DN vs. healthy control) of the MCP-1 rs1024611 polymorphism. (A) for the overall populations and (B) genotyping method; (C) age- and sex- adjusted; (D) and comorbid chronic disease subgroups. Figure S3. Forest plot of DN risk with the dominant model (GG+GA vs. AA) (DN vs. T2DM) of the MCP-1 rs1024611 polymorphism. (A) for the overall populations and (B) genotyping method; (C) age- and sex- adjusted; (D) and comorbid chronic disease subgroups. Figure S4. Sensitivity analysis via deletion of each individual study (T2DM vs. healthy control). (A) for GG+GA vs. AA and (B) GG vs.GA + AA; (C) GG vs. AA; (D) GG vs. GA; (E) and G vs. A models. Figure S5. Sensitivity analysis via deletion of each individual study (DN vs. healthy control). (A) for GG+GA vs. AA and (B) GG vs.GA + AA; (C) GG vs. AA; (D) GG vs. GA; (E) and G vs. A models. Figure S6. Sensitivity analysis via deletion of each individual study (DN vs. T2DM). (A) for GG+GA vs. AA and (B) GG vs.GA + AA; (C) GG vs. AA; (D) GG vs. GA; (E) and G vs. A models. Table S1. The comprehensive search strategies for different databases. Table S2. Main characteristic of included observational studies evaluating the relationship between the MCP-1 rs1024611 polymorphism and DN/T2DM risk. Table S3. Meta-analysis of the association between the MCP-1 rs1024611polymorphism and T2DM risk (T2DM vs. healthy control). Table S4. Meta-analysis of the association between the MCP-1 rs1024611polymorphism and DN risk (DN vs. healthy control). Table S5. Meta-analysis of the association between the MCP-1 rs1024611polymorphism and DN risk (DN vs. T2DM). [file 12902_2023_1514_MOESM1_ESM.zip › Figure S5/Figure S5A.jpg]

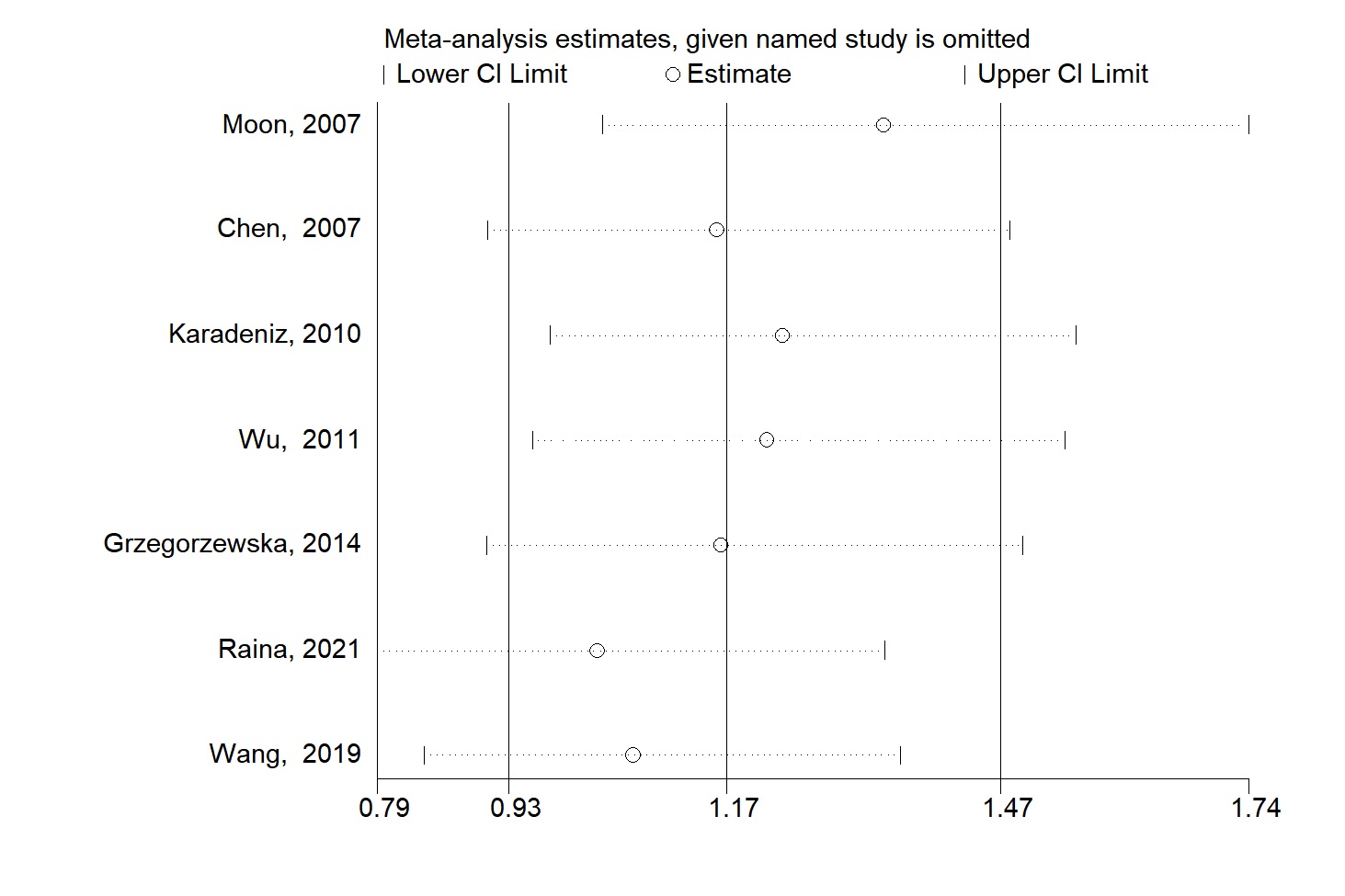

Supplement: Supplementary file 1 — Additional file 1: Figure S1. Forest plot of T2DM risk with the dominant model (GG+GA vs. AA) (T2DM vs. healthy control) of the MCP-1 rs1024611 polymorphism. (A) for the overall populations and (B) genotyping method; (C) age- and sex- adjusted; (D) and comorbid chronic disease subgroups. Figure S2. Forest plot of DN risk with the dominant model (GG+GA vs. AA) (DN vs. healthy control) of the MCP-1 rs1024611 polymorphism. (A) for the overall populations and (B) genotyping method; (C) age- and sex- adjusted; (D) and comorbid chronic disease subgroups. Figure S3. Forest plot of DN risk with the dominant model (GG+GA vs. AA) (DN vs. T2DM) of the MCP-1 rs1024611 polymorphism. (A) for the overall populations and (B) genotyping method; (C) age- and sex- adjusted; (D) and comorbid chronic disease subgroups. Figure S4. Sensitivity analysis via deletion of each individual study (T2DM vs. healthy control). (A) for GG+GA vs. AA and (B) GG vs.GA + AA; (C) GG vs. AA; (D) GG vs. GA; (E) and G vs. A models. Figure S5. Sensitivity analysis via deletion of each individual study (DN vs. healthy control). (A) for GG+GA vs. AA and (B) GG vs.GA + AA; (C) GG vs. AA; (D) GG vs. GA; (E) and G vs. A models. Figure S6. Sensitivity analysis via deletion of each individual study (DN vs. T2DM). (A) for GG+GA vs. AA and (B) GG vs.GA + AA; (C) GG vs. AA; (D) GG vs. GA; (E) and G vs. A models. Table S1. The comprehensive search strategies for different databases. Table S2. Main characteristic of included observational studies evaluating the relationship between the MCP-1 rs1024611 polymorphism and DN/T2DM risk. Table S3. Meta-analysis of the association between the MCP-1 rs1024611polymorphism and T2DM risk (T2DM vs. healthy control). Table S4. Meta-analysis of the association between the MCP-1 rs1024611polymorphism and DN risk (DN vs. healthy control). Table S5. Meta-analysis of the association between the MCP-1 rs1024611polymorphism and DN risk (DN vs. T2DM). [file 12902_2023_1514_MOESM1_ESM.zip › Figure S5/Figure S5B.jpg]

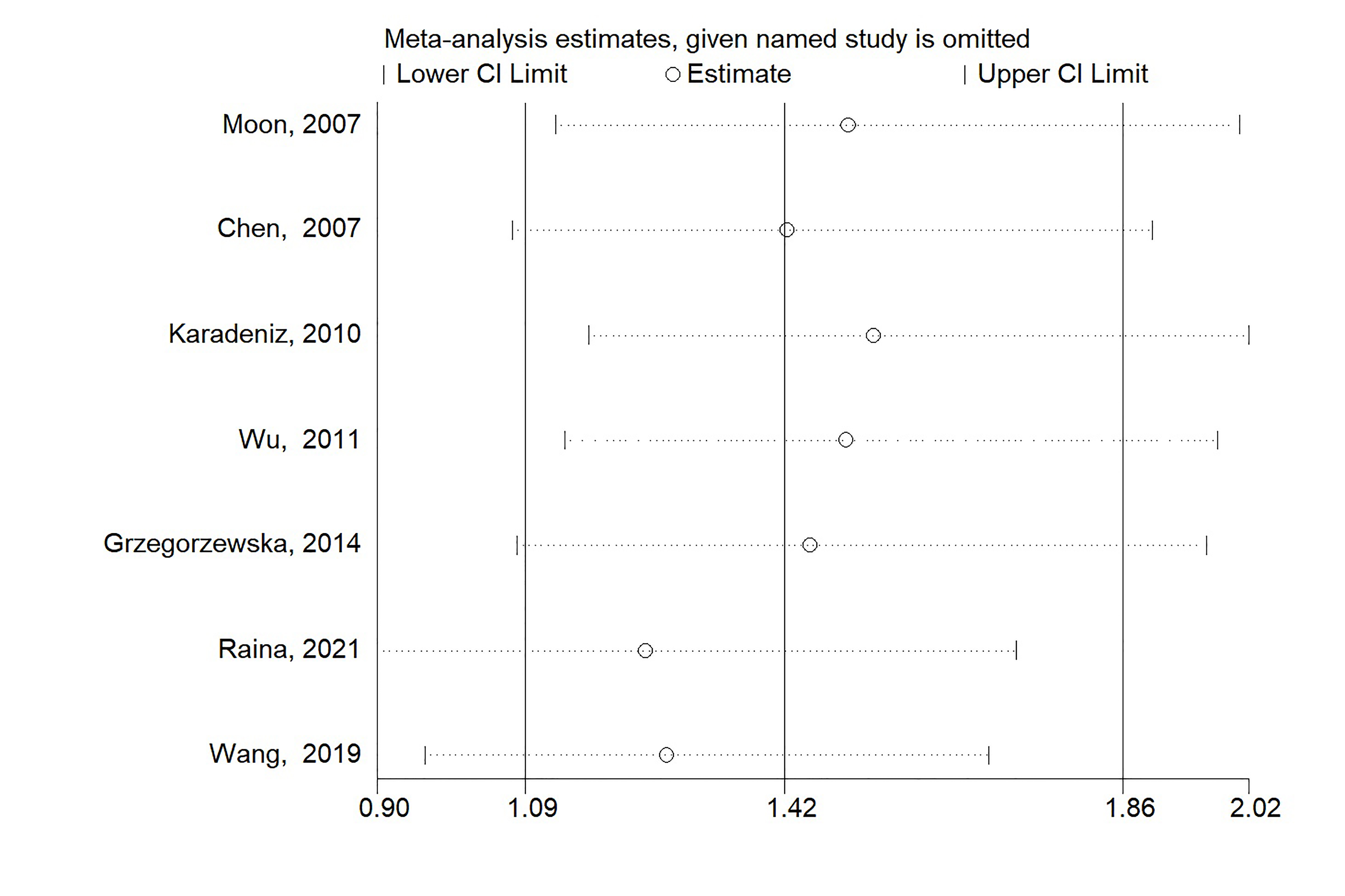

Supplement: Supplementary file 1 — Additional file 1: Figure S1. Forest plot of T2DM risk with the dominant model (GG+GA vs. AA) (T2DM vs. healthy control) of the MCP-1 rs1024611 polymorphism. (A) for the overall populations and (B) genotyping method; (C) age- and sex- adjusted; (D) and comorbid chronic disease subgroups. Figure S2. Forest plot of DN risk with the dominant model (GG+GA vs. AA) (DN vs. healthy control) of the MCP-1 rs1024611 polymorphism. (A) for the overall populations and (B) genotyping method; (C) age- and sex- adjusted; (D) and comorbid chronic disease subgroups. Figure S3. Forest plot of DN risk with the dominant model (GG+GA vs. AA) (DN vs. T2DM) of the MCP-1 rs1024611 polymorphism. (A) for the overall populations and (B) genotyping method; (C) age- and sex- adjusted; (D) and comorbid chronic disease subgroups. Figure S4. Sensitivity analysis via deletion of each individual study (T2DM vs. healthy control). (A) for GG+GA vs. AA and (B) GG vs.GA + AA; (C) GG vs. AA; (D) GG vs. GA; (E) and G vs. A models. Figure S5. Sensitivity analysis via deletion of each individual study (DN vs. healthy control). (A) for GG+GA vs. AA and (B) GG vs.GA + AA; (C) GG vs. AA; (D) GG vs. GA; (E) and G vs. A models. Figure S6. Sensitivity analysis via deletion of each individual study (DN vs. T2DM). (A) for GG+GA vs. AA and (B) GG vs.GA + AA; (C) GG vs. AA; (D) GG vs. GA; (E) and G vs. A models. Table S1. The comprehensive search strategies for different databases. Table S2. Main characteristic of included observational studies evaluating the relationship between the MCP-1 rs1024611 polymorphism and DN/T2DM risk. Table S3. Meta-analysis of the association between the MCP-1 rs1024611polymorphism and T2DM risk (T2DM vs. healthy control). Table S4. Meta-analysis of the association between the MCP-1 rs1024611polymorphism and DN risk (DN vs. healthy control). Table S5. Meta-analysis of the association between the MCP-1 rs1024611polymorphism and DN risk (DN vs. T2DM). [file 12902_2023_1514_MOESM1_ESM.zip › Figure S5/Figure S5C.jpg]

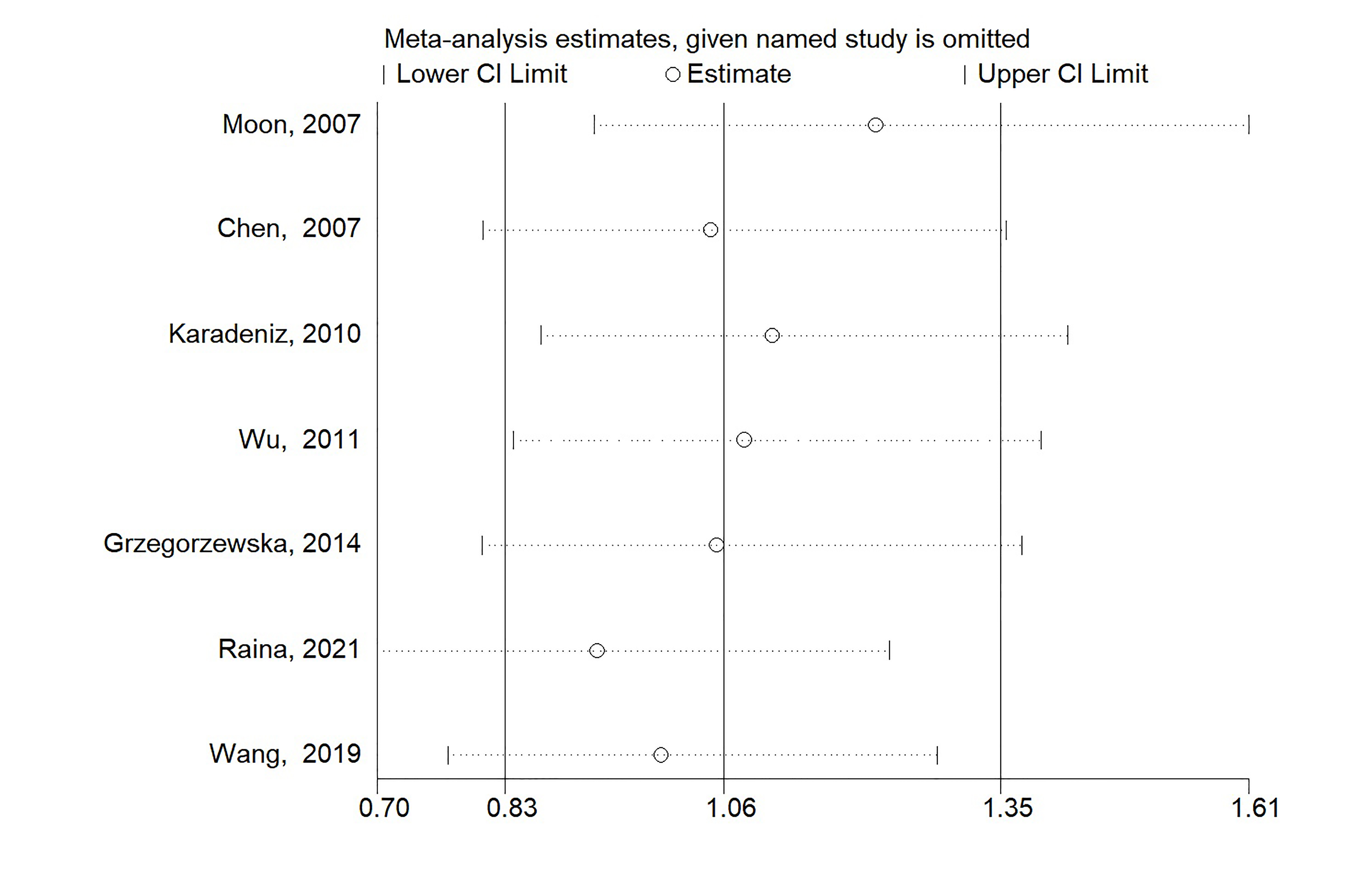

Supplement: Supplementary file 1 — Additional file 1: Figure S1. Forest plot of T2DM risk with the dominant model (GG+GA vs. AA) (T2DM vs. healthy control) of the MCP-1 rs1024611 polymorphism. (A) for the overall populations and (B) genotyping method; (C) age- and sex- adjusted; (D) and comorbid chronic disease subgroups. Figure S2. Forest plot of DN risk with the dominant model (GG+GA vs. AA) (DN vs. healthy control) of the MCP-1 rs1024611 polymorphism. (A) for the overall populations and (B) genotyping method; (C) age- and sex- adjusted; (D) and comorbid chronic disease subgroups. Figure S3. Forest plot of DN risk with the dominant model (GG+GA vs. AA) (DN vs. T2DM) of the MCP-1 rs1024611 polymorphism. (A) for the overall populations and (B) genotyping method; (C) age- and sex- adjusted; (D) and comorbid chronic disease subgroups. Figure S4. Sensitivity analysis via deletion of each individual study (T2DM vs. healthy control). (A) for GG+GA vs. AA and (B) GG vs.GA + AA; (C) GG vs. AA; (D) GG vs. GA; (E) and G vs. A models. Figure S5. Sensitivity analysis via deletion of each individual study (DN vs. healthy control). (A) for GG+GA vs. AA and (B) GG vs.GA + AA; (C) GG vs. AA; (D) GG vs. GA; (E) and G vs. A models. Figure S6. Sensitivity analysis via deletion of each individual study (DN vs. T2DM). (A) for GG+GA vs. AA and (B) GG vs.GA + AA; (C) GG vs. AA; (D) GG vs. GA; (E) and G vs. A models. Table S1. The comprehensive search strategies for different databases. Table S2. Main characteristic of included observational studies evaluating the relationship between the MCP-1 rs1024611 polymorphism and DN/T2DM risk. Table S3. Meta-analysis of the association between the MCP-1 rs1024611polymorphism and T2DM risk (T2DM vs. healthy control). Table S4. Meta-analysis of the association between the MCP-1 rs1024611polymorphism and DN risk (DN vs. healthy control). Table S5. Meta-analysis of the association between the MCP-1 rs1024611polymorphism and DN risk (DN vs. T2DM). [file 12902_2023_1514_MOESM1_ESM.zip › Figure S5/Figure S5D.jpg]

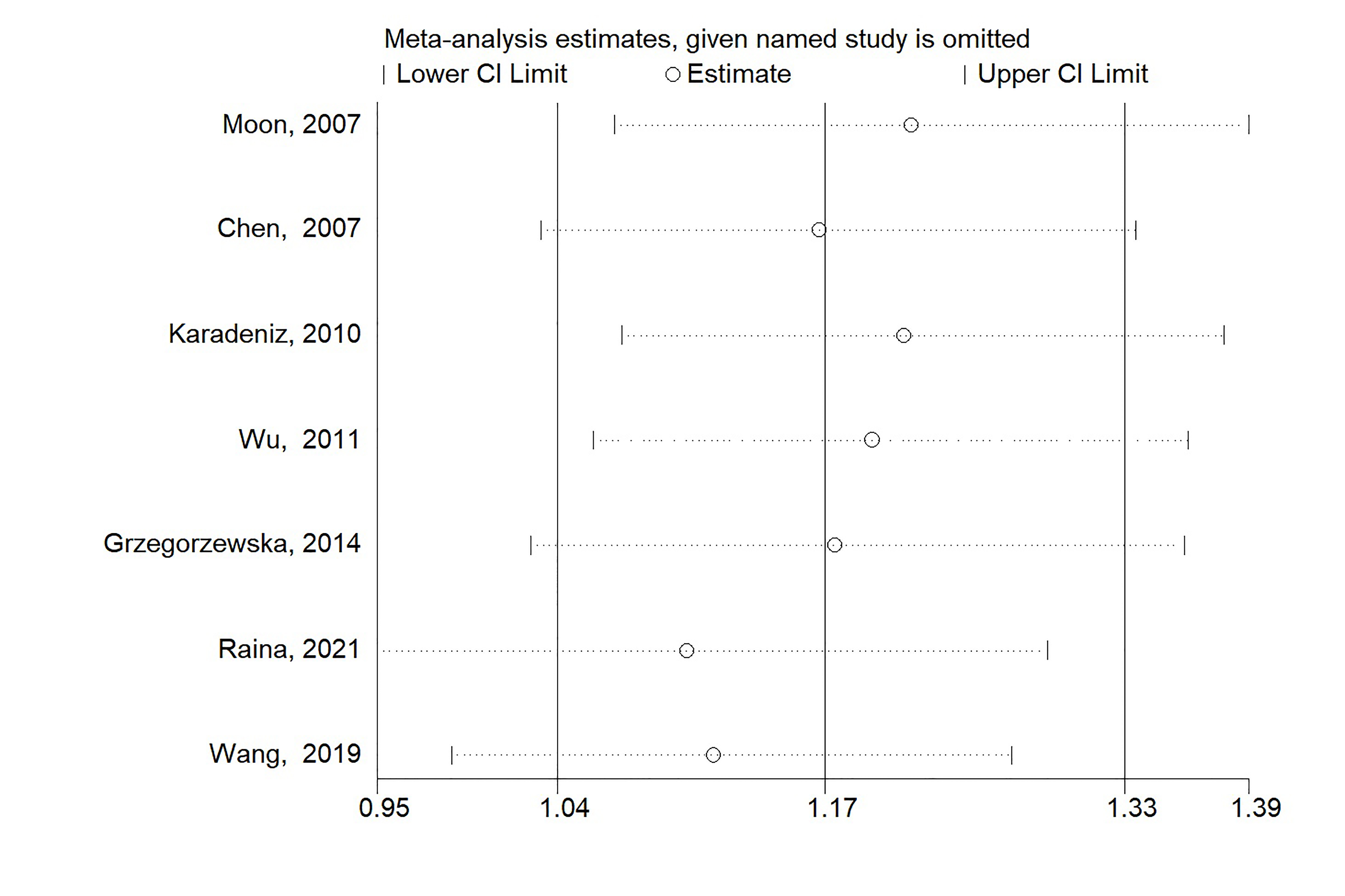

Supplement: Supplementary file 1 — Additional file 1: Figure S1. Forest plot of T2DM risk with the dominant model (GG+GA vs. AA) (T2DM vs. healthy control) of the MCP-1 rs1024611 polymorphism. (A) for the overall populations and (B) genotyping method; (C) age- and sex- adjusted; (D) and comorbid chronic disease subgroups. Figure S2. Forest plot of DN risk with the dominant model (GG+GA vs. AA) (DN vs. healthy control) of the MCP-1 rs1024611 polymorphism. (A) for the overall populations and (B) genotyping method; (C) age- and sex- adjusted; (D) and comorbid chronic disease subgroups. Figure S3. Forest plot of DN risk with the dominant model (GG+GA vs. AA) (DN vs. T2DM) of the MCP-1 rs1024611 polymorphism. (A) for the overall populations and (B) genotyping method; (C) age- and sex- adjusted; (D) and comorbid chronic disease subgroups. Figure S4. Sensitivity analysis via deletion of each individual study (T2DM vs. healthy control). (A) for GG+GA vs. AA and (B) GG vs.GA + AA; (C) GG vs. AA; (D) GG vs. GA; (E) and G vs. A models. Figure S5. Sensitivity analysis via deletion of each individual study (DN vs. healthy control). (A) for GG+GA vs. AA and (B) GG vs.GA + AA; (C) GG vs. AA; (D) GG vs. GA; (E) and G vs. A models. Figure S6. Sensitivity analysis via deletion of each individual study (DN vs. T2DM). (A) for GG+GA vs. AA and (B) GG vs.GA + AA; (C) GG vs. AA; (D) GG vs. GA; (E) and G vs. A models. Table S1. The comprehensive search strategies for different databases. Table S2. Main characteristic of included observational studies evaluating the relationship between the MCP-1 rs1024611 polymorphism and DN/T2DM risk. Table S3. Meta-analysis of the association between the MCP-1 rs1024611polymorphism and T2DM risk (T2DM vs. healthy control). Table S4. Meta-analysis of the association between the MCP-1 rs1024611polymorphism and DN risk (DN vs. healthy control). Table S5. Meta-analysis of the association between the MCP-1 rs1024611polymorphism and DN risk (DN vs. T2DM). [file 12902_2023_1514_MOESM1_ESM.zip › Figure S5/Figure S5E.jpg]

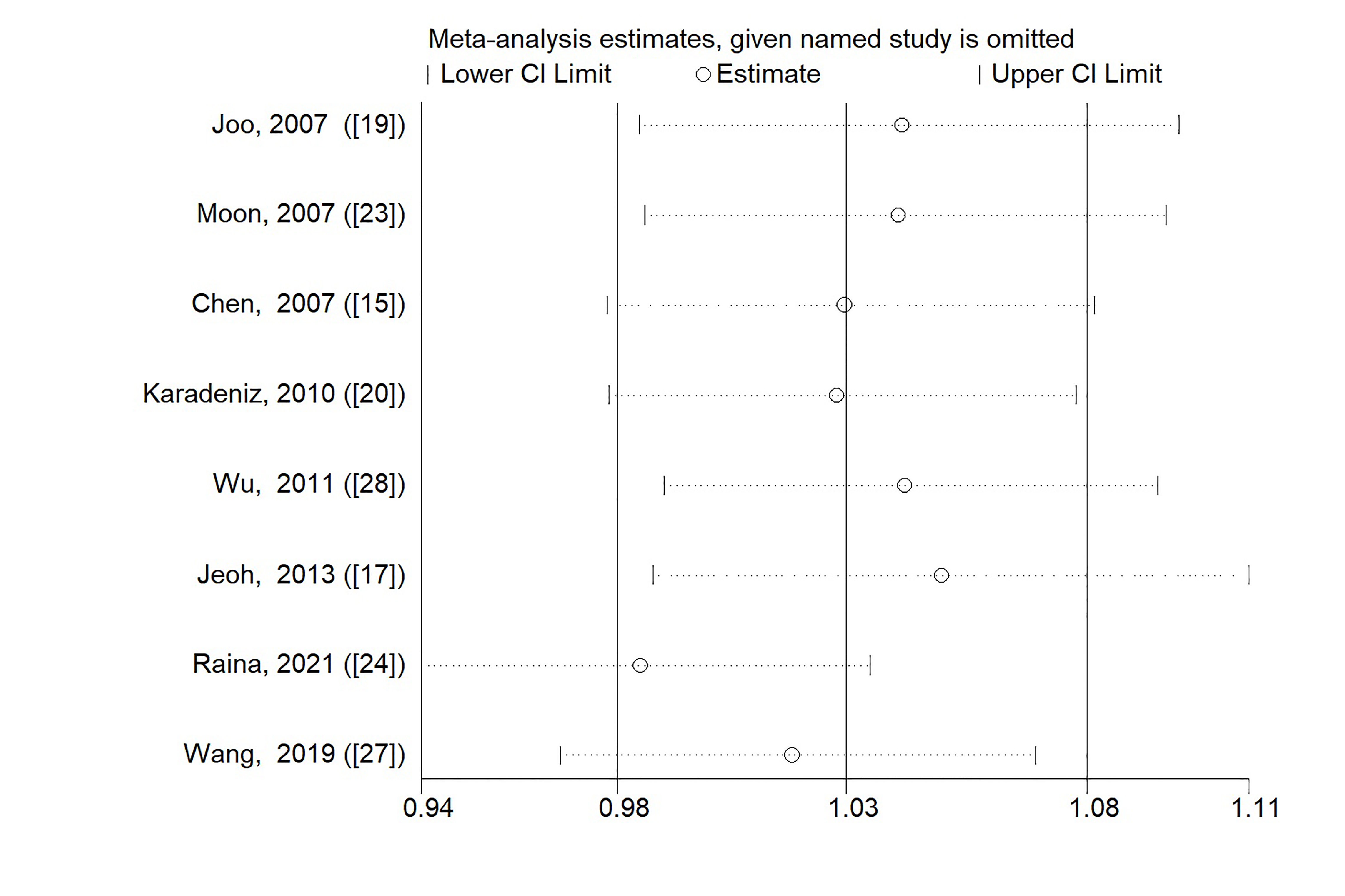

Supplement: Supplementary file 1 — Additional file 1: Figure S1. Forest plot of T2DM risk with the dominant model (GG+GA vs. AA) (T2DM vs. healthy control) of the MCP-1 rs1024611 polymorphism. (A) for the overall populations and (B) genotyping method; (C) age- and sex- adjusted; (D) and comorbid chronic disease subgroups. Figure S2. Forest plot of DN risk with the dominant model (GG+GA vs. AA) (DN vs. healthy control) of the MCP-1 rs1024611 polymorphism. (A) for the overall populations and (B) genotyping method; (C) age- and sex- adjusted; (D) and comorbid chronic disease subgroups. Figure S3. Forest plot of DN risk with the dominant model (GG+GA vs. AA) (DN vs. T2DM) of the MCP-1 rs1024611 polymorphism. (A) for the overall populations and (B) genotyping method; (C) age- and sex- adjusted; (D) and comorbid chronic disease subgroups. Figure S4. Sensitivity analysis via deletion of each individual study (T2DM vs. healthy control). (A) for GG+GA vs. AA and (B) GG vs.GA + AA; (C) GG vs. AA; (D) GG vs. GA; (E) and G vs. A models. Figure S5. Sensitivity analysis via deletion of each individual study (DN vs. healthy control). (A) for GG+GA vs. AA and (B) GG vs.GA + AA; (C) GG vs. AA; (D) GG vs. GA; (E) and G vs. A models. Figure S6. Sensitivity analysis via deletion of each individual study (DN vs. T2DM). (A) for GG+GA vs. AA and (B) GG vs.GA + AA; (C) GG vs. AA; (D) GG vs. GA; (E) and G vs. A models. Table S1. The comprehensive search strategies for different databases. Table S2. Main characteristic of included observational studies evaluating the relationship between the MCP-1 rs1024611 polymorphism and DN/T2DM risk. Table S3. Meta-analysis of the association between the MCP-1 rs1024611polymorphism and T2DM risk (T2DM vs. healthy control). Table S4. Meta-analysis of the association between the MCP-1 rs1024611polymorphism and DN risk (DN vs. healthy control). Table S5. Meta-analysis of the association between the MCP-1 rs1024611polymorphism and DN risk (DN vs. T2DM). [file 12902_2023_1514_MOESM1_ESM.zip › Figure S6/Figure S6A.jpg]

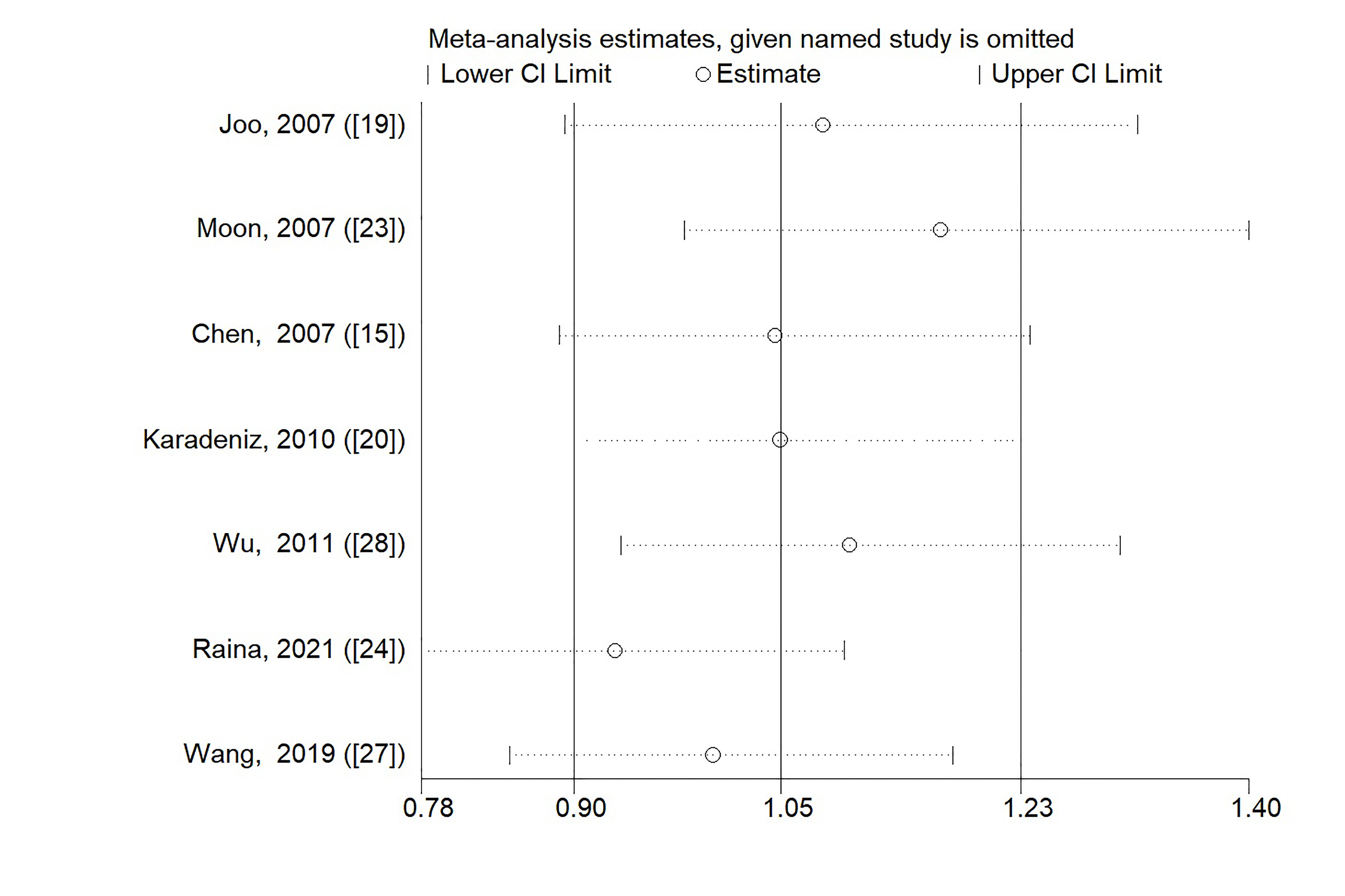

Supplement: Supplementary file 1 — Additional file 1: Figure S1. Forest plot of T2DM risk with the dominant model (GG+GA vs. AA) (T2DM vs. healthy control) of the MCP-1 rs1024611 polymorphism. (A) for the overall populations and (B) genotyping method; (C) age- and sex- adjusted; (D) and comorbid chronic disease subgroups. Figure S2. Forest plot of DN risk with the dominant model (GG+GA vs. AA) (DN vs. healthy control) of the MCP-1 rs1024611 polymorphism. (A) for the overall populations and (B) genotyping method; (C) age- and sex- adjusted; (D) and comorbid chronic disease subgroups. Figure S3. Forest plot of DN risk with the dominant model (GG+GA vs. AA) (DN vs. T2DM) of the MCP-1 rs1024611 polymorphism. (A) for the overall populations and (B) genotyping method; (C) age- and sex- adjusted; (D) and comorbid chronic disease subgroups. Figure S4. Sensitivity analysis via deletion of each individual study (T2DM vs. healthy control). (A) for GG+GA vs. AA and (B) GG vs.GA + AA; (C) GG vs. AA; (D) GG vs. GA; (E) and G vs. A models. Figure S5. Sensitivity analysis via deletion of each individual study (DN vs. healthy control). (A) for GG+GA vs. AA and (B) GG vs.GA + AA; (C) GG vs. AA; (D) GG vs. GA; (E) and G vs. A models. Figure S6. Sensitivity analysis via deletion of each individual study (DN vs. T2DM). (A) for GG+GA vs. AA and (B) GG vs.GA + AA; (C) GG vs. AA; (D) GG vs. GA; (E) and G vs. A models. Table S1. The comprehensive search strategies for different databases. Table S2. Main characteristic of included observational studies evaluating the relationship between the MCP-1 rs1024611 polymorphism and DN/T2DM risk. Table S3. Meta-analysis of the association between the MCP-1 rs1024611polymorphism and T2DM risk (T2DM vs. healthy control). Table S4. Meta-analysis of the association between the MCP-1 rs1024611polymorphism and DN risk (DN vs. healthy control). Table S5. Meta-analysis of the association between the MCP-1 rs1024611polymorphism and DN risk (DN vs. T2DM). [file 12902_2023_1514_MOESM1_ESM.zip › Figure S6/Figure S6B.jpg]

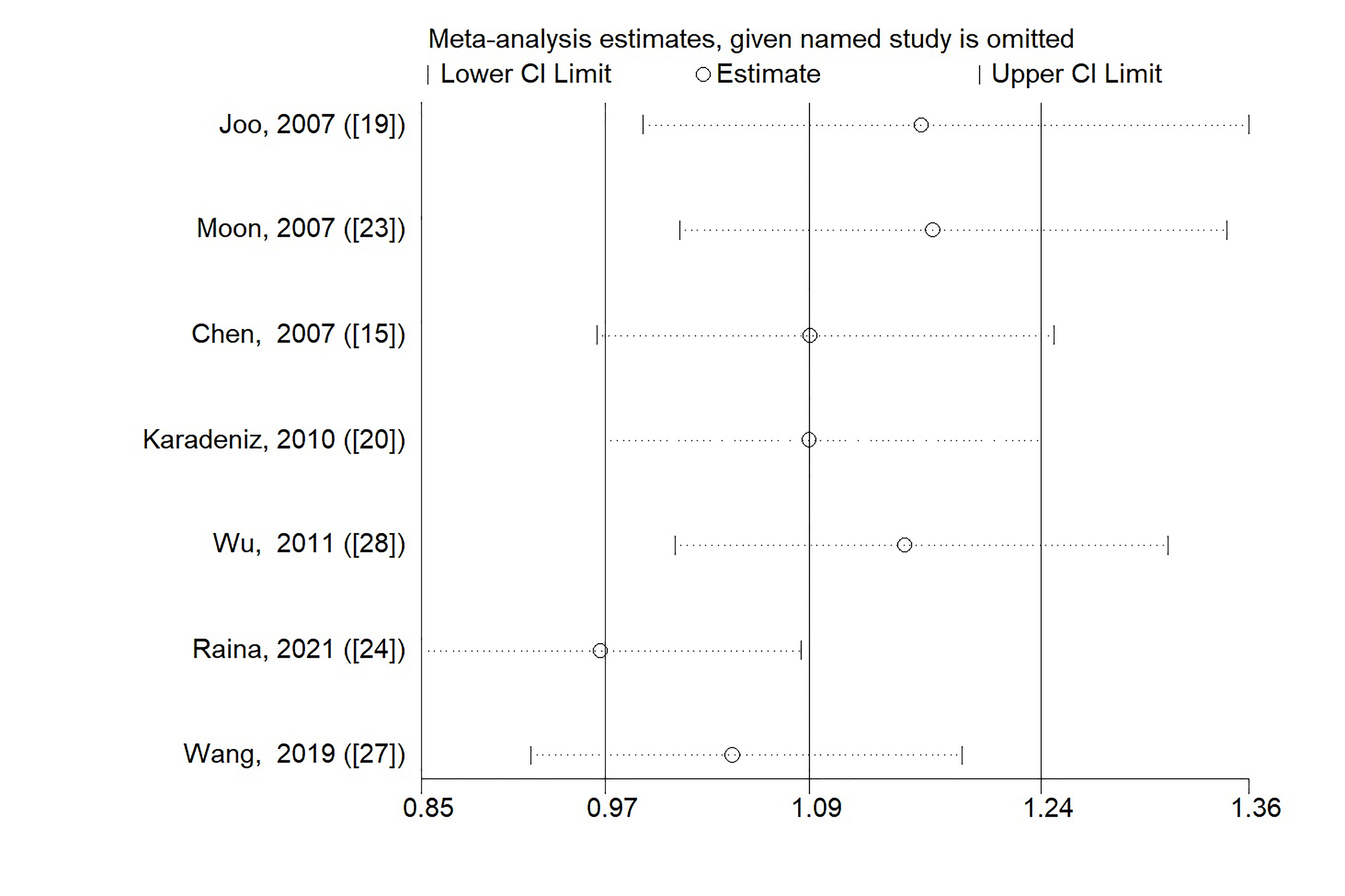

Supplement: Supplementary file 1 — Additional file 1: Figure S1. Forest plot of T2DM risk with the dominant model (GG+GA vs. AA) (T2DM vs. healthy control) of the MCP-1 rs1024611 polymorphism. (A) for the overall populations and (B) genotyping method; (C) age- and sex- adjusted; (D) and comorbid chronic disease subgroups. Figure S2. Forest plot of DN risk with the dominant model (GG+GA vs. AA) (DN vs. healthy control) of the MCP-1 rs1024611 polymorphism. (A) for the overall populations and (B) genotyping method; (C) age- and sex- adjusted; (D) and comorbid chronic disease subgroups. Figure S3. Forest plot of DN risk with the dominant model (GG+GA vs. AA) (DN vs. T2DM) of the MCP-1 rs1024611 polymorphism. (A) for the overall populations and (B) genotyping method; (C) age- and sex- adjusted; (D) and comorbid chronic disease subgroups. Figure S4. Sensitivity analysis via deletion of each individual study (T2DM vs. healthy control). (A) for GG+GA vs. AA and (B) GG vs.GA + AA; (C) GG vs. AA; (D) GG vs. GA; (E) and G vs. A models. Figure S5. Sensitivity analysis via deletion of each individual study (DN vs. healthy control). (A) for GG+GA vs. AA and (B) GG vs.GA + AA; (C) GG vs. AA; (D) GG vs. GA; (E) and G vs. A models. Figure S6. Sensitivity analysis via deletion of each individual study (DN vs. T2DM). (A) for GG+GA vs. AA and (B) GG vs.GA + AA; (C) GG vs. AA; (D) GG vs. GA; (E) and G vs. A models. Table S1. The comprehensive search strategies for different databases. Table S2. Main characteristic of included observational studies evaluating the relationship between the MCP-1 rs1024611 polymorphism and DN/T2DM risk. Table S3. Meta-analysis of the association between the MCP-1 rs1024611polymorphism and T2DM risk (T2DM vs. healthy control). Table S4. Meta-analysis of the association between the MCP-1 rs1024611polymorphism and DN risk (DN vs. healthy control). Table S5. Meta-analysis of the association between the MCP-1 rs1024611polymorphism and DN risk (DN vs. T2DM). [file 12902_2023_1514_MOESM1_ESM.zip › Figure S6/Figure S6C.jpg]

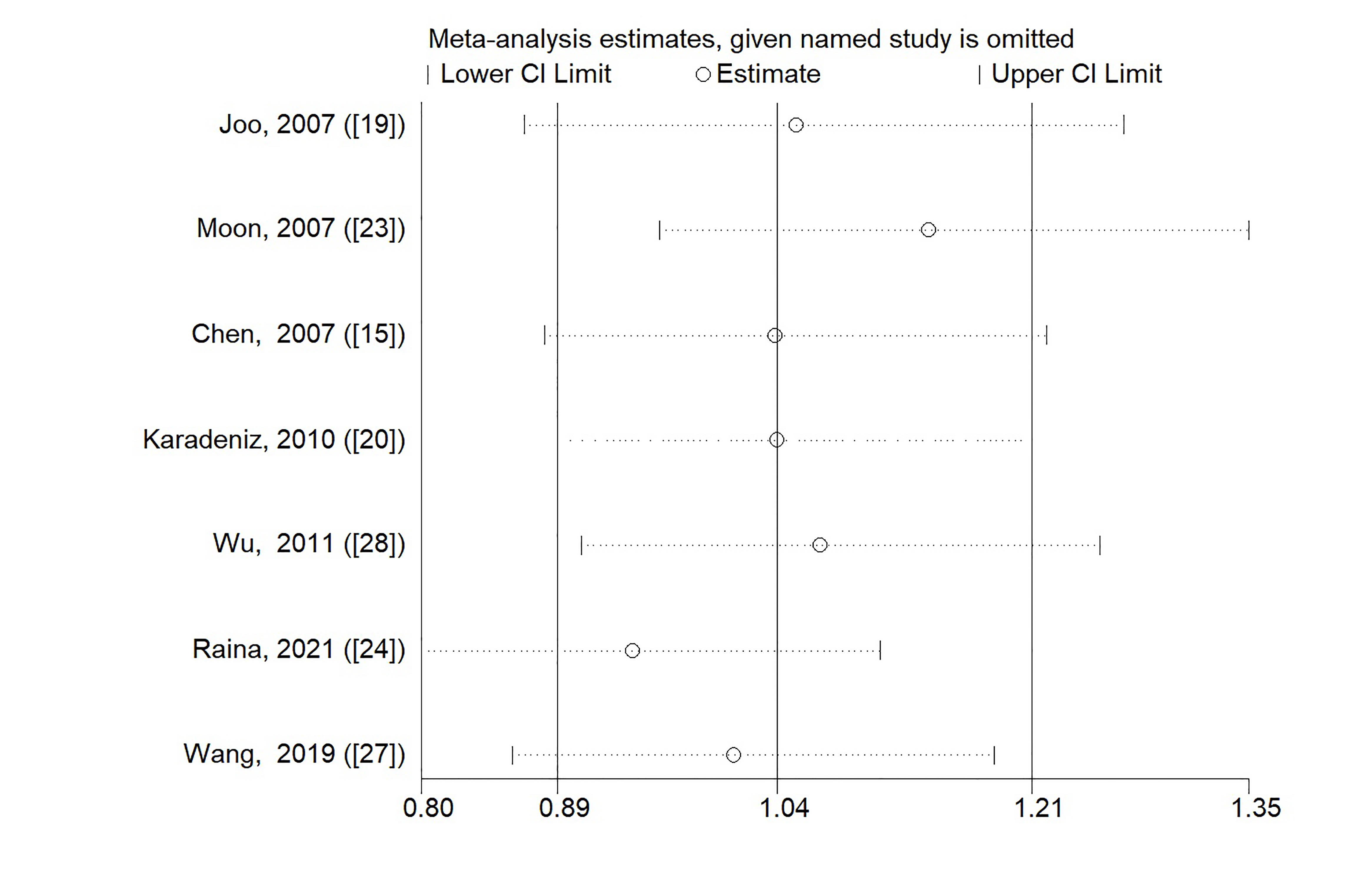

Supplement: Supplementary file 1 — Additional file 1: Figure S1. Forest plot of T2DM risk with the dominant model (GG+GA vs. AA) (T2DM vs. healthy control) of the MCP-1 rs1024611 polymorphism. (A) for the overall populations and (B) genotyping method; (C) age- and sex- adjusted; (D) and comorbid chronic disease subgroups. Figure S2. Forest plot of DN risk with the dominant model (GG+GA vs. AA) (DN vs. healthy control) of the MCP-1 rs1024611 polymorphism. (A) for the overall populations and (B) genotyping method; (C) age- and sex- adjusted; (D) and comorbid chronic disease subgroups. Figure S3. Forest plot of DN risk with the dominant model (GG+GA vs. AA) (DN vs. T2DM) of the MCP-1 rs1024611 polymorphism. (A) for the overall populations and (B) genotyping method; (C) age- and sex- adjusted; (D) and comorbid chronic disease subgroups. Figure S4. Sensitivity analysis via deletion of each individual study (T2DM vs. healthy control). (A) for GG+GA vs. AA and (B) GG vs.GA + AA; (C) GG vs. AA; (D) GG vs. GA; (E) and G vs. A models. Figure S5. Sensitivity analysis via deletion of each individual study (DN vs. healthy control). (A) for GG+GA vs. AA and (B) GG vs.GA + AA; (C) GG vs. AA; (D) GG vs. GA; (E) and G vs. A models. Figure S6. Sensitivity analysis via deletion of each individual study (DN vs. T2DM). (A) for GG+GA vs. AA and (B) GG vs.GA + AA; (C) GG vs. AA; (D) GG vs. GA; (E) and G vs. A models. Table S1. The comprehensive search strategies for different databases. Table S2. Main characteristic of included observational studies evaluating the relationship between the MCP-1 rs1024611 polymorphism and DN/T2DM risk. Table S3. Meta-analysis of the association between the MCP-1 rs1024611polymorphism and T2DM risk (T2DM vs. healthy control). Table S4. Meta-analysis of the association between the MCP-1 rs1024611polymorphism and DN risk (DN vs. healthy control). Table S5. Meta-analysis of the association between the MCP-1 rs1024611polymorphism and DN risk (DN vs. T2DM). [file 12902_2023_1514_MOESM1_ESM.zip › Figure S6/Figure S6D.jpg]

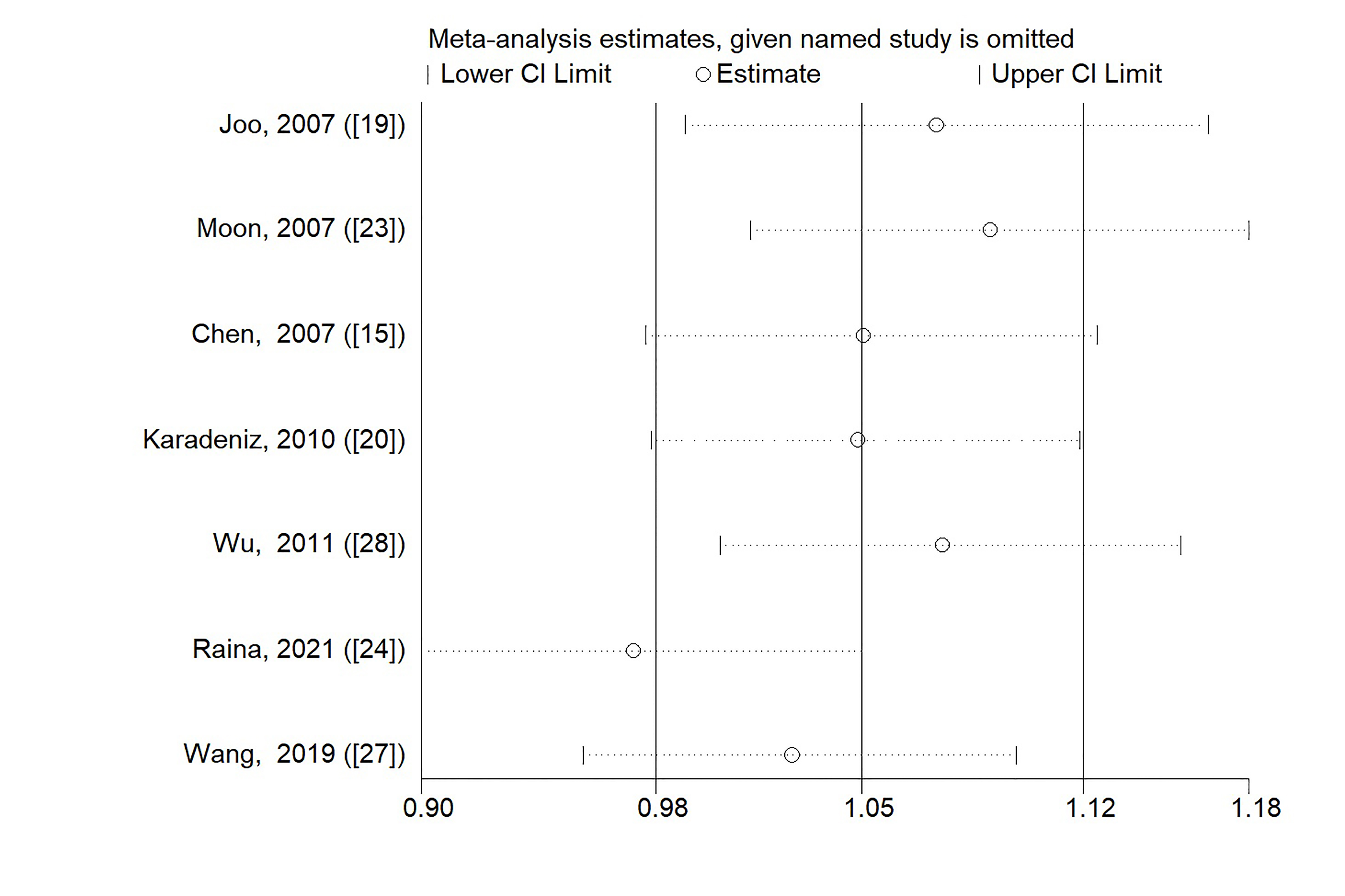

Supplement: Supplementary file 1 — Additional file 1: Figure S1. Forest plot of T2DM risk with the dominant model (GG+GA vs. AA) (T2DM vs. healthy control) of the MCP-1 rs1024611 polymorphism. (A) for the overall populations and (B) genotyping method; (C) age- and sex- adjusted; (D) and comorbid chronic disease subgroups. Figure S2. Forest plot of DN risk with the dominant model (GG+GA vs. AA) (DN vs. healthy control) of the MCP-1 rs1024611 polymorphism. (A) for the overall populations and (B) genotyping method; (C) age- and sex- adjusted; (D) and comorbid chronic disease subgroups. Figure S3. Forest plot of DN risk with the dominant model (GG+GA vs. AA) (DN vs. T2DM) of the MCP-1 rs1024611 polymorphism. (A) for the overall populations and (B) genotyping method; (C) age- and sex- adjusted; (D) and comorbid chronic disease subgroups. Figure S4. Sensitivity analysis via deletion of each individual study (T2DM vs. healthy control). (A) for GG+GA vs. AA and (B) GG vs.GA + AA; (C) GG vs. AA; (D) GG vs. GA; (E) and G vs. A models. Figure S5. Sensitivity analysis via deletion of each individual study (DN vs. healthy control). (A) for GG+GA vs. AA and (B) GG vs.GA + AA; (C) GG vs. AA; (D) GG vs. GA; (E) and G vs. A models. Figure S6. Sensitivity analysis via deletion of each individual study (DN vs. T2DM). (A) for GG+GA vs. AA and (B) GG vs.GA + AA; (C) GG vs. AA; (D) GG vs. GA; (E) and G vs. A models. Table S1. The comprehensive search strategies for different databases. Table S2. Main characteristic of included observational studies evaluating the relationship between the MCP-1 rs1024611 polymorphism and DN/T2DM risk. Table S3. Meta-analysis of the association between the MCP-1 rs1024611polymorphism and T2DM risk (T2DM vs. healthy control). Table S4. Meta-analysis of the association between the MCP-1 rs1024611polymorphism and DN risk (DN vs. healthy control). Table S5. Meta-analysis of the association between the MCP-1 rs1024611polymorphism and DN risk (DN vs. T2DM). [file 12902_2023_1514_MOESM1_ESM.zip › Figure S6/Figure S6E.jpg]
